# Preparation method shapes the recovery and ecological interpretation of DNA and RNA soil viral communities

**DOI:** 10.1038/s41467-026-74154-1

**Published:** 2026-07-28

**Authors:** Josué A. Rodríguez-Ramos, Amy E. Zimmerman, Ruonan Wu, Sheryl L. Bell, Trinidad D. Alfaro, Nicholas J. Reichart, Kirsten S. Hofmockel, William C. Nelson

**Affiliations:** 1 Earth and Biological Sciences Directorate, Pacific Northwest National Laboratory, Richland, WA USA; 2 Department of Agronomy, Iowa State University, Ames, IA USA

**Keywords:** Microbial ecology, Bioinformatics, Microbiology techniques

## Abstract

Deciphering viral ecology in soils is challenging due to soil’s high physicochemical and microbial community complexity. To enhance detection of DNA and RNA viruses, we applied different preparation methods to soils collected from a grassland field experiment. Analyses included metagenomics and metatranscriptomics of size-fractionated extracellular viruses, total soil metagenomics and metatranscriptomics, total soil metatranscriptomics with polyadenylation enrichment, and metagenomics of bacteria/archaea as well as eukaryote-enriched samples. DNA viromes outperformed total soil metagenomes in viral detection and quality. Contrastingly, RNA viromes and total soil metatranscriptomes performed similarly for viral recovery, though RNA viromes yielded higher-quality genomes. Together, our results highlight how different preparation methods can influence the recovery and quality of DNA and RNA vOTUs. Further, we demonstrate the power of different methods in identifying distinct viral communities with unique host predictions, which in turn can have significant implications for ecological investigations related to interkingdom interactions.

## Abstract (web summary)

Profiling soil viral communities is challenging due to the inherent complexity of soil samples. In this study, the authors show that soil sample preparation strongly shapes which DNA and RNA viruses are detected, which can, in turn, impact ecological implications of soil viral community analyses.

## Introduction

In soils, viral communities are prevalent, with estimates varying between 10^7^−10^10^ viruses per gram of soil^1–3^, and are recognized for their impacts on biogeochemical cycling^4,5^. Theoretical estimates report that viruses may transform ~1.2 Pg of terrestrial prokaryotic carbon per year globally, assuming a 15% virus-mediated mortality rate^6^. Experimental assessments in soils show that viruses can alter the fate of dissolved organic carbon and nitrogen^7^ and influence the dissolved organic matter and CO_2_ released into the atmosphere^8^. Nonetheless, understanding and constraining viral ecology is an ongoing challenge due to the complex interactions between the kingdoms of life (i.e., fungi, bacteria, archaea, etc.) that govern the soil biosphere.

From a methodological perspective, disentangling soil microbial diversity into sequence-based representation can be challenging. Viruses are incredibly diverse, encompassing different genomic compositions (DNA vs. RNA), reproductive strategies (lytic, lysogenic, chronic, etc.), and host communities, all of which result in meaningful differences at the sequence level that can be difficult to fully capture^9^. Similarly, bacteria and archaea can have highly variable abundances in natural systems, resulting in underrepresentation of rare organisms in genomic databases^10^. Finally, organisms like fungi have low DNA concentrations and their cell walls are difficult to lyse, resulting in lower DNA yields that limit representation and complicate scientific interpretations^11^. We note that aside from methodological constraints, the high number of possible interactions between soil microbiomes also complicates the interpretation of microbial data. Ultimately, the complexity of soil makes soil microbiomes difficult to characterize.

To mitigate these issues, researchers have developed preparation methods to enrich for specific pools of organisms prior to metagenomic (or metatranscriptomic) sequencing. For example, flotation-based approaches are effective for isolating hyphae and roots^12^. Density-based gradients (e.g., Nycodenz) separate target groups (i.e., bacterial, archaeal, viral, etc.) from others, thus enriching their quantities prior to DNA extraction^13^. Size fractionation for free viral particles prior to DNA or RNA extractions is an alternative to the mining of total metagenomes for viral genomes^14–16^. Polyadenylation of RNA is common in eukaryotes, making poly-dT-based enrichment methods an efficient screen for eukaryotic activity^17^, and their viral counterparts^18^. Together, these methods have helped reveal the extent of soil biodiversity and uncover the ecological roles of viral^15^, bacterial/archaeal^19^, and fungal^20^ communities in soil. However, key methodological knowledge gaps remain regarding viral communities: 1) these multi-kingdom enrichment methods have not been extensively assessed for their ability to identify viruses that are associated with different biological kingdoms, particularly in soils, and 2) RNA virus data from fractionated virus methods have not been compared to whole soil RNA methods.

Viral DNA and RNA metagenomics and metatranscriptomics have unearthed a massive diversity of soil viruses which are continuing to increase our knowledge of their global distributions and ecological functions^5,21–23^. These discoveries are shedding light on the adaptability and resilience of viral communities under varying environmental conditions. For instance, viral community compositions can shift in response to changes in moisture regimes, which highlights the dynamic nature of soil viromes^24–26^. Such shifts not only influence the viral populations themselves but also impact the broader microbial communities they interact with. However, additional studies examining different soil types are needed to understand how viral and microbial communities change across soil characteristics like texture and moisture level.

**Fig. 1:**
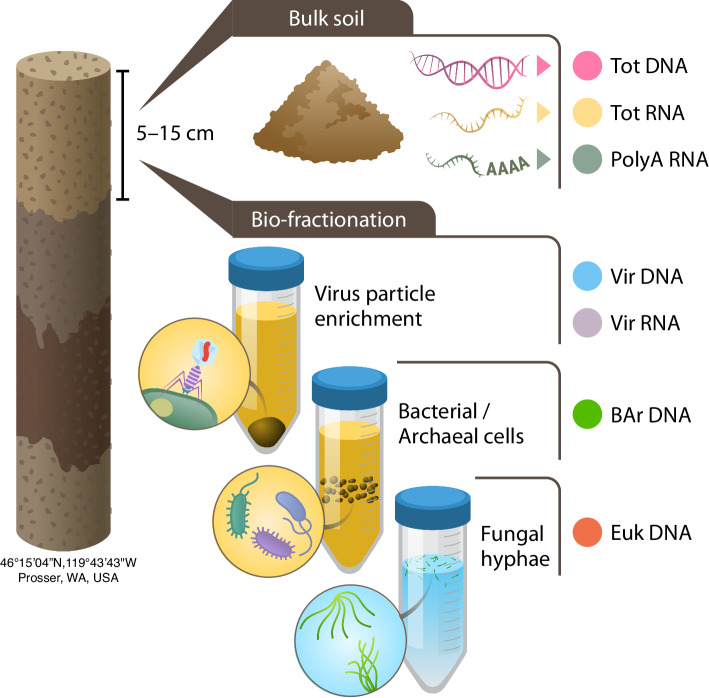
Using parallel preparation methods to unearth soil viral diversity. Conceptual diagram of the preparation methods employed to yield the 7 methods tested in this manuscript. From the top 5–15 cm of soil, a single core was collected and 7 parallel sequencing protocols were employed: Tot DNA (i.e., total metagenomes), Tot RNA (i.e., total metatranscriptomes), PolyA RNA (i.e., poly(A) enriched bulk metatranscriptomes), Vir DNA (i.e., DNA viral metagenomes), Vir RNA (i.e., RNA viral metatranscriptomes), BAr DNA (i.e., Nycodenz bacterial and archaeal enrichment), and Euk DNA (i.e., fungal and plant flotations). For more details on how each preparation was collected, see Methods.

Here, we sampled a single site with 7 plots (19.5 m^2^) to 1) evaluate the impact of sample preparation methods on the identification of distinct viral communities, and 2) understand how different preparation methods can impact our understanding of viral community responses to soil moisture in an irrigated (managed) arid grassland. To address these questions, we applied multiple fractionation approaches to enrich distinct subsets of the soil microbiome e.g., viruses (Vir DNA and RNA), bacteria and archaea (BAr DNA), and eukaryotes (Euk DNA), as well as bulk soil methods e.g., total DNA (Tot DNA), total RNA (Tot RNA), and polyadenylation enriched total RNA (PolyA RNA) (Fig. 1). We hypothesized that by pairing multiple preparation approaches with multi-omics, we would improve sensitivity of viruses pertaining to less studied viral communities in sequence data (i.e., fungi, archaea).

## Results

### Parallel soil preparation methods yield unique vOTU profiles

From multiple preparation methods (Vir DNA, Tot DNA, BAr DNA, Euk DNA, Vir RNA, Tot RNA, PolyA RNA), we generated a total of 46 metagenomes and 39 metatranscriptomes from 7 experimental plots representing a moisture gradient in our arid grassland field site (Fig. 1, Fig. S1, Data S1). All metagenomic and metatranscriptomic datasets were assembled individually and screened for vOTUs (Fig. S2). This resulted in a database of 28,071 viral OTUs (vOTUs) clustered within individual methods (see Methods, Data S2). Specifically, the database was composed of 19,769 DNA vOTUs and 8302 ssRNA vOTUs. We note that we focused only on ssRNA vOTUs to assess RNA virus transcript abundance patterns (see Methods, Fig. S2).

vOTU recovery (i.e., the extent to which vOTUs ≥10 kb can be assembled from a preparation) was different across preparation methods for both DNA and RNA viruses. The majority (71%) of the DNA vOTUs came from Vir DNA sequencing (14,124 vOTUs). On the other hand, the number of RNA vOTUs were similar between the Tot RNA (4035 vOTUs) and Vir RNA sequence sets (3892 vOTUs) (Fig. S2). Methods yielding the most viral contigs were (in descending order): Vir DNA, Euk DNA, Tot DNA, and BAr DNA for DNA vOTUs, and Tot RNA, Vir RNA, and PolyA RNA for RNA vOTUs. Notably, accumulation curves all showed steady increases in the number of viruses identified with each additional sample, suggesting a large diversity of viruses remains to be identified (Fig. S3A). Nonetheless, we note that both Vir DNA and RNA methods had the largest proportions of mapped reads to vOTUs, confirming that virome methods effectively enrich for both DNA and RNA viral communities, more so than other methods (Fig. S3B).

**Fig. 2:**
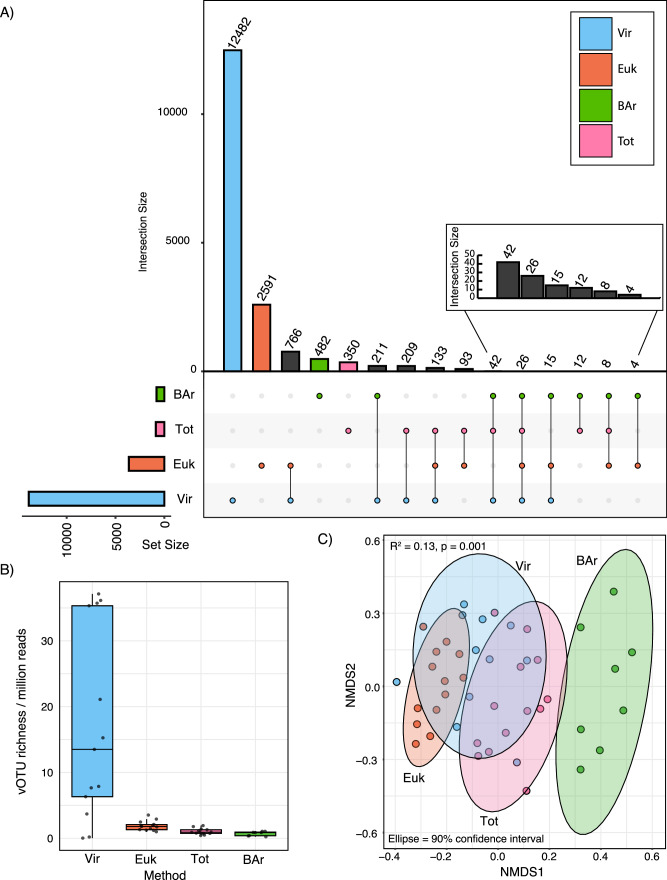
DNA viral preparation methods recover and detect non-overlapping DNA viral OTUs. Colors show different preparation methods. **A** Upset plot shows the intersection of DNA viral OTUs across preparation methods by means of recovery (i.e., are clustered at 95% ANI across 85% of the shortest contig across different methods). Vertical bars show the size of overlap (or number of unique vOTUs), while horizontal bars show set size. **B** Boxplots show the total number of DNA vOTUs, normalized for sample read depth, across methods by means of detection (i.e., reads from each method mapped to the viral reference database). Boxes represent the interquartile range (IQR) with the median shown as the center line; whiskers extend to the most extreme values within 1.5 × IQR of the first and third quartiles (Tukey method). All preparations have 13 biological replicates except for BAr DNA (8 samples). **C** NMDS of vOTU presence or absence as determined by means of detection across all preparation methods. R^2^ and *p* value is calculated using adonis2 (PERMANOVA), and ellipses show a 90% confidence interval for the Bray-Curtis dissimilarity of each group.

**Fig. 3:**
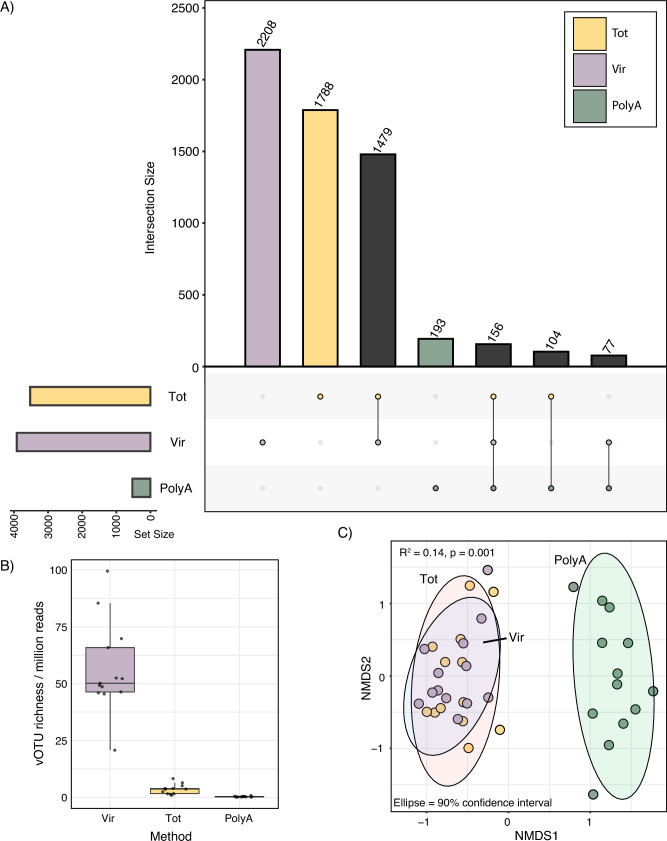
RNA viral preparation methods recover and detect non-overlapping RNA viral OTUs. Colors show different preparation methods. **A** Upset plot shows the intersection of RNA viral OTUs across preparation methods by means of recovery (i.e., are clustered at 95% ANI across 85% of the shortest contig across different methods). Vertical bars show the size of overlap (or number of unique vOTUs), while horizontal bars show set size. **B** Boxplots show the total number of RNA vOTUs, normalized for sample read depth, across methods by means of detection (i.e., reads from each method mapped to the viral reference database). Boxes represent the interquartile range (IQR) with the median shown as the center line; whiskers extend to the most extreme values within 1.5 × IQR of the first and third quartiles (Tukey method). All preparations have 13 biological replicates. **C** NMDS of vOTU presence or absence as determined by means of detection across all preparation methods. R^2^ and *p* value are calculated using adonis2 (PERMANOVA), and ellipses show a 90% confidence interval for the Bray-Curtis dissimilarity of each group.

To assess viral recovery efficiency, we examined the distribution of assembled vOTUs across methods by using the sequence similarity clustering results of globally clustered (i.e., clustered across methods) DNA and RNA databases, hereafter referred to as our consolidated database of 17,590 DNA vOTUs and 6005 RNA vOTUs (23,595 vOTUs total). Essentially, this allows us to determine whether any individual method is capable of the assembly of a virus (i.e., by de-novo assembly). Because sequencing depth and the percent fraction of viral reads (i.e., the enrichment of viral particles) per sample are themselves outcomes of each preparation method, we did not rarefy our reads to a common sequencing depth; instead we opted to normalize the number of vOTUs detected to the total sequencing depth from each method^21^. Each method yielded more unique vOTUs than shared (Figs. 2A and 3A). Notably, 44% of total overlapping vOTUs were shared between Tot Soil RNA and Vir RNA (1479 vOTUs), and 23% between the Vir DNA and Euk DNA (766 vOTUs).

To determine how well each method performed at viral detection (i.e., the ability to detect a vOTU in a preparation by the mapping of reads to a reference vOTU database such as assembled viral scaffolds or a tailored vOTU database), we mapped the individual reads from each preparation method to the consolidated database (17,590 DNA vOTUs, 6005 RNA vOTUs). By combining viruses from all methods into a single, consolidated vOTU database, we mitigate some of the deficiencies of individual assemblies (e.g., fragmented contigs) and improve our ability to detect shared viruses across methods via the mapping of 151 bp reads. In this way, we can assess whether the apparent differences in recovery (Figs. 2A and 3A) primarily reflect true enrichment of vOTUs or are influenced by incomplete contig reconstruction in some methods. Mirroring the recovery-based approach comparisons above, DNA and RNA viral preparations consistently had a higher detection of viral contigs than other methods after accounting for the overall sequencing depth of each method (Figs. 2B and 3B). We note, however, that Vir RNA and Tot RNA had equivalent total number of vOTUs detected without accounting for total sequencing.

We used a binary presence/absence matrix of viral contigs to determine whether the detection of each vOTU per preparation was unique. For DNA vOTUs, Euk, Tot, and BAr methods contained significantly different viral communities (Fig. 2C). Further, the Vir DNA method showed overlap with the Euk and Tot methods but not the BAr method. For RNA vOTUs, the composition of Tot and Vir vOTU communities were strongly overlapping (Fig. 3C). Contrastingly, the PolyA method detected significantly different (*p* ≤ 0.05) vOTUs. Ultimately, these results show 1) each DNA preparation method (besides Vir DNA) recovered and detected unique sets of vOTUs, 2) Vir DNA recovery had the largest overlap with other DNA methods, and 3) RNA virome and total metatranscriptomes were similar. More research comparing informatics techniques like co-assemblies and multiple assemblers (i.e., metaSPAdes^27^) are needed to expand on these results. However, given the similarity between results from recovery-based and detection-based approaches, further analysis focused on recovery-based assessments of the different preparation methods.

### Vir DNA and RNA recovered a higher number of longer and higher quality vOTUs than other preparation methods

To assess the impact of each preparation method on the recovery of more complete, higher-quality viruses, we compared the lengths of vOTUs that were recovered in two or more methods (Fig. S4A). For the 3,864 DNA viruses present in at least two preparation methods, Vir DNA sequencing yielded the longest viral contigs for 47% of viruses, followed by Euk DNA (35%), Tot DNA (10%), and BAr DNA (8%). Of the 4043 RNA vOTUs shared in at least two methods, Vir RNA recovered the longest contigs for 77% of shared viruses, followed by Tot RNA (19%) and PolyA RNA (4%).

We also wanted to evaluate the overall quality of the viral contigs recovered across methods (Fig. S4B). Specifically, we calculated which method had the best quality representative within a viral cluster by CheckV quality (complete, high-quality, medium-quality, low-quality). Like the results for viral lengths, Vir DNA had the largest proportion of higher quality vOTUs (44%), followed by Euk DNA (36%), Tot DNA (12%) and BAr DNA (8%). Similarly, for RNA vOTUs, Vir RNA accounted for a higher quality representative in 69% of cases followed by RNA Bulk (27%) and PolyA RNA (4%). Together, these results highlight that both DNA and RNA Vir methods consistently recovered longer, more complete genomes for both DNA and ssRNA viruses.

### Most DNA and ssRNA viral families are recovered by all preparation methods

**Fig. 4:**
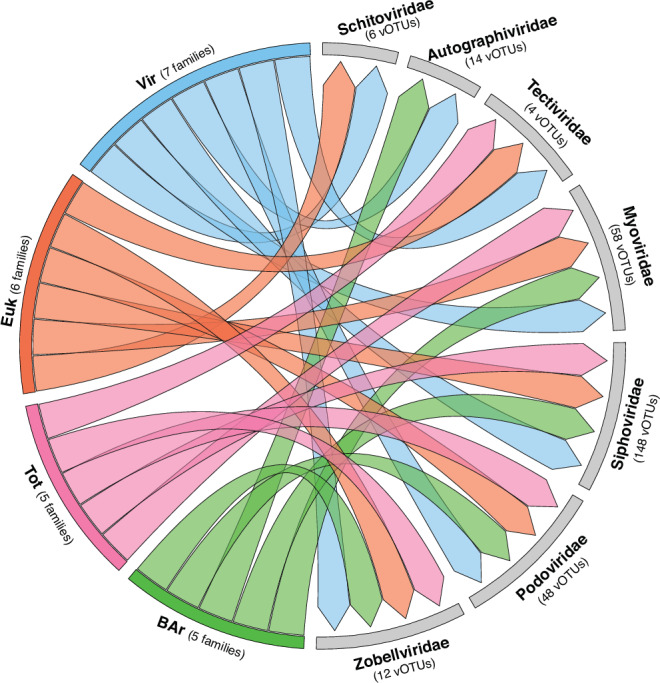
DNA virus preparation methods can select for family-level taxonomic assignments. vContact2 output of genera-level clustering between viruses from this study and RefSeq v211. Each chord represents the detection of a viral family by a preparation method for the total number of DNA vOTUs that could be taxonomically classified (i.e., clustered to the RefSeq database (289 vOTUs).

While vOTUs are usually the target unit of interest in viral studies, it is not always clear whether they represent functionally distinct populations, particularly with regards to RNA viruses. As such, to understand whether there were differences in viral recovery between methods, we also examined viral overlaps at higher taxonomic levels. For DNA vOTUs, vContact2 was used to compare the clustered dataset to the RefSeq v211 database (Fig. 4). 49% (9604 vOTUs) of DNA vOTUs were able to be confidently placed into genus-level clusters, with the remaining 51% of DNA vOTUs being either singletons (44%) or unable to be confidently assigned to a single genus-level cluster (e.g., overlap clusters, 7%). Only 3% (289 vOTUs) of genus-clustered vOTUs clustered to the RefSeq database (i.e., could be taxonomically classified).

Of the viruses that are taxonomically classified, only Vir DNA recovered all classified viral families. *Schitoviridae* were only recovered in the Vir DNA and Euk DNA, while *Autographviridae* were only recovered in the Vir DNA and BAr DNA. *Tectiviridae* were not recovered by BAr DNA. *Siphoviridae*, *Myoviridae*, *Podoviridae*, and *Zobellviridae* were recovered across all methods. We highlight that while all methods recovered most of the classified Families in our dataset, only a very small proportion of vOTUs are confidently assigned taxonomy (289 vOTUs). In fact, of the 9604 genera-level clusters present in our databases, 40% of those were unique within individual methods (Data S3), suggesting some degree of taxonomic uniqueness per method.

**Fig. 5:**
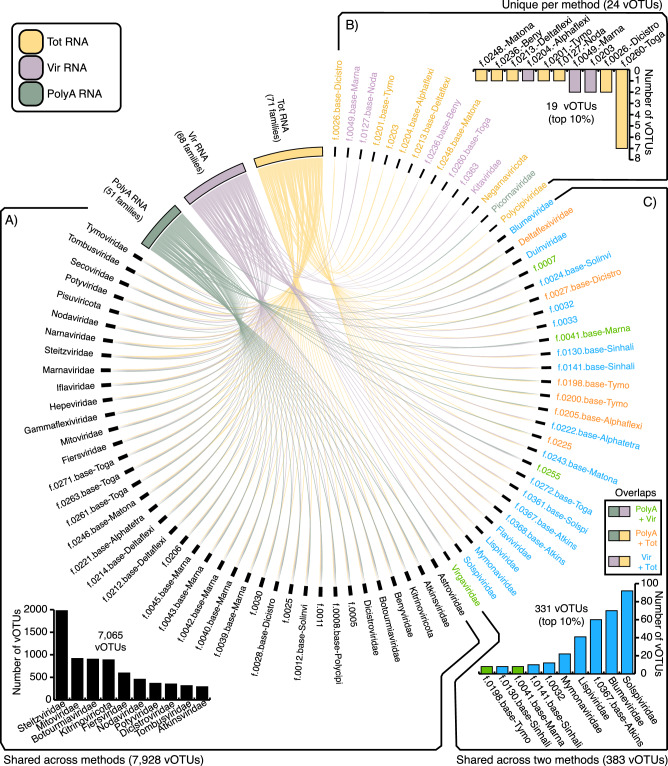
RNA vOTU families are generally widespread across preparation methods. A Chord diagram of ssRNA (positive or negative sense genome types) taxonomic overlap at the family level. show the top 10 families that overlap in either (**A**) all methods, (**B**) two methods, or (**C**) one method.

For RNA vOTUs, classification was based on phylogenetic analyses of the RNA-dependent RNA polymerases (RdRp). Overall, 8302 RdRps were recovered from the RNA vOTUs and classified into 82 families (Fig. 5). 49% of RNA families were recovered by every method and accounted for 95% of RdRps in our database (7928 RdRPs). 33% of viral families (383 RdRps, or 5% of all RdRps) were recovered by only 2 methods, the majority of which (63%) were shared between Tot RNA and Vir RNA. Finally, 18% of families ( < 1% of all RdRps) were only recovered in a single data stream, with 53% of these recovered by Tot RNA, and 40% by Vir RNA. One family, *Picornaviridae*, was recovered solely in the PolyA RNA.

Together, these results highlight that 49% of taxonomically classifiable RNA viral families and 57% of classifiable DNA viral families are recovered regardless of preparation method, but that there are methods that can have less sensitivities to certain families like *Schitoviridae*, *Autographviridae*, and *Picornaviridae*. Notably, it contrasts the vOTU level results which showed viruses were mostly unique per preparation method. This could suggest that vOTUs may belong to distinct communities whose ecological uniqueness are aggregated at the family-level, and/or that high levels of viral community heterogeneity conceal the true total diversity and overlap of lower-abundance viruses^28^.

### Different methods enriched for different proportions of predicted host taxonomies

**Fig. 6:**
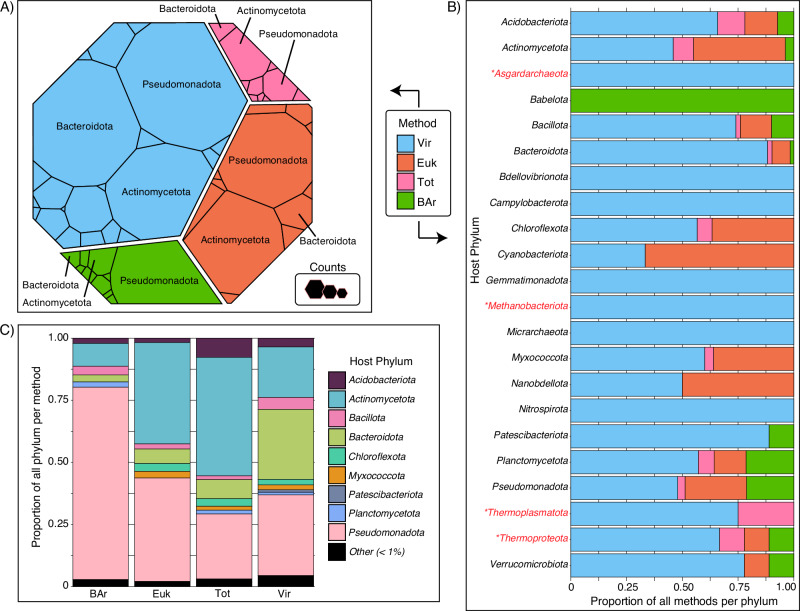
DNA virus-host predictions show method-specific host enrichment. **A** Different bacterial and archaeal phyla that were detected as hosts between each different method (colors), and the size of the shapes denotes the overall number of viruses that putatively infect each phylum. The top 3 phyla from each method are denoted by text. **B** Proportion that each method contributes to the total detectable host pool. Red text and asterisks indicate whether lineages are archaeal. **C** Phylum-level proportions of each host for different DNA methods for Phyla that accounted for at least 1% of the relative proportion of vOTUs identified.

In silico host prediction for DNA vOTUs successfully assigned putative hosts to 1317 vOTUs (out of 19,769 vOTUs) which encompassed 1213 bacterial and archaeal genomes from 28 unique phyla. The BAr DNA vOTU set had the highest proportion of host links of any method (153 host matches, 18% of BAr DNA vOTUs), followed by Euk DNA (336 host matches, 9% of Euk DNA), Tot DNA (65 host matches, 6% of Tot DNA vOTUs) and Vir DNA (625 host matches, 4% of Vir DNA vOTUs). The Vir DNA set had the largest total number of linked vOTUs (Fig. 6A). Notably, the predicted host profiles varied per preparation method, and only 32% of host assignments at the phyla level were detected across all four DNA datasets (*Acidobacteriota, Actinomycetota, Bacillota, Bacteroidota, Planctomycetota*, and *Pseudomonadota*).

The Vir DNA vOTU set linked to the widest variety of host phyla (21 phyla) and was the only method to assign hosts to *Asgardarchaeota*, *Bdellovibrionota*, *Campylobacterota*, *Gemmatimonadota*, *Methanobacteriota*, *Micrarchaeota*, and *Nitrospirota* (Fig. 6B). BAr DNA vOTUs linked to 10 different phyla and provided unique links to *Babelota*. Euk DNA and Tot DNA linked to 12 and 10 phyla, respectively, and were not assigned any unique host phyla. The proportions of hosts detected per method also varied widely (Fig. 6C). For example, Bar DNA had more assignments to *Pseudomonadota*, Euk DNA had more assignments to *Chloroflexota*, Tot DNA had more assignments to *Actinomycetota*, and Vir DNA had more assignments to *Bacteroidota*.

**Fig. 7:**
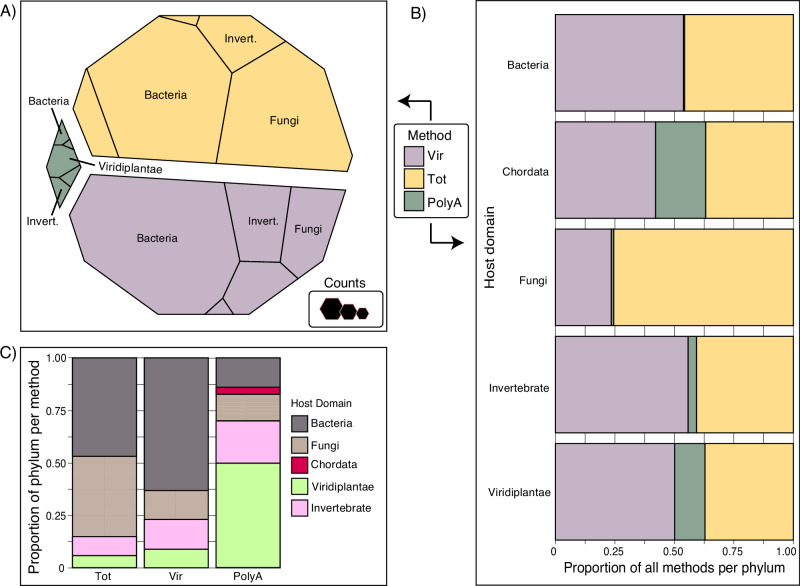
RNA virus-host predictions show method-specific host enrichment. **A** Different Domains that were detected as hosts between each different method (colors), and the size of the shapes denotes the overall number of viruses that putatively infect each Domain. The top 3 Domains from each method are denoted by text. **B** Proportion that each method contributes to the total detectable host pool. **C** Domain-level proportions of each host for different RNA methods for Phyla that accounted for at least 1% of the relative proportion of vOTUs identified.

RNA virus-host predictions were done with RNAVirHost^29^ at a higher-level taxonomic resolution per tool limitations. Of the 8302 RNA vOTUs analyzed, 5,781 (70%) were confidently assigned a putative host. Tot RNA had the highest number of assigned predictions (3,022, 75% of Tot RNA vOTUs), followed by Vir RNA (2,641, 68% of Vir vOTUs) and PolyA RNA (118, 31% of total PolyA vOTUs) (Fig. 7A). While Tot RNA and Vir RNA had more host assignments than PolyA RNA, all methods had at least one hit to each kingdom (Bacteria, Chordata, Fungi, Invertebrates, and Viridiplantae) (Fig. 7B). Like our DNA virus results, comparison across methods revealed differences in the overall proportions of identified hosts (Fig. 7C). Tot RNA had the highest proportion of links to Fungi, while PolyA RNA had the highest proportion to Viridiplantae and Invertebrates. Vir RNA had the highest proportion of associations to Bacteria. Together, our RNA vOTU host predictions suggest that methods designed to enrich for specific kingdom-level organisms can also be successful at identifying viral communities that infect different groups, especially for RNA vOTUs.

### Preparation methods show varied trends between viral diversity and transcript abundance by moisture for DNA and RNA vOTUs

**Fig. 8:**
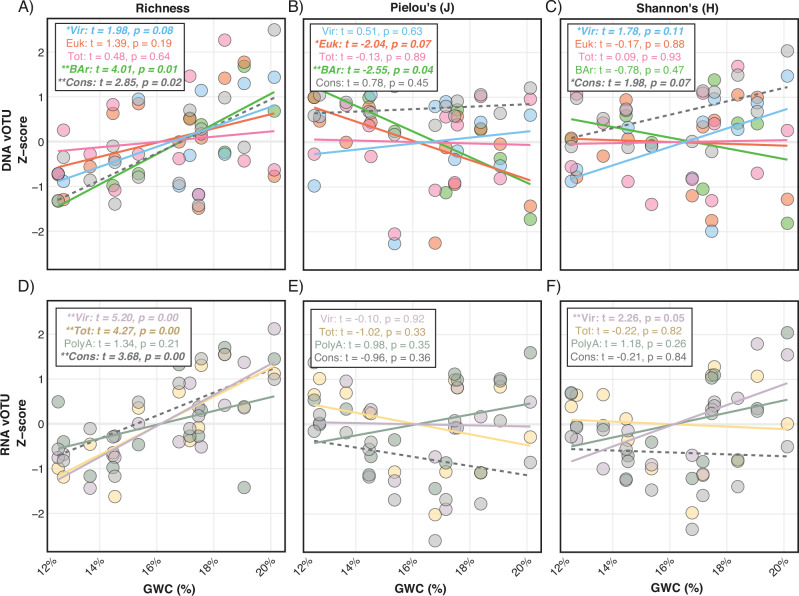
Viral richness, Pielou’s evenness, and Shannon’s diversity across preparation methods and moisture. Top row scatterplots show the measured moisture (x-axis) and the z-score normalized **A** richness (total counts) **B** Pielou’s evenness, or **C** Shannon’s diversity per sample for DNA viral contigs. Bottom row scatterplots show equivalent plots for RNA viruses (**D**, **E**, and **F**). Significance of trends was assessed by fitting separate linear models for each preparation method; reported *p*-values correspond to two-sided tests of the slope term, and no multiple-comparison correction was applied. Significance values are shown in the text boxes and colored by preparation method. Each colored circle represents a preparation method as listed in Fig. S2. The dotted gray line shows the trends for the consolidated database created out of all methods. Non-z-score-normalized diversity values are shown in in Fig. S5.

To assess how each method could influence the interpretation of ecological patterns, we quantified viral diversity for DNA and RNA vOTUs of each individual preparation method (14,121 Vir DNA, 3,757 Euk DNA, 1,017 Tot DNA, 871 BAr DNA, 4,035 Tot RNA, 3892 Vir RNA, 375 PolyA RNA) and compared those to the consolidated databases of each DNA and RNA group (17,590 DNA vOTUs, 6,005 RNA vOTUs), which represent the best available depiction of the soil viral community from our site. We then compared how each method could detect overall trends in relation to the measured moisture conditions (12%-20% gravimetric moisture) of our managed Washington arid grassland (Fig. 8, Figs. S1 and S5). We note that like in Fig. 3, these data were not rarefied, as our intent was to highlight if any given preparation had the capacity to enrich viral particles and potentially generate a more comprehensive viral database which could alter the interpretation of viral diversity.

Richness of both DNA and RNA viruses had marginally significant positive trends in high-moisture samples for Vir DNA, BAr DNA, Vir RNA, Tot RNA, and the consolidated DNA and RNA databases (*p* ≤ 0.1) (Fig. 8A). Contrastingly, Euk DNA, Tot DNA, and PolyA RNA did not detect significant trends by richness and moisture. Pielou’s evenness had varied trends across methods (Fig. 8B), and only Euk DNA and BAr DNA detected marginally significant trends (*p* ≤ 0.1), and both trended negatively with increasing moisture. Using Shannon’s index, only Vir DNA, Vir RNA, and the consolidated DNA database detected marginally significant positive trends with moisture (*p* ≤ 0.1) (Fig. 8C). Together, these results show that not all methods are statistically sufficient to detect moisture trends at the current sampling effort.

**Fig. 9:**
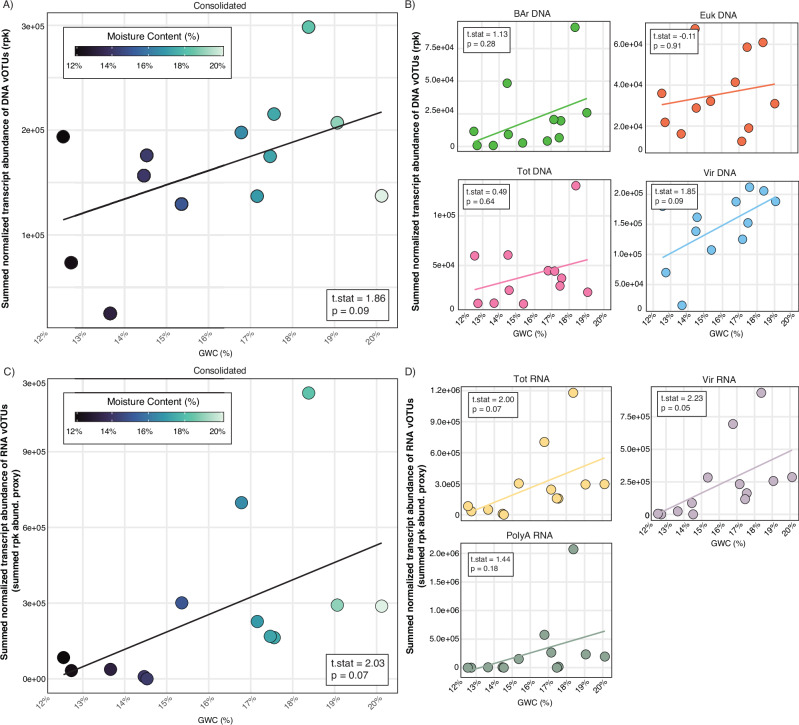
Higher moisture samples have higher activity trends than lower moisture samples, and not all methods significantly capture those trends. Scatterplots denote the relationship between total summed normalized rpk expression and moisture for either DNA or RNA viruses. Linear model statistics are shown in boxes within each plot; reported *p*-values correspond to two-sided tests of the slope term, and no multiple-comparison correction was applied. **A** Expression trends detected across the consolidated database of 17,590 DNA vOTUs. **B** Expression trends detected across the individual viruses that were identified across each preparation method and colored by DNA preparation method (as denoted by colors in Fig. S2). **C** Expression trends detected across the clustered database of 8,302 RNA vOTUs. **D** Expression trends detected across the individual viruses that were identified across each preparation method and colored by RNA preparation method (as denoted by colors in Fig. S2).

Viral transcript abundance for both DNA and RNA vOTUs was calculated by mapping Tot RNA reads to the vOTUs from each individual preparation as well as the consolidated databases of all DNA or RNA vOTUs (see Methods, Fig. 9). For the consolidated databases, vOTUs showed marginally significant (*p* ≤ 0.1) trends of transcript abundance with soil moisture content for both DNA and RNA viral communities (Fig. 9A, C). Individual methods, however, were not equally effective at detecting these trends. For DNA methods (Fig. 9B), Tot DNA, Bar DNA, and Euk DNA vOTUs did not reflect the consolidated database trends. In fact, the only method to detect a significant trend of viral transcript abundance to moisture was the Vir DNA (*p* ≤ 0.1). For RNA methods, Tot RNA and Vir RNA showed marginally increasing transcript abundance trends in relation to increasing soil moisture (*p* ≤ 0.1) (Fig. 9D).

In addition to transcript abundance, we calculated the total number of active vOTUs per sample for the consolidated databases and each preparation method (Fig. S6). Like the transcript abundance results, not all DNA preparation methods were able to match the positive, marginal trend of the consolidated database between active DNA vOTUs and moisture (*p* ≤ 0.1) (Fig. S6A). BAr DNA and Vir DNA detected similar significant (*p *≤ 0.05) positive trends with moisture, while Euk DNA and Tot DNA did not (Fig. S6B). For RNA vOTUs, consolidated databases of active vOTUs (128 vOTUs) did not show trends in relation to moisture (Fig. S5C). While Tot RNA and Vir RNA did not detect any trends, PolyA RNA did, however we note that there were less than 15 active vOTUs detected in the PolyA (Fig. S5D), and warrant caution in the interpretation of viral ecology by PolyA RNA alone^18^.

Together, our results show that not all preparation methods were equally efficient at identifying trends regarding viral transcript abundance or number of active vOTUs by soil moisture in this grassland soil.

## Discussion

### Implementing fractionation methods to enrich distinct subsets of the soil microbiome unearths unique insights into viral communities

Fractionation methods that enrich for specific subsets of the soil microbiome have been around, in some cases, for decades^12^. However, we still lack information on how DNA and RNA fractionations can inform the field of viral ecology. Here, we compared viral communities identified from seven preparation methods to determine to what extent each method characterized the soil virosphere. The debate about whether it is appropriate or not to rarefy sequencing data prior to microbiome analyses is ongoing^30,31^, with both methods being scientifically reasonable. For example, rarefying to a common number of reads is appropriate when the goal is to compare richness across samples at an equalized sampling effort. In our case, however, the purpose of our analyses was to evaluate the overall performance of each preparation method, as implemented in this study, at identifying, assembling, and describing vOTUs and their ecology in a soil environment. Our preparation methods differ significantly in extraction and enrichment strategy, ultimately affecting the resulting proportion of viral reads and sequencing depth. These differences in viral yield and depth are intrinsic aspects of method efficiency. As such, we opted to not rarefy reads across different preparation methods for our comparisons.

We have summarized our initial hypotheses about each preparation method and our observed outcomes in Table 1.

**Table 1:** Comparison of expected versus recovered viral communities resulting from parallel preparation methods intended to enrich distinct subsets of the soil microbiome

Preparation Method	A priori expectations	*A posteriori* results
**Tot DNA** (Total soil DNA)	• Both extracellular and intracellular/cell-associated viruses; inert particles and viruses undergoing infections • Dominated by bacteriophage (Archaea are small portion of soil microbiome) • Putative hosts are predominantly bacteria and archaea (few known fungal/plant DNA viruses)	• Relatively poor recovery and detection of viruses likely due to high community diversity and low read coverage (i.e., challenges to assembly, lower sensitivity for recovering viruses)^15,34^ • Predicted hosts mostly bacterial, with small proportion of archaeal • Large overlap with Vir DNA, but mostly distinct from Euk and BAr DNA.
**Vir DNA** (DNA from viral enrichment)	• Predominantly extracellular viruses; inert particles and recently lytic • Dominated by bacteriophage; depleted of giant viruses (excluded by 0.22um filter)^88^ • Putative hosts are predominantly bacteria and archaea (few known fungal/plant DNA viruses)^89–91^	• Sample processing lyses some cells, releasing intact, intracellular viruses (DNase treatment should digest free DNA) • Highest quantity and quality of DNA vOTUs^15,32^ • Nucleocytoviricota (NCLDV) • Mostly bacterial predictions, but highest proportion of archaeal hosts
**BAr DNA** (DNA from Bacterial/Archaeal cell enrichment)	• Predominantly cell-associated viruses (integrated, intracellular, or attached) • Dominated by bacteriophages (archaea are small portion of microbiome) • Putative hosts are free-living bacteria and archaea	• Method with highest proportion of host predictions • Dominated by bacteriophage (few archaeal viruses, but some detected) • Smallest number of DNA vOTUs detected • Possible that some extracellular viruses get pelleted with cells in density gradients
**Euk DNA** (DNA from hyphal enrichment)	• Predominantly cell-associated viruses (integrated, intracellular, or attached) • Dominated by bacteriophages (few known fungal DNA viruses are ssDNA, and methods did not enrich for ssDNA)^89–91^ • Putative hosts include fungal- or root-associated bacteria	• Fine roots are indistinguishable from hyphae • Potential bias against yeasts (different density than hyphae) • Highest proportion of hosts to *Actinomycetota;* no fungal hosts identified • Large overlap with Vir DNA, but mostly distinct from Tot and BAr DNA.
**Tot RNA** (rRNA-depleted total RNA)	• Predominantly cell-associated (intracellular or attached) viruses (RNA has short half-life) • Composition reflects relative proportion of potential host populations in soil • Putative hosts include bacteria, archaea, fungi, other eukaryotes^92^	• Highest quantity of RNA vOTUs detected • Most unique RNA virus families detected • Lesser quality vOTUs compared to Vir RNA • Highest proportion of fungal hosts, though most assigned hosts were bacterial • Significant overlap with Vir RNA
**Vir RNA** (RNA from viral enrichment)	• Predominantly extracellular viruses; inert particles and recently lytic • Dominated by bacteriophage (mycoviruses primarily replicate through vertical transmission)^23,89–93^ • Putative hosts are predominantly bacteria and archaea (fungal/plant RNA viruses primarily replicate by vertical transmission)	• Less distinct from Tot RNA than expected • Slightly fewer viral families detected than in Tot RNA, but comparable and not as low as PolyA • Highest quality RNA vOTUs recovered • Highest proportion of bacterial hosts • Higher proportion of Viridiplantae and Invertebrate hosts compared to Tot RNA
**PolyA RNA** (polyA-enriched total RNA)	• Predominantly cell-associated (intracellular or attached) viruses • Dominated by mycoviruses (assuming viruses preferentially modify RNA to align with host)^46^ • Putative hosts are eukaryotes (fungi, plants, invertebrates in soil)^18^	• Method had highest enrichment of predicted eukaryote hosts (mostly plants and invertebrates) • Smallest number of RNA vOTUs detected^18^ • Smallest proportion of fungal hosts • Some bacterial hosts as bacteriophage can polyadenylate their genomes as host defense mechanisms

Preparation methods were designed to enrich extracellular (free) virus particles (Vir), bacterial and archaeal cells (BAr), fungal hyphae and fine roots (Euk), or unfractionated total soil (Tot). Fractionation methods were followed by metagenome or metatranscriptome (with/without polyA-selection or rRNA depletion) sequencing to characterize DNA and RNA viral sub-communities, respectively.

### Choice of DNA preparation method can change interpretation of viral ecology

DNA extracted from purified viral particles (i.e., viromes, Vir DNA) outperformed all other DNA methods in both quantity and quality of recovered vOTUs (Fig. 2, Figs. S2 and S3). Across parallel extractions, individual DNA preparation methods identified 19,769 vOTUs, where 14,124 vOTUs (81%) were recovered from DNA viromes (Vir DNA). Our results reinforce previously reported observations that Vir DNA is consistently more effective than total metagenomes (Tot DNA) at characterizing viral communities in soils^15^ and other ecosystems^32,33^. Consistent with those reports^15,33^, we observed pronounced shifts in the taxonomic proportions of computationally predicted hosts between Vir DNA and Tot DNA (Fig. 6), with Vir DNA identifying the highest proportion of archaeal genomes. Moreover, relationships between moisture and either vOTU diversity (richness, Pielou’s, Shannon’s) (Fig. 8) or expression (Fig. 9) can differ between methods. We hypothesize these differences could 1) result from selection of communities specific to different ecological niches targeted by fractionation method, and/or 2) stem from the combination of high viral and organismal diversity present in soils which can hinder sufficient data collection for viral recovery^15,34^.

Vir DNA uniquely bridged the viral communities recovered by Tot DNA and eukaryote-enriched DNA (Euk DNA), whereas Tot DNA and Euk DNA showed virtually no detection or recovery overlap with each other (Fig. 2A, C). These patterns suggest that Euk DNA captures not only hyphae and root-associated viral communities, but also viruses associated with select free-living cells and some extracellular viruses. For example, Euk DNA had the highest proportion of predicted *Actinomycetota* hosts, which are known to generate branched filaments^35^, and thus might be enriched by the flotation-based enrichment approach compared to other free-living bacterial cell morphologies. We hypothesize that these viruses are likely not detectable at the standard sequencing depth collected for Tot DNA, resulting in non-overlapping communities. We also hypothesize that the overlap in vOTUs between Euk DNA and Vir DNA results from extracellular viruses adsorbing to the high surface area represented by hyphae and fine roots during sample preparation. Paired extractions of model communities with mixed cell morphologies in simulated soil conditions would help resolve possible sources of variation among methods.

The bacterial/archaeal-enriched fractionation (BAr DNA) stood out as the method with least overlap with the others. This distinct viral community likely reflects selective enrichment of intracellular (lysogenic temperate viruses, which may or may not be integrated) or tightly host-associated viruses that co-fractionate with intact cells. In rhizosphere systems, induction of integrated viruses changes viral and bacterial communities, subsequently leading to downstream effects on ecosystem nutrient cycling^36^. The clear separation between BAr DNA-associated and Vir DNA-associated viral communities could be explained by the viral seed bank hypothesis^37^, in which cell-associated and free (i.e., extracellular) integrated virus populations represent largely distinct pools. Consequently, our results suggest that BAr fractionations may be useful for studies aimed at contrasting temperate versus virulent infection cycles or quantifying the soil prophage reservoir.

Ultimately, our results support and extend what other groups have advocated for in sequence-based studies of the soil virosphere^32,33^: it is important to consider the scientific question that is going to be addressed prior to choosing a DNA preparation method, as it can alter the interpretation of soil viral ecology.

### RNA preparation methods capture overlapping RNA viral communities, yet still provide distinct insights

While RNA viromes have been used to assess the composition and ecology of soil RNA viruses^38^, prior to this study the recovery efficiency of RNA vOTUs had not been directly compared between total soil metatranscriptomes (Tot RNA) and purified viral RNA (i.e., RNA viromes, Vir RNA). Contrary to DNA vOTU results, the number of ssRNA vOTUs recovered and detected was almost equal between Vir RNA and Tot RNA (Fig. 3A), with Tot RNA recovering only 143 more vOTUs than Vir RNA, and the two methods sharing almost complete overlap by the read-mapping detection method (Fig. 3C). However, Vir RNA produced the longest and highest quality vOTUs among RNA methods for 69% of viruses recovered (Fig. S3).

In addition to the quality distinctions, the overall proportion of predicted host assignments differed between Tot RNA and Vir RNA vOTUs. For example, the highest proportion of predicted fungal hosts came from Tot RNA (Fig. 7C), consistent with the understanding that fungal RNA viruses are obligately intracellular^39^, which likely explains why they are underrepresented in extracellular Vir RNA preparations. Further, eukaryote organisms tend to polyadenylate their mRNA for stability, nuclear export, and translation^17^. PolyA RNA (polyA enrichment of total soil metatranscriptomes) had the highest proportions of eukaryote linkages within *Viridiplantae*, Invertebrates, and Chordata (Fig. 7). Despite fungi also polyadenylating their mRNA, the low total yield of PolyA RNA vOTUs and inefficiencies in PolyA selection^18^, alongside the dominance of plant-associated viruses, likely limited detection of fungal viruses in this fraction, as nucleic acid extractions from fungi are known to be challenging^40,41^. vOTUs from polyA RNA belonged to orders recognized for polyadenylation: *Picornavirales*^42^, *Patatavirales*^43^, *Stellavirales*^44^, and *Tymovirales*^45^, though the purpose of polyadenylation in viruses remains enigmatic^46^.

One practical limitation for the implemented Vir RNA protocol^38^ was that our Vir RNA did not undergo a standard ribosomal RNA (rRNA) depletion and thus was overwhelmingly composed of ribosomal reads (89% on average). We concur with Hillary et al.^38^ and recommend rRNA depletion prior to RNA virome sequencing. However, we recognize that this process is not commonplace because RNA yields from viral pellets are low, demanding very large soil samples (kilograms of soil), which can aggregate ecologically distinct viral communities, in turn challenging detection of relevant ecological trends. Another consideration for method selection is that RNA viruses can have higher mutation rates than DNA viruses^47^, making viral genome quality critical for addressing processes like genomic adaptations. To this point, only 22% of RNA vOTUs were shared between Tot and Vir RNA, denoting a significant degree of non-overlap, possibly due to high RNA virus diversity.

Given these insights, we recommend careful consideration of potential tradeoffs between genomes of higher viral quality (Vir RNA) and genomes with additional ecological context enabled by quantification of transcript abundance patterns of both viral and host communities (Tot RNA), or specific members of the soil microbiome like eukaryotes (PolyA RNA). Each method offers a distinct advantage, and it is likely that their complementary use, rather than any single approach, will best advance our understanding of soil RNA viral ecology and interkingdom interactions.

### Virus profiles from managed grassland provide insights into viral ecology

Soil moisture regulates the hydraulic connectivity among soil pores, which controls microbial substrate access and community interactions^48^. Soils pore spaces may be filled by liquids, gases, or a mixture of the two^3^, while solids make up only about half of the volume of any given soil. Lower moisture levels in historically moist systems can result in low pore connectivity, favoring higher overall diversity of microbial communities aggregated across microsites^49^. Conversely, lower moisture (i.e., increased aridity) in arid systems is associated with reduced soil microbial diversity and abundance^50^. While much less studied, these variable patterns are also observed for soil virus communities, and are very likely due, at least in part, to their dependency with their respective hosts. DNA viral richness can both increase^28^ and decrease^25^ with lower moisture soils. Similarly, DNA virus expression and total number of active viruses, as well as RNA vOTU abundance, can increase or decrease with higher moisture soils^24^. These conflicting responses support previous findings that soil moisture effects on microbial ecology are system-specific and depend on intrinsic soil characteristics (i.e., soil texture, porosity) that influence soil matric potential and hydraulic connectivity^51,52^.

The arid grassland soil at our experimental site has been extensively characterized^53,54^ (see methods). We hypothesize that viral activity and diversity in this system increase with moisture up to an optimal saturation point of gravimetric moisture. At this optimal point, host resource acquisition (i.e., C availability), viral dispersion, and ecological niche differentiation are balanced, leading to increased viral diversity and activity. Beyond that optimal point, we hypothesize that viral communities, much like their microbial counterparts, become homogenized, leading to decreased diversity and number of active vOTUs but increased overall viral expression. The optimum is dependent on intrinsic soil properties like porosity, density and permeability, so, naturally, different soil types will display different optimal moistures. This is highly speculative, and extensive targeted research across a broad range of soil types is needed to confirm these hypotheses. We urge the incorporation of measurements like effective water saturation (i.e., volume ratio of water filled soil pores) and intrinsic soil properties like percent sand/silt/clay, bulk density, percent organic matter, and cation exchange capacity^3,55^ to contextualize results with the hope that contrasting trends among sites can be reconciled, ultimately leading to a generalized understanding of viral response to soil moisture gradients.

Together, this study characterizes the different depictions of a viral community resulting from parallel DNA and RNA preparation methods and uncovers the complexity of DNA and RNA viral communities in an arid grassland soil environment. While more research is needed to compare the results here with different soils from other systems, different preparation methods unearthed distinct DNA and RNA viral communities associated with different host groups. We also present the first comparison between Vir RNA (i.e., RNA viromes) and Tot RNA (i.e., soil metatranscriptomes) with regards to RNA virus recovery and ecological interpretation. In agreement with previous publications, Vir DNA significantly outperforms all other methods in DNA viral detection and recovery. However, we show that virus host identifications for DNA vOTUs were different between methods. While both Vir RNA and Tot RNA methods identified a similar number of viral contigs, there were tradeoffs regarding quality and length (Vir RNA outperforms), and differences in the overall proportions of host assignments (Tot RNA assigned more fungi, Vir RNA assigned more bacteria). Method choice therefore may impact interpretation of DNA and RNA virus ecology through fractionated sampling of the broader viral community. Notably, Tot DNA alone did not detect any significant trends by richness or expression in relation to moisture, in contrast to the positive trends identified by Vir DNA. Each RNA preparation method evaluated, however, found a positive relationship between RNA viral richness and expression and soil moisture. While our work only focuses on the soil of a single arid grassland field site and a narrow moisture gradient (12-20% gravimetric moisture), our findings underscore the importance of tailored, soil-specific evaluations when studying viral ecology, encouraging researchers to consider ecological and methodological contexts when developing and addressing specific hypotheses.

## Methods

### Field Site management

Samples were collected from the Tall Wheatgrass Irrigation Field Trial in Prosser, WA, USA (46°15′04″N and 119°43′43″W), operated by Washington State University, and described in our previous publications^56–58^. The site is characterized by marginal Aridisol soils with low organic matter content ( < 2%), pH of 8, and a sandy loam texture (55.5% sand, 34.1% silt, 10.4% clay). Specifically, it is described as coarse-silty, mixed, superactive, mesic Xeric Haplocambids and having high porosity, permeability, and soil bulk density (avg = 1.56 g/cm^3^)^53^. Tall wheatgrass (*Thinopyrum ponticum*), which is drought tolerant and adapted for growth on marginal soil, was established in May 2018, prior to which the site was uncultivated desert shrub-steppe. Plants are uniformly distributed within plots. Irrigation treatments have been ongoing since spring 2019. Irrigation is provided through drip lines from April to October with water supplied at four levels (100%, 75%, 50%, and 25% field water capacity) to create plots with differing water stress based on modeled crop evapotranspiration of tall wheatgrass^59,60^. Each experimental plot is 2.1 m × 10.7 m with a 1.5 m alley between adjacent plots (Fig. S1).

### Sample collection and processing

Soil cores were collected on 18 Oct 2022 and 7 March 2023 from plots within the highest (100%) and lowest (25%) irrigation treatments, including 3 field replicates per treatment. All sampled plots were planted with the Alkar cultivar, except one plot with the Jose cultivar (Plot 40, 100% irrigation treatment) which was re-sampled in the spring for consistency, resulting in unequal sample numbers between collection efforts (*n* = 6 for October 2022, *n* = 7 for March 2023). Within each plot, one core (5 cm diameter) was collected from a random location down to 15 cm depth. The 0–5 cm portion was discarded to remove the surface litter layer. The 5–15 cm portion of each soil core was aseptically broken up and a subsample for Tot RNA was snap frozen in liquid nitrogen (and stored at −80 °C prior to RNA extraction). The remaining soil was transported from the field site to the Pacific Northwest National Laboratory (PNNL) on ice for processing.

In the laboratory, 2 mm sieves were used to homogenize soil and remove large roots and rocks prior to subsampling. All subsampling was completed the same day as sample collection. Subsamples for bacterial and archaeal (BAr), fungal (Euk), and viral (Vir) fractionation were stored at 4 °C until further processing. Soil for DNA extraction were stored at −80 °C until processing. The soil water content was measured by the gravimetric method^61^ for each sample. Briefly, 10 g of soil was dried at 60 °C until a stable weight was achieved^62^. Gravimetric water content (GWC) was calculated as the fresh soil weight minus the dry soil weight, relative to fresh soil weight. pH was determined in 1:2 soil water slurries according to^63^. Sample metadata is available in Data S1.

### Sample fractionation for bacteria/archaea, fungal hyphae, and viruses

Bacterial and archaeal cells were separated from total soil using a Nycodenz density gradient^64^. 15 mL of 1X PBS with 0.01% Tween20 was added to 10 g soil and vortexed for 15 min. After allowing the soil slurry to settle for 5 min, supernatant was transferred to a new tube and diluted with 4 mL of the PBS/Tween solution. The slurry was vortexed again and allowed to settle before the supernatants were pooled. Two layers of Nycodenz were added below the supernatant: 5 mL of 40% Nycodenz then 2 mL of 80% Nycodenz below that. Gradients were centrifuged at 5,000 x g for 15 min at 4 °C with a slow ramp and slow brake. The top two layers (aqueous phage and 40% Nycodenz) were transferred to separate tubes and diluted with an equal volume of 1X PBS. Cells were pelleted by centrifuging at 7,000 x *g* for 15 min, resuspended in 0.1 mL 1X PBS, and combined. Extracted cells were stored at −20 °C until DNA extraction.

Fungal hyphae (and fine roots) were separated from total soil using a modified hyphal float approach^65^. 30 mL of 4 M KCl was added to 10 g soil and gently inverted to mix for 5 min. Slurries were allowed to settle for 1 min to sediment soil before decanting the supernatant with hyphae to a clean tube. Soil was re-extracted once with 4 M KCl and twice with 2 mL sterile DI water. Hyphae were collected by filtering pooled supernatants through 40 μm mesh and transferred to microcentrifuge tubes with sterile tweezers. Extracted hyphae were stored at −80 °C until DNA extraction.

Extracellular DNA and RNA viruses were separated from total soil following the Soil Viromics Protocol from the Emerson lab group (https://www.protocols.io/view/soil-viromics-protocol-emerson-lab-v1-kxygxz7q4v8j/v1) with modifications inspired by Hillary et al.^38^. For each sample, 120 g of soil was evenly distributed (20 g each) into 50 mL tubes and extracted with 18 mL PPBS buffer (2% BSA, 10% PBS, 1% K-citrate, 150 mM MgSO4). Slurries were shaken at 300 rpm for 10 min at 4 °C and centrifuged at 4000 x  *g* for 10 min at 4 °C in swinging bucket rotor. Supernatant was decanted to a clean tube and pooled with the supernatants of two subsequent extractions. Supernatant was decanted to a clean tube, and the extraction steps were repeated two additional times for each soil sample. Supernatants were pooled resulting a final volume of approximately 54 mL per sample. Pooled supernatants were centrifuged at 10,000 x *g* for 8 min at 4 °C in a fixed angle rotor and filtered through 0.22 µm PES (Thermo Scientific) to remove microbial cells. To pellet viruses, cell-free supernatants were ultracentrifuged at 35,000 x *g* for 3 h at 4 °C under vacuum. Supernatant was decanted, taking care not to disturb the viral pellet. Each pellet was resuspended in 100 µL cell culture grade water and DNase-treated following manufacturer recommendations (Promega RQ1 RNase-Free Dnase). Resuspended and pooled viral pellets were stored at −80 °C until further extraction.

### Nucleic acid extraction (DNA and RNA)

DNA was extracted from bacterial/archaeal cells (BAr), and fungal hyphae (Euk), and soil (Tot-DNA) using the Quick-DNA Fecal/Soil Microbe Miniprep Kit (Zymo) according to manufacturer instructions. DNA yield was quantified by Qubit DNA High Sensitivity assay (Invitrogen) and purity was checked by NanoDrop spectrophotometry. Extracted DNA was stored at −80 °C prior to being shipped for sequencing. Tot RNA (2 g) was extracted with the RNeasy PowerSoil Total RNA kit (Qiagen) according to manufacturer instructions. The resulting RNA was DNase treated with the TURBO DNA-free™ Kit (Invitrogen) according to manufacturer instructions. Extracted RNA was quantified using the Qubit RNA High Sensitivity assay (Invitrogen), purity was checked by NanoDrop spectrophotometry, and quality was assessed by RNA 6000 Nano Kits (Agilent). Samples were stored at −80 °C prior to being shipped for sequencing. RNA and DNA were co-isolated from resuspended viral pellets using the RNeasy PowerSoil Total RNA Kit (Qiagen) in combination with the RNeasy PowerSoil DNA Elution Kit (Qiagen) and quantified as described above. Extracted nucleic acids were shipped to the JGI for metagenome and metatranscriptome library prep and sequencing, in support of user proposal 509015. Sample metadata can be found in Data S1.

### Sequencing and library preparation methods (DNA and RNA)

DNA sequencing data were generated at the DOE Joint Genome Institute (JGI) using Illumina technology. For Virome DNA, an Illumina Low Input (DNA) library was constructed and sequenced using the Illumina NovaSeq X platform. For Tot, Euk and BAr DNA, an Illumina regular (DNA) library was constructed using the Illumina NovaSeq X platform. JGI’s standard computational pipeline was used to process the reads. Briefly, BBDuk (version 39.01)^66^ was used to remove contaminants, trim reads that contained adapter sequence and homopolymers of Gs of size 5 or more at the ends of the reads, remove optical duplicates from data generated on the NovaSeq X platform, and right quality trim reads where quality drops to 0. BBDuk was used to remove reads that contained 4 or more ‘N’ bases, had an average quality score across the read less than 3 or had a minimum length ≤51 bp or 33% of the full read length. Reads containing Swift sequences were trimmed from the left or right of the read. Reads mapped with BBMap to masked human, cat, dog and mouse references at 93% identity were removed. Reads aligned to common microbial contaminants were removed.

RNA samples extracted from total soil were split and treated with poly-A selection to enrich for eukaryote RNA (PolyA RNA) or rRNA depleted (Tot RNA) with Qiagen FastSelect kit with pooled bacterial, yeast, and plant probes to enrich for bacterial/archaeal mRNA prior to sequencing. RNA sequencing data were generated at the DOE Joint Genome Institute (JGI) using Illumina technology. For Virome RNA, an Illumina Ultra-Low Input (RNA) library was constructed and sequenced using an Illumina NovaSeq X 270 bp fragment platform and a TruSeq RNA library preparation kit. For PolyA and Tot RNA, an Illumina low input (RNA) library was constructed and sequenced using an Illumina NovaSeq X 270 bp fragment platform and a TruSeq RNA library preparation kit. BBDuk (version 39.01)^66^ was used to remove contaminants, trim reads that contained adapter sequence and homopolymers of G’s of size 5 or more at the ends of the reads, remove optical duplicates from data generated on the NovaSeq X platform, and right quality trim reads where quality drops to 0. BBDuk was used to remove reads that contained 1 or more ‘N’ bases, had an average quality score across the read less than 10 or had a minimum length ≤51 bp or 33% of the full read length. Reads mapped with BBMap to masked human, cat, dog and mouse references at 93% identity were removed as well as reads aligned to common microbial contaminants, ribosomal RNA reads, or known spike-ins. All sample metadata can be found in Data S1.

### DNA and RNA viral contig identification

After DNA sequencing at the Joint Genome Institute (JGI) from all different data types, the resulting reads were quality-filtered with the default JGI workflow that uses bbduk with flags ktrim=r, ordered, minlen=51, minlenfraction=0.33, mink=11, tbo, tpe, rcomp=f, k = 23, hdist=1, hdist2 = 1, ftm=5, pratio=G,C, plen=20, phist, qhist, bhist, and gchist. These reads were downloaded from the JGI data portal and assembled in-house using MEGAHIT^67^ with default settings. To identify viral contigs from our assemblies, we used the modular viromics pipeline (MVP) v1.1.1^68^, which uses geNOMAD v1.7.4^69^, CheckV v1.0.3^70^, trimal v1.5.0^71^, mafft v7.526^72^, and FastTree v2.1.1^73^. MVP was run with a minimum scaffold cutoff of 10 kb. Viruses identified in each sample were then clustered at 95% ANI across 85% of the shortest contig per MiUViG standards^74^. DNA vOTUs were required to have a viral score of ≥0.7, and no more host genes than viral genes, yielding a per-method clustered database of 19,759 DNA vOTUs that met our quality cutoffs, which were then clustered across all sample types generating 17,590 DNA vOTUs. All viral information can be found in Data S2.

After RNA sequencing at JGI from all different data types, reads were quality-filtered and assembled with the standard JGI workflow which used MEGAHIT^67^ with default settings and flag –k-list 23, 43, 63, 83, 103, 123. Assemblies were downloaded from the JGI data portal, and MVP was run. Given that RNA viruses are often segmented and there is no size consensus for a minimum size, MVP was run with the same settings as above, with the exception that we did not require a minimum size cutoff. RNA viruses were required to have a virus score ≥0.7, no more host genes than viral genes, and required to have an RNA-dependent RNA polymerase (RdRp) gene. The identification of RdRps were taken from MVP which uses HMMER^75^, a default score of 50, and a minimum e-value of 0.01 to search the geNOMAD RdRp database.

RNA viruses that had an unknown genome type (RNA) or ssRNA genome type and had an RdRp were subject to phylogenetic trees to confirm their taxonomy with the Riboviria database^23^ (see section on RNA virus taxonomic assignment). With the confirmed taxonomic assignment, their genome types were corroborated using the latest International Committee on Taxonomy of Viruses (ICTV) master list^76^. Only confirmed ssRNA viruses that had positive or negative genome types with no instance of an ambisense genome by taxonomy were retained for downstream analyses. This yielded a database of 8302 vOTUs clustered per-method that was used to address the differences in efficiency of RNA methods. Given that using current methods it is impossible to assign transcript abundance information to double stranded RNA (dsRNA) viruses, and to keep the analyses between the ecological and methodological aspects of this manuscript comparable, we did not account for RNA viruses that had dsRNA (515 dsRNA vOTUs). Additionally, viruses that could not confidently be assigned to a genome type were also removed (323 vOTUs). Finally, viral contigs that were opposite to the data type from which they came from were removed (i.e., RNA viruses from DNA methods, or DNA viruses from RNA methods, 8 vOTUs).

### DNA virus taxonomic assignment

Viral taxonomy for DNA viruses was assessed using vContact2 v0.11.3^77^. Viruses were compared to the reference database of RefSeq v211. Our vOTUs were run through Prodigal v2.6.3 using default settings to identify protein coding genes, resulting in a database of 761,653 genes. Then, vContact2 was run with flags --rel-mode Diamond --db “ProkaryoticViralRefSeq211-Merged” --pcs-mode MCL --vcs-mode ClusterONE --pc-evalue 0.0001 --reported-alignments 25 --max-overlap 0.8 --penalty 2.0 --haircut 0.1 --pc-inflation 2.0 --vc-inflation 2.0 --min-density 0.3 --min-size 2 --vc-overlap 0.9 --vc-penalty 2 --vc-haircut 0.55 --merge-method single --similarity match --seed-method nodes --sig 1.0 --max-sig 300 --mod-inflation 5 --mod-sig 1.0 --mod-shared-min 3 --link-sig 1.0 --link-prop 0.5 --verbose -vv -o./ -t 32 --c1-bin. vContact2 taxonomic assignments are found in Data S3.

### RNA virus taxonomic assignment

RNA virus contigs that had an RdRp sequence were assigned taxonomy via phylogenetic trees. To build a reference of RdRps for alignment while also reducing compute times, preliminary taxonomic assignment by MVP was used to download a set of reference sequences from the Riboviria database^23^ that matched the MVP-assigned taxonomies. All viral contigs that corresponded to each taxonomic assignment were downloaded. For specifics of which viral contig was placed with which tree, see Data S2. After all corresponding sequences for each phylogenetic group were downloaded, we did a blastp search using the RdRp from our viral contigs as the query, and the entirety of corresponding Riboviria group as the reference and retained the best hit. RdRp sequences from each group were then aligned using MUSCLE5^78^ to the references, resulting in a total of 21 unique alignments. The resulting alignments were then automatically trimmed using trimAl^71^ using flag -automated1 and subsequently used to generate a phylogenetic tree using FastTree^73^. Trees were then re-rooted to the specified outgroup (Data S2) using ETE3^79^. ssRNA viral contigs from our dataset were assigned the taxonomic string of the nearest neighbor from the Riboviria reference. RdRp results are found in Data S2.

### DNA and RNA virus host predictions

Host assignment for DNA vOTUs were performed using iPHoP version 1.4 run with default settings with the Jun25_rw database^80^. iPHoP results were then processed using a custom parser that is available on GitHub that only reports the hit(s) that had the highest confidence score per each virus and ensures a minimum confidence score of 90. Host assignment for RNA vOTUs were performed using RNAVirHost^29^ and using the taxonomic assignments that were generated with this manuscript. Hits were only considered if they were determined by RNAVirHost to be “high confidence” or “assigned”.

### Viral recovery and DNA/RNA read mapping for abundance and transcript abundance

The vOTUs from each preparation were clustered across all methods at 95% ANI across 85% of the shortest contig, which generated the 17,590 and 6005 DNA and RNA vOTU final tables, respectively. To determine whether a virus was recovered (i.e., fully assembled and classified as viral) from each method, the viral clustering output was parsed using a parser available on GitHub (https://github.com/jrr-microbio/interkingdom_virus)^81^. If a viral contig was clustered across different methods, it was assigned an overlap count (Figs. 2A and 3A). To identify whether viruses were detected in each preparation method at a read level (e.g., sequencing level), individual reads from each preparation were mapped to the consolidated databases of either DNA (17,590 vOTUs) and RNA vOTUs (6,005 vOTUs) (Figs. 2B, C and 3B, C).

For DNA vOTUs, individual preparation reads were mapped using bbmap and filtered to a 98% minimum percent of identity using reformat.sh. After, resulting SAM files were processed through CoverM version 0.7.0^82^ to apply a 75% minimum coverage and 1x depth cutoff for each hit. A virus was considered detected if it had assigned counts at the specified cutoffs. To compare DNA virus diversity trends, reads from each preparation were mapped to a database of vOTUs from each respective preparation. Additionally, Tot DNA reads were mapped to the consolidated database of all 17,590 vOTUs for the “Full” viral diversity that is shown in Fig. 8. All mapping is done as specified above using bbmap. To determine DNA virus transcript abundance, total RNA reads were mapped to the consolidated database of 17,590 vOTUs as well as individual vOTU databases from each method by using bbmap and filtered to a 98% minimum percent of identity using reformat.sh (Fig. 9). Then, featureCounts^83^ was run to estimate read counts per gene region. The resulting transcript abundance values were imported into R and normalized by using a gene length corrected trimmed mean of M-values normalization (geTMM)^84^.

For RNA vOTUs, a TruSeq kit was used which enabled strand-conserved sequencing. Both the strandedness of genes within an RNA virus and their taxonomic assignment are critical for assigning reads as being representative of either abundance or transcript abundance. Further, because taxonomic assignment in RNA viruses depends on the highly conserved RdRp gene, any viruses that did not contain an RdRp with a minimum e-value of 0.1 and a match score of 50 from the MVP RdRp annotation pipeline was subsequently removed from further analyses, and the RdRp of each of those RNA viruses was used as the “genomic representative” for each genome’s ecological patterns. While we recognize that dsRNA viruses are important, only viruses that were ssRNA viruses were used to assign abundance and transcript abundance patterns given that their replication process involves the formation of a complementary strand.

Leveraging the gene and strandedness assignments from geNOMAD as part of the MVP pipeline, we utilized the MVP “geaParser” to assign read counts to either the coding strand or non-coding strand^85^ which reads each BAM file and takes into account whether reads were mapped from either template or non-template strand of our RdRp sequences. A post-processing script then considered the strandedness of the viral gene to determine if mapped reads represented abundance or transcript abundance. All code is available on GitHub^81^. ssRNA viruses were considered abundant if recruitment happened in the template strand, and the overall “abundance” was inferred as the total count of reads identified per sample. ssRNA viruses were considered active if recruitment happened on the non-template strand. Given that the significance of higher or lower counts in RNA virus transcript abundance is still debated, RNA viruses that were detected as active were then assigned their respective abundance value for each sample as a proxy for their transcript abundance as has been done previously^86^. RNA viruses that were identified as potentially active but not abundant were removed from subsequent analyses (113 vOTUs) as they likely represented viruses that did not have reads recruiting to the expected strands due to the mapping cutoffs used.

Moisture was measured as described above and used as a continuous variable for statistics to relate the viral diversity and expression metrics detailed above. A lm function was run using R base stats and *t* test values and *p*-values for each are reported in each figure.

### Reporting summary

Further information on research design is available in the Nature Portfolio Reporting Summary linked to this article.

## Supplementary information

Supplementary Information

Description of Additional Supplementary Files

Supplementary Dataset 1

Supplementary Dataset 2

Supplementary Dataset 3

Reporting Summary

Transparent Peer Review file

## Data Availability

Metadata, viral genomes, microbial and archaeal genomes, as well as sample metadata are publicly available at PNNL DataHub^87^. Raw reads for all data are available on the JGI data portal (https://data.jgi.doe.gov/) under project IDs: PolyA RNA: 1440986-1440991 and 1441011-1441017, Tot RNA: 1440986-1440991 and 1441011-1441017, Euk DNA: 1441083-1441088 and 1441096-1441102, BAr DNA: 1441077, 1441079, 1441081-1441082, 1441091, and 1441093-1441095, Vir DNA: 1441129-1441141, Vir RNA: 1440998-1441010. Data is also directly linked by NCBI project IDs: PolyA RNA (PRJNA1412023, PRJNA1466428, PRJNA1412024, PRJNA1409590, PRJNA1410383, PRJNA1408336, PRJNA1410213, PRJNA1409451, PRJNA1408337, PRJNA1408032, PRJNA1408683, PRJNA1412026, PRJNA1409285), Tot RNA (PRJNA1412023, PRJNA1466428, PRJNA1412024, PRJNA1409590, PRJNA1410383, PRJNA1408336, PRJNA1410213, PRJNA1409451, PRJNA1408337, PRJNA1408032, PRJNA1408683, PRJNA1412026, PRJNA1409285), Euk DNA (PRJNA1408512, PRJNA1408591, PRJNA1408772, PRJNA1411830, PRJNA1412022, PRJNA1409283, PRJNA1410547, PRJNA1408334, PRJNA1408944, PRJNA1408335, PRJNA1411524, PRJNA1411726, PRJNA1411725), BAr DNA (PRJNA1408593, PRJNA1408948, PRJNA1408207, PRJNA1408423, PRJNA1408597, PRJNA1410546, PRJNA1410004, PRJNA1408425), Vir DNA (PRJNA1409100, PRJNA1408339, PRJNA1411076, PRJNA1410354, PRJNA1408685, PRJNA1410694, PRJNA1410197, PRJNA1408203, PRJNA1409665, PRJNA1409846, PRJNA1408598, PRJNA1410331, PRJNA1411077), Vir RNA (PRJNA1409829, PRJNA1408774, PRJNA1412028, PRJNA1411922, PRJNA1409847, PRJNA1410505, PRJNA1411837, PRJNA1409102, PRJNA1411375, PRJNA1410666, PRJNA1409452, PRJNA1409193, PRJNA1412132). See supplemental file Data S1 for more information on each sample. All trimmed reads are publicly available on the JGI Data Portal and the PNNL DataHub (https://data.pnl.gov/group/7/nodes/dataset/37100). Protein fasta files used for phylogenetic analysis as well as all supplemental code are available on GitHub (https://github.com/jrr-microbio/interkingdom_virus)^81^. All data that is needed to generate the figures is present in Supplemental Tables, and all code is available to process and reproduce those within the GitHub location.

## Code availability

Full code and files for all analyses are available on GitHub (https://github.com/jrr-microbio/interkingdom_virus)^81^. Code for phylogenetic analyses is available on GitHub (https://github.com/jrr-microbio/interkingdom_virus)^81^. The geaParser python script made by the MVP authors^68^ is provided on GitHub as a standalone script (https://github.com/jrr-microbio/interkingdom_virus)^81^. The custom parser used for DNA virus taxonomic assignments is available on GitHub (https://github.com/jrr-microbio/interkingdom_virus)^81^.
