# Supplementary material for: Preparation method shapes the recovery and ecological interpretation of DNA and RNA soil viral communities
Source: Nat Commun. 2026 Jul 28;17:7551. doi: 10.1038/s41467-026-74154-1 (PMC13415888; doi:10.1038/s41467-026-74154-1)
Supplement: Supplementary file 1 — Supplementary Information [file 41467_2026_74154_MOESM1_ESM.pdf]

Supplementary Tables:

Table S1: Sample Metadata Sheet: Metadata for each site, Metadata for each sample, Stats for read mapping results, Project IDs and sample metadata from JGI for data accessibility, and Collected biogeochemical measurements from each sample.

Table S2: vOTU Tracking Sheet: Viral information including All vOTUs from DNA Virome, All vOTUs from RNA Virome, All vOTUs from DNA Bacterial, All vOTUs from DNA EukFloat, All vOTUs from RNA Bulk, All vOTUs from RNA PolyA, Clustering dataframe of vOTUs from each method after being clustered across all methods to detect the vOTU overlap across methods, Taxonomic assignments via phylogenetic trees for the 8,335 RdRps encoded in 8,302 RNA vOTUs from all data methods, Masterlist downloaded from ICTV on October 20, 2024. Specifically, file version: ICTV_Master_Species_List_2023_MSL39.v3.xlsx (https://ictv.global/msl), Results from virus host matching of DNA viruses, and Results from virus host matching of RNA viruses

Table S3: vContact2 Results: Sheet containing the vContact2 output for all DNA vOTU taxonomic groupings.

Table S4: MAG data including Taxonomic identity of each MAG identified in bacterial DNA and Quality information of each MAG identified in bacterial DNA

Supplementary Figures:

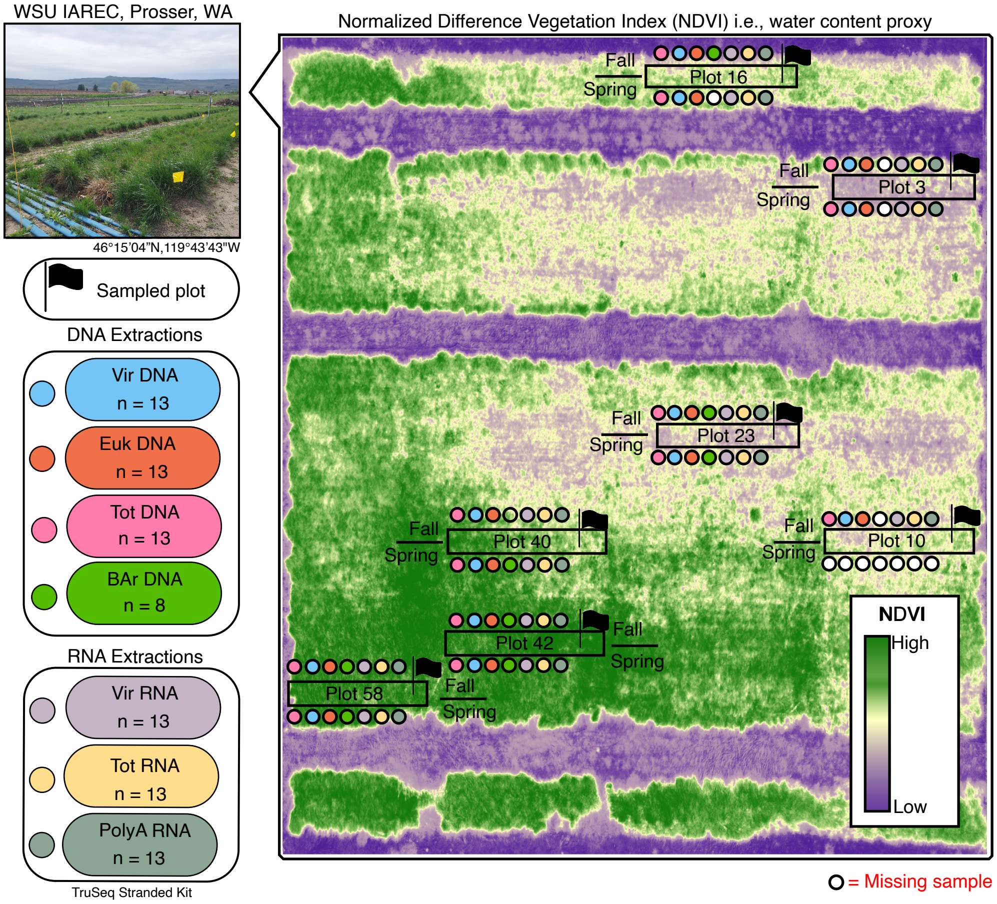

Supplementary Figure 1: Site-level overview of collected samples. Drone image showing the field plot where samples were collected. Field color scale denotes the normalized difference vegetation index (NDVI) which is used as a proxy for water content. Plot IDs with flags and squares denote areas that were sampled, and colored circles represent which data types were collected from each site. Circles above the plot denote fall sampling, while circles below the box denote spring sampling. Empty circles denote missing samples. NDVI image from September 6, 2022, from the Washington State University Unmanned Aerial Vehicle multispectral sensor.

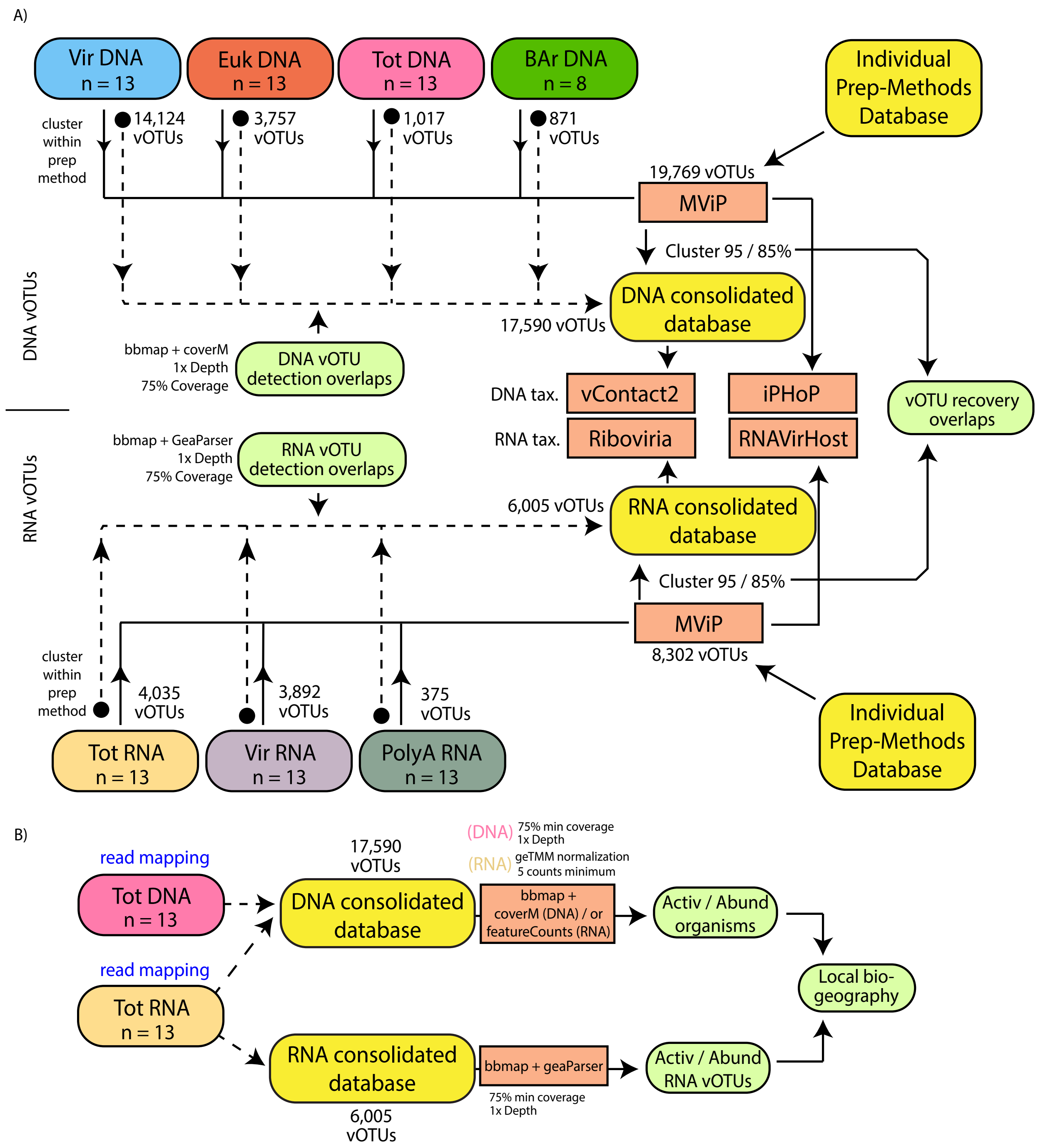

Supplementary Figure 2: Conceptual diagram of computational workflow used for manuscript. A) Top of figure shows the process that was done for the comparisons of the different methods. **B)** Bottom of the figure shows what was done for the ecological interpretation analyses. For vOTUs in both (A) and (B), we applied a 10kb size cutoff for DNA vOTUs, and no size cutoff RNA vOTUs. Only RNA vOTUs that had ssRNA + or - sense genomes were considered. Both genome types had to have a VirScore $\geq$ 0.7 and at least as many viral genes as host genes. vOTUs that did not agree with data type (i.e., DNA genome in RNA) were removed as well. Viral contigs are clustered at 95% ANI across 85% of shortest contig.

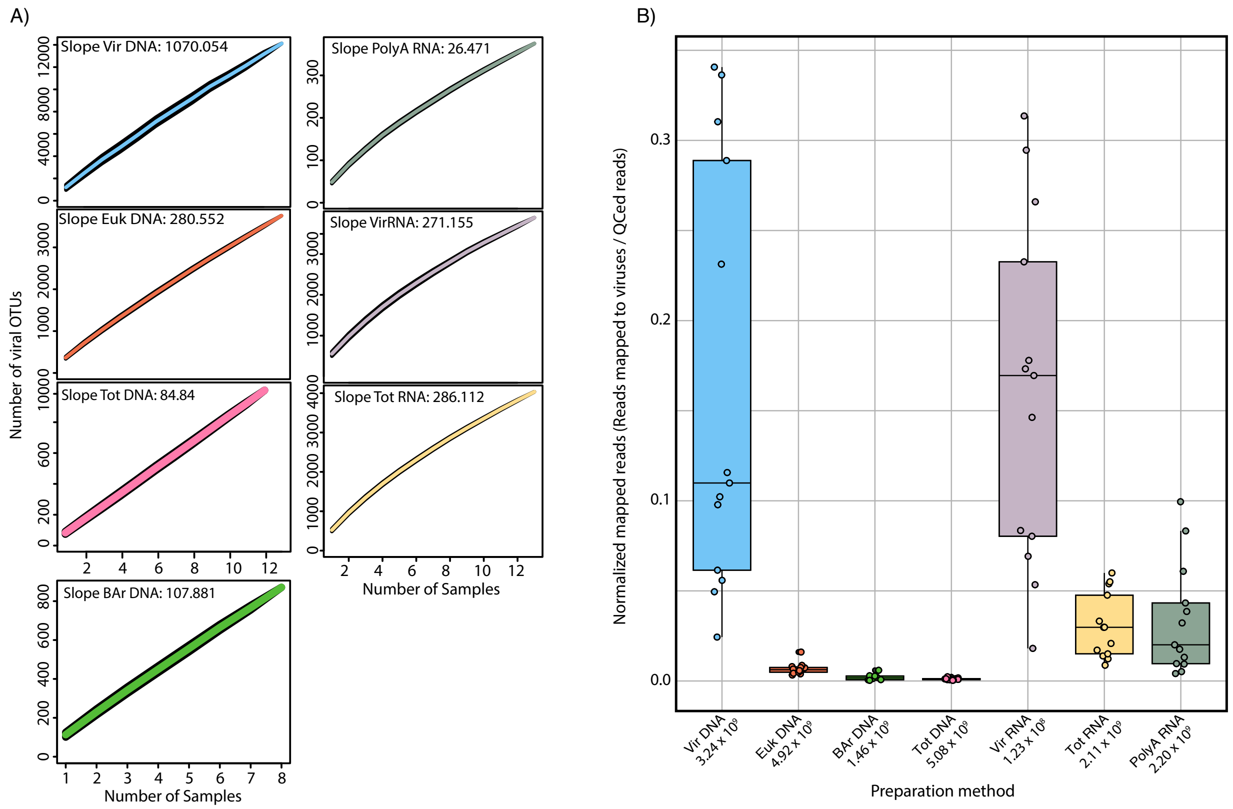

Supplementary Figure 3: Preparation methods vary in their ability to acquire viral sequences A) Viral accumulation plots highlight how many viral contigs (y-axis) are found per each sample (x-axis) across methods (colors). Confidence intervals are denoted as the width of each line, and the slope is denoted. **B)** The total number of quality-controlled reads from each method that map to the corresponding viral contigs recovered from each method. Colors denote each method, and total number of quality-controlled reads per method are shown under x-axis labels. Boxes represent the interquartile range (IQR) with the median shown as the center line; whiskers extend to the most extreme values within 1.5 × IQR of the first and third quartiles (Tukey method). All preparations have 13 biological replicates except for BAr DNA (8 samples).

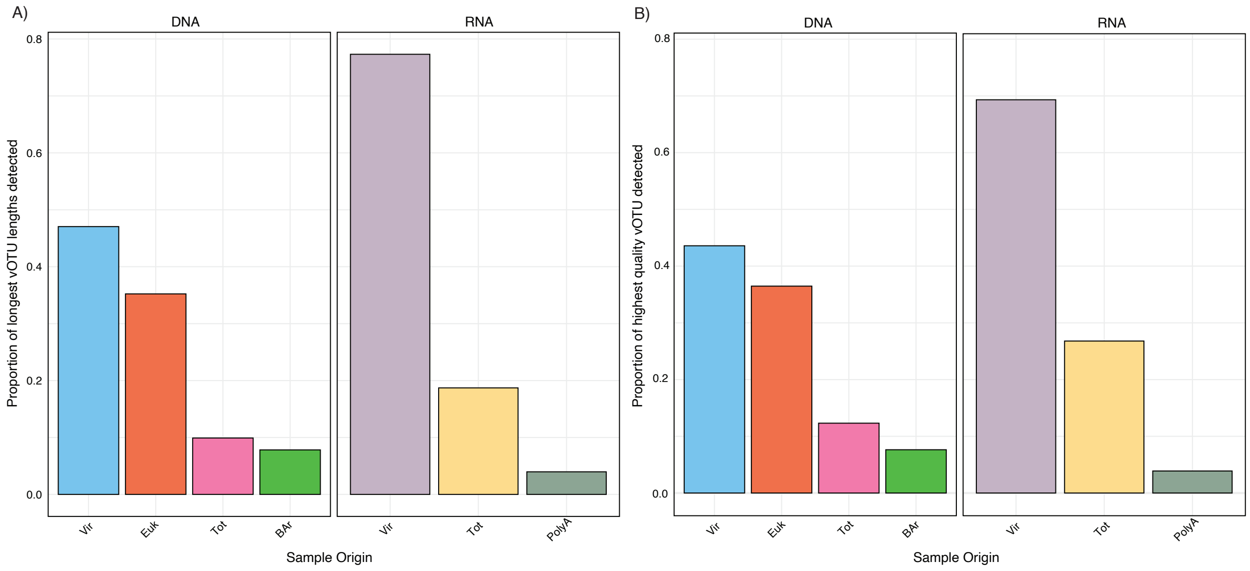

Supplementary Figure 4. Viral DNA and RNA yield longer and higher quality vOTUs than other methods. Boxplots are colored by preparation method and show the proportion of vOTUs pertaining to each preparation method that have the longest A) lengths or B) quality of overlapping viruses.

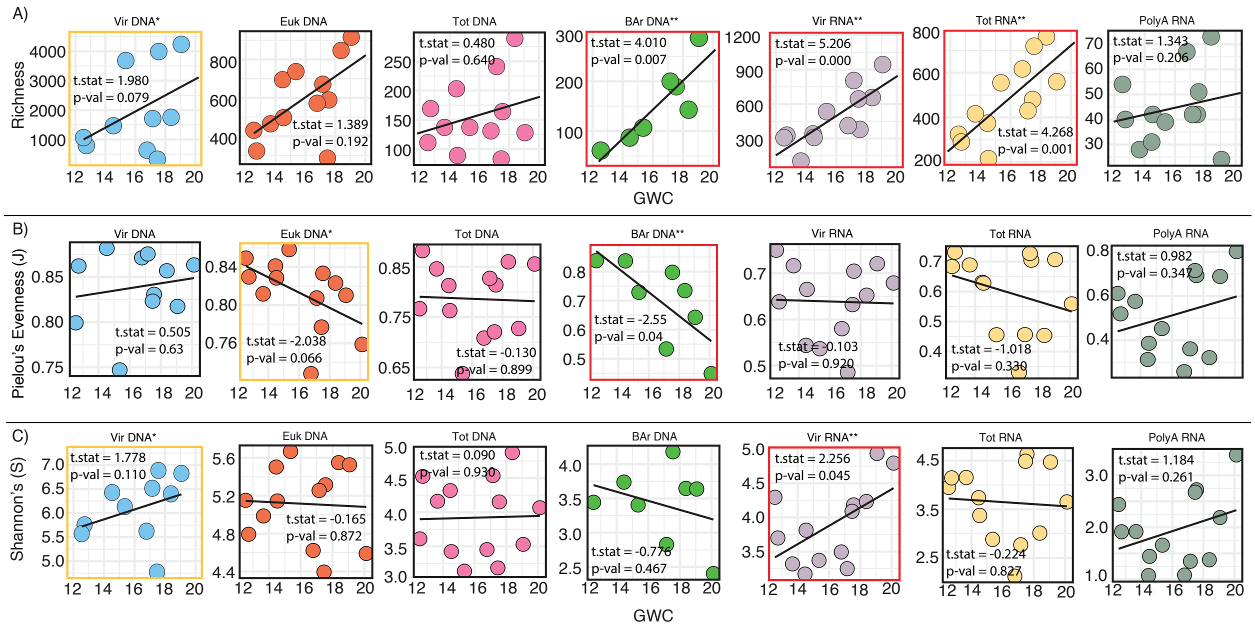

Supplementary Figure 5. Viral richness, Pielou's evenness, and Shannon's diversity across preparation methods and moisture. Scatterplots show the measured moisture (Y-axis) and the overall **A)** richness (total counts) **B)** Pielou's evenness, or **C)** Shannon's diversity, of viral contigs that originated from each preparation method. Significance of trends was assessed by fitting separate linear models for each preparation method; reported p-values correspond to two-sided tests of the slope term, and no multiple-comparison correction was applied. Significance values are shown in the text boxes and colored by preparation method. Significance in differences by fitting a linear model are shown with red boxes as significant (p ≤ 0.05), and yellow boxes as marginally significant (p ≤ 0.1), with values denoted within each plot.

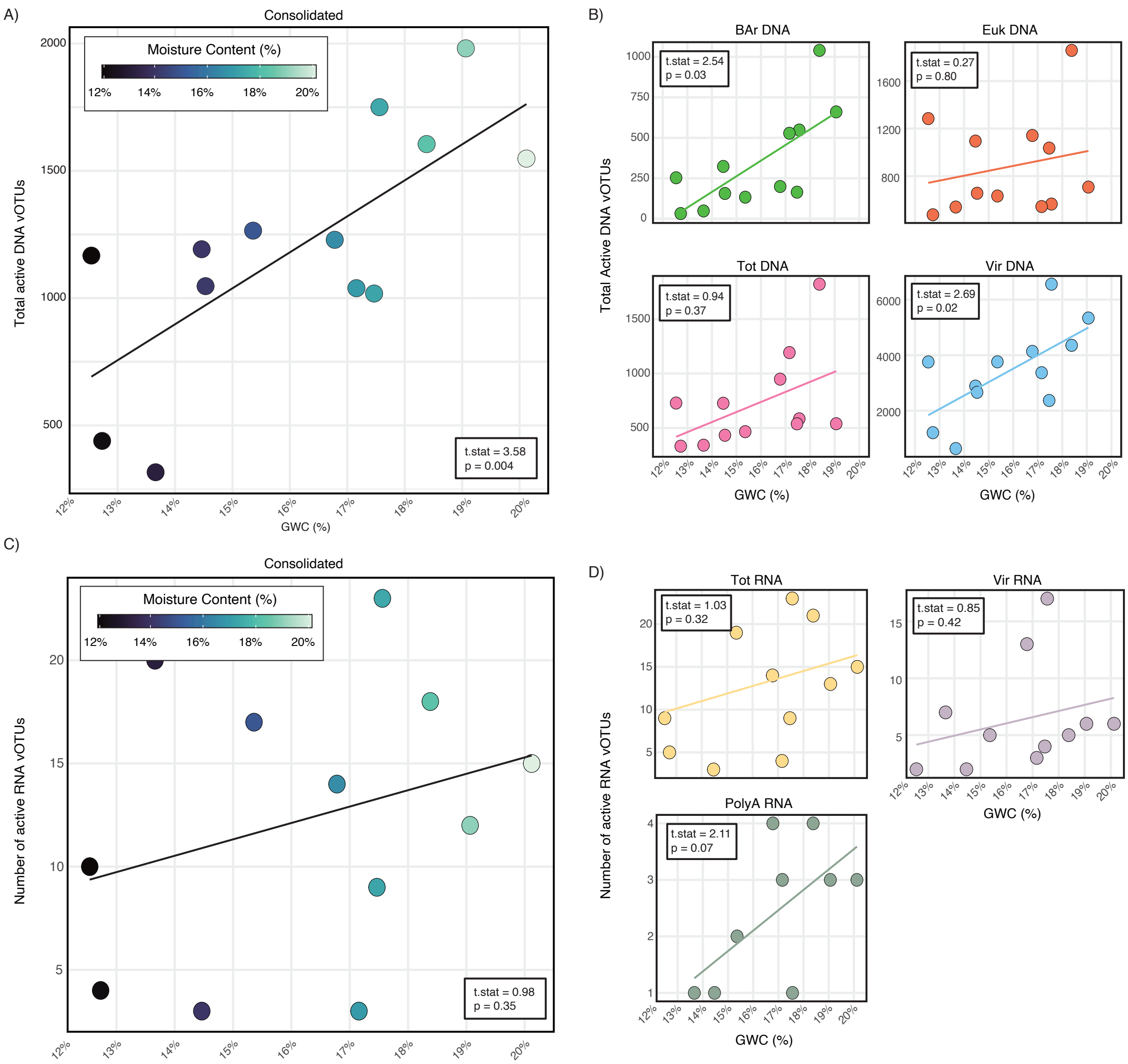

Supplementary Figure 6. Total number of active vOTUs for DNA and RNA datasets. Scatterplots denote the relationship between the number of active vOTUs and moisture for either DNA or RNA viruses. Linear fit statistics are denoted in boxes within each plot. Linear model statistics are shown in boxes within each plot; reported p-values correspond to two-sided tests of the slope term, and no multiple-comparison correction was applied. **A)** Number of vOTU trends detected across the clustered database of 17,590 DNA vOTUs. **B)** Number of vOTU trends detected across the individual viruses that were identified across each preparation method and colored by DNA preparation method. **C)** Number of vOTU trends detected across the clustered database of 8,302 RNA vOTUs. **D)** Number of vOTU trends detected across the individual viruses that were identified across each preparation method and colored by RNA preparation method.
