# Supplementary material for: Preparation method shapes the recovery and ecological interpretation of DNA and RNA soil viral communities
Source: Nat Commun. 2026 Jul 28;17:7551. doi: 10.1038/s41467-026-74154-1 (PMC13415888; doi:10.1038/s41467-026-74154-1)
Supplement: Supplementary file 2 — Description of Additional Supplementary Files [file 41467_2026_74154_MOESM2_ESM.docx]

**Description of Additional Supplementary Files**

Data S1: Sample Metadata Sheet: Metadata for each site, Metadata for each sample, Stats for read mapping results, Project IDs and sample metadata from JGI for data accessibility, and Collected biogeochemical measurements from each sample.

Data S2: vOTU Tracking Sheet: Viral information including All vOTUs from DNA Virome, All vOTUs from RNA Virome, All vOTUs from DNA Bacterial, All vOTUs from DNA EukFloat, All vOTUs from RNA Bulk, All vOTUs from RNA PolyA, Clustering dataframe of vOTUs from each method after being clustered across all methods to detect the vOTU overlap across methods, Taxonomic assignments via phylogenetic trees for the 8,335 RdRps encoded in 8,302 RNA vOTUs from all data methods, Masterlist downloaded from ICTV on October 20, 2024. Specifically, file version: ICTV_Master_Species_List_2023_MSL39.v3.xlsx (https://ictv.global/msl), Results from virus host matching of DNA viruses, and Results from virus host matching of RNA viruses

Data S3: vContact2 Results: Sheet containing the vContact2 output for all DNA vOTU taxonomic groupings.
