# Supplementary material for: Preparation method shapes the recovery and ecological interpretation of DNA and RNA soil viral communities
Source: Nat Commun. 2026 Jul 28;17:7551. doi: 10.1038/s41467-026-74154-1 (PMC13415888; doi:10.1038/s41467-026-74154-1)
Supplement: Supplementary file 6 — Reporting Summary [file 41467_2026_74154_MOESM6_ESM.pdf]

nature portfolio

Corresponding author(s): William C. Nelson

Last updated by author(s): Apr 24, 2026

Reporting Summary

Nature Portfolio wishes to improve the reproducibility of the work that we publish. This form provides structure for consistency and transparency in reporting. For further information on Nature Portfolio policies, see our Editorial Policies and the Editorial Policy Checklist.

Statistics

For all statistical analyses, confirm that the following items are present in the figure legend, table legend, main text, or Methods section.

n/a	Confirmed	
☐	☒	The exact sample size (n) for each experimental group/condition, given as a discrete number and unit of measurement
☐	☒	A statement on whether measurements were taken from distinct samples or whether the same sample was measured repeatedly
☐	☒	The statistical test(s) used AND whether they are one- or two-sided<br>*Only common tests should be described solely by name; describe more complex techniques in the Methods section.*
☐	☒	A description of all covariates tested
☐	☒	A description of any assumptions or corrections, such as tests of normality and adjustment for multiple comparisons
☐	☒	A full description of the statistical parameters including central tendency (e.g. means) or other basic estimates (e.g. regression coefficient) AND variation (e.g. standard deviation) or associated estimates of uncertainty (e.g. confidence intervals)
☐	☒	For null hypothesis testing, the test statistic (e.g. F, t, r) with confidence intervals, effect sizes, degrees of freedom and P value noted<br>*Give P values as exact values whenever suitable.*
☒	☐	For Bayesian analysis, information on the choice of priors and Markov chain Monte Carlo settings
☐	☒	For hierarchical and complex designs, identification of the appropriate level for tests and full reporting of outcomes
☒	☐	Estimates of effect sizes (e.g. Cohen's d, Pearson's r), indicating how they were calculated

Our web collection on statistics for biologists contains articles on many of the points above.

Software and code

Policy information about availability of computer code

Data collection

Sample collection and processing
Soil cores were collected on 18 Oct 2022 and 7 March 2023 from plots within the highest (100%) and lowest (25%) irrigation treatments, including 3 field replicates per treatment. All sampled plots were planted with the Alkar cultivar, except one plot with the Jose cultivar (Plot 40, 100% irrigation treatment) which was re-sampled in the spring for consistency, resulting in unequal sample numbers between collection efforts (n = 6 for October 2022, n = 7 for March 2023). Within each plot, one core (5cm diameter) was collected from a random location down to 15 cm depth. The 0-5 cm portion was discarded to remove the surface litter layer. The 5-15 cm portion of each soil core was aseptically broken up and a subsample for Tot RNA was snap frozen in liquid nitrogen (and stored at -80C prior to RNA extraction). The remaining soil was transported from the field site to the Pacific Northwest National Laboratory (PNNL) on ice for processing.

In the laboratory, 2mm sieves were used to homogenize soil and remove large roots and rocks prior to subsampling. All subsampling was completed the same day as sample collection. Subsamples for bacterial and archaeal (BAr), fungal (Euk), and viral (Vir) fractionation were stored at 4C until further processing. Soil for DNA extraction were stored at -80C until processing. The soil water content was measured by the gravimetric method [61] for each sample. Briefly, 10 g of soil was dried at 60C until a stable weight was achieved [62]. Gravimetric water content (GWC) was calculated as the fresh soil weight minus the dry soil weight, relative to fresh soil weight. pH was determined in 1:2 soil water slurries according to [63]. Sample metadata is available in Table S1.

Sample fractionation for bacteria/archaea, fungal hyphae, and viruses
Bacterial and archaeal cells were separated from total soil using a Nycodenz density gradient [64]. 15 mL of 1X PBS with 0.01 % Tween20 was added to 10 g soil and vortexed for 15 min. After allowing the soil slurry to settle for 5 min, supernatant was transferred to a new tube and diluted with 4 mL of the PBS/Tween solution. The slurry was vortexed again and allowed to settle before the supernatants were pooled. Two layers of Nycodenz were added below the supernatant: 5 mL of 40% Nycodenz then 2 mL of 80% Nycodenz below that. Gradients were

centrifuged at 5,000 x g for 15 min at 4C with a slow ramp and slow brake. The top two layers (aqueous phage and 40% Nycodenz) were transferred to separate tubes and diluted with an equal volume of 1X PBS. Cells were pelleted by centrifuging at 7,000 x g for 15 min, resuspended in 0.1 mL 1X PBS, and combined. Extracted cells were stored at -20°C until DNA extraction.

Fungal hyphae (and fine roots) were separated from total soil using a modified hyphal float approach [65]. 30 mL of 4 M KCl was added to 10 g soil and gently inverted to mix for 5 min. Slurries were allowed to settle for 1 min to sediment soil before decanting the supernatant with hyphae to a clean tube. Soil was re-extracted once with 4 M KCl and twice with 2 mL sterile DI water. Hyphae were collected by filtering pooled supernatants through 40 µm mesh and transferred to microcentrifuge tubes with sterile tweezers. Extracted hyphae were stored at -80C until DNA extraction.

Extracellular DNA and RNA viruses were separated from total soil following the Soil Viromics Protocol from the Emerson lab group (https://www.protocols.io/view/soil-viromics-protocol-emerson-lab-v1-kxygxz7q4v8j/v1) with modifications inspired by Hillary et al. [38]. For each sample, 120 g of soil was evenly distributed (20 g each) into 50 mL tubes and extracted with 18 mL PPBS buffer (2% BSA, 10% PBS, 1% K-citrate, 150 mM $MgSO_4$). Slurries were shaken at 300 rpm for 10 min at 4C and centrifuged at 4000 x g for 10 min at 4C in swinging bucket rotor. Supernatant was decanted to a clean tube and pooled with the supernatants of two subsequent extractions. Supernatant was decanted to a clean tube, and the extraction steps were repeated two additional times for each soil sample. Supernatants were pooled resulting a final volume of approximately 54 mL per sample. Pooled supernatants were centrifuged at 10,000 x g for 8 min at 4C in a fixed angle rotor and filtered through 0.22µm PES (Thermo Scientific) to remove microbial cells. To pellet viruses, cell-free supernatants were ultracentrifuged at 35,000 x g for 3 hours at 4C under vacuum. Supernatant was decanted, taking care not to disturb the viral pellet. Each pellet was resuspended in 100 µL cell culture grade water and DNase-treated following manufacturer recommendations (Promega RQ1 RNase-Free Dnase). Resuspended and pooled viral pellets were stored at -80C until further extraction.

Nucleic acid extraction (DNA and RNA)
DNA was extracted from bacterial/archaeal cells (BAr), and fungal hyphae (Euk), and soil (Tot-DNA) using the Quick-DNA Fecal/Soil Microbe Miniprep Kit (Zymo) according to manufacturer instructions. DNA yield was quantified by Qubit DNA High Sensitivity assay (Invitrogen) and purity was checked by NanoDrop spectrophotometry. Extracted DNA was stored at -80ºC prior to being shipped for sequencing. Tot RNA (2 g) was extracted with the RNeasy PowerSoil Total RNA kit (Qiagen) according to manufacturer instructions. The resulting RNA was DNase treated with the TURBO DNA-free™ Kit (Invitrogen) according to manufacturer instructions. Extracted RNA was quantified using the Qubit RNA High Sensitivity assay (Invitrogen), purity was checked by NanoDrop spectrophotometry, and quality was assessed by RNA 6000 Nano Kits (Agilent). Samples were stored at -80ºC prior to being shipped for sequencing. RNA and DNA were co-isolated from resuspended viral pellets using the RNeasy PowerSoil Total RNA Kit (Qiagen) in combination with the RNeasy PowerSoil DNA Elution Kit (Qiagen) and quantified as described above. Extracted nucleic acids were shipped to the JGI for metagenome and metatranscriptome library prep and sequencing, in support of user proposal 509015. Sample metadata can be found on Table S1.

Data analysis

Code used for processing and generation of figures is available on GitHub: https://github.com/jrr-microbio/interkingdom_virus/tree/main

For manuscripts utilizing custom algorithms or software that are central to the research but not yet described in published literature, software must be made available to editors and reviewers. We strongly encourage code deposition in a community repository (e.g. GitHub). See the Nature Portfolio guidelines for submitting code & software for further information.

Data

Policy information about availability of data

All manuscripts must include a data availability statement. This statement should provide the following information, where applicable:

- Accession codes, unique identifiers, or web links for publicly available datasets
- A description of any restrictions on data availability
- For clinical datasets or third party data, please ensure that the statement adheres to our policy

Metadata, viral genomes, microbial and archaeal genomes, as well as sample metadata are publicly available at PNNL DataHub [87]. Raw reads for all data are available on the JGI data portal (https://data.jgi.doe.gov/) under project IDs: PolyA RNA: 1440986-1440991 and 1441011-1441017, Tot RNA: 1440986-1440991 and 1441011-1441017, Euk DNA: 1441083-1441088 and 1441096-1441102, BAr DNA: 1441077, 1441079, 1441081-1441082, 1441091, and 1441093-1441095, Vir DNA: 1441129-1441141, Vir RNA: 1440998-1441010. All trimmed reads are publicly available on the JGI Data Portal and the PNNL DataHub (https://data.pnl.gov/group/7/nodes/dataset/37100). Protein fasta files used for phylogenetic analysis as well as all supplemental code are available on GitHub (https://github.com/jrr-microbio/interkingdom_virus) [81].

Research involving human participants, their data, or biological material

Policy information about studies with human participants or human data. See also policy information about sex, gender (identity/presentation), and sexual orientation and race, ethnicity and racism.

Reporting on sex and gender	n/a
Reporting on race, ethnicity, or other socially relevant groupings	n/a
Population characteristics	n/a
Recruitment	n/a
Ethics oversight	n/a

Note that full information on the approval of the study protocol must also be provided in the manuscript.

Field-specific reporting

Please select the one below that is the best fit for your research. If you are not sure, read the appropriate sections before making your selection.

☐ Life sciences ☐ Behavioural & social sciences ☒ Ecological, evolutionary & environmental sciences

For a reference copy of the document with all sections, see nature.com/documents/nr-reporting-summary-flat.pdf

Ecological, evolutionary & environmental sciences study design

All studies must disclose on these points even when the disclosure is negative.

Study description

To enhance detection of sub-communities of DNA and RNA viruses, we applied fractionation approaches to soils collected across a moisture gradient from a grassland field experiment. Analyses included metagenomics and metatranscriptomics of size-fractionated extracellular viruses (i.e., DNA and RNA viromes), metagenomics of bacteria/archaea- or eukaryote-enriched samples, and whole soil metatranscriptomes with rRNA-depletion or polyadenylation enrichment.

Research sample

Field Site management

Samples were collected from the Tall Wheatgrass Irrigation Field Trial in Prosser, WA, USA (46°15′04″N and 119°43′43″W), operated by Washington State University, and described in our previous publications [56–58]. The site is characterized by marginal Aridisol soils with low organic matter content (<2%), pH of 8, and a sandy loam texture (55.5% sand, 34.1% silt, 10.4% clay). Specifically, it is described as coarse-silty, mixed, superactive, mesic Xeric Haplocambids and having high porosity, permeability, and soil bulk density (avg = 1.56g/cm3) [53]. Tall wheatgrass (Thinopyrum ponticum), which is drought tolerant and adapted for growth on marginal soil, was established in May 2018, prior to which the site was uncultivated desert shrub-steppe. Plants are uniformly distributed within plots. Irrigation treatments have been ongoing since spring 2019. Irrigation is provided through drip lines from April to October with water supplied at four levels (100%, 75%, 50%, and 25% field water capacity) to create plots with differing water stress based on modeled crop evapotranspiration of tall wheatgrass [59, 60]. Each experimental plot is 2.1 m × 10.7 m with a 1.5 m alley between adjacent plots (Figure S1).

Sampling strategy

Samples were collected based on plot identity across a transect of soil which had differential moisture treatments applied.

Data collection

Sequencing and library preparation methods (DNA and RNA)

Samples were collected as described in the Methods by Sheryl Bell and others via field sample collection. DNA sequencing data were generated at the DOE Joint Genome Institute (JGI) using Illumina technology. For Virome DNA, an Illumina Low Input (DNA) library was constructed and sequenced using the Illumina NovaSeq X platform. For Tot, Euk and BAr DNA, an Illumina regular (DNA) library was constructed using the Illumina NovaSeq X platform. JGI's standard computational pipeline was used to process the reads. Briefly, BBDuk (version 39.01) [66] was used to remove contaminants, trim reads that contained adapter sequence and homopolymers of Gs of size 5 or more at the ends of the reads, remove optical duplicates from data generated on the NovaSeq X platform, and right quality trim reads where quality drops to 0. BBDuk was used to remove reads that contained 4 or more 'N' bases, had an average quality score across the read less than 3 or had a minimum length ≤ 51 bp or 33% of the full read length. Reads containing Swift sequences were trimmed from the left or right of the read. Reads mapped with BBMap to masked human, cat, dog and mouse references at 93% identity were removed. Reads aligned to common microbial contaminants were removed.

RNA samples extracted from total soil were split and treated with poly-A selection to enrich for eukaryote RNA (PolyA RNA) or rRNA depleted (Tot RNA) with Qiagen FastSelect kit with pooled bacterial, yeast, and plant probes to enrich for bacterial/archaeal mRNA prior to sequencing. RNA sequencing data were generated at the DOE Joint Genome Institute (JGI) using Illumina technology. For Virome RNA, an Illumina Ultra-Low Input (RNA) library was constructed and sequenced using an Illumina NovaSeq X 270 bp fragment platform and a TruSeq RNA library preparation kit. For PolyA and Tot RNA, an Illumina low input (RNA) library was constructed and sequenced using an Illumina NovaSeq X 270 bp fragment platform and a TruSeq RNA library preparation kit. BBDuk (version 39.01) [66] was used to remove contaminants, trim reads that contained adapter sequence and homopolymers of G's of size 5 or more at the ends of the reads, remove optical duplicates from data generated on the NovaSeq X platform, and right quality trim reads where quality drops to 0. BBDuk was used to remove reads that contained 1 or more 'N' bases, had an average quality score across the read less than 10 or had a minimum length ≤ 51 bp or 33% of the full read length. Reads mapped with BBMap to masked human, cat, dog and mouse references at 93% identity were removed as well as reads aligned to common microbial contaminants, ribosomal RNA reads, or known spike-ins. All sample metadata can be found on Table S1.

Timing and spatial scale

Soil cores were collected on 18 Oct 2022 and 7 March 2023 from plots within the highest (100%) and lowest (25%) irrigation treatments, including 3 field replicates per treatment, and 13 biogical replicates per preparation method except BAr DNA which had 8 biological replicates (5 samples failed).

Data exclusions

n/a

Reproducibility

All code is available on github: (https://github.com/jrr-microbio/interkingdom_virus)

Randomization

Samples were collected across different plot identities. No further groupings were used, and moisture values per each sample were used as a continuous variable.

Blinding

No need for blinding. Samples are simply collected from the field site and processed

Did the study involve field work? ☒ Yes ☐ No

Field work, collection and transport

Field conditions	Field Site management<br>Samples were collected from the Tall Wheatgrass Irrigation Field Trial in Prosser, WA, USA (46°15′04″N and 119°43′43″W), operated by Washington State University, and described in our previous publications [56–58]. The site is characterized by marginal Aridisol soils with low organic matter content (<2%), pH of 8, and a sandy loam texture (55.5% sand, 34.1% silt, 10.4% clay). Specifically, it is described as coarse-silty, mixed, superactive, mesic Xeric Haplocambids and having high porosity, permeability, and soil bulk density (avg = 1.56g/cm3) [53]. Tall wheatgrass (Thinopyrum ponticum), which is drought tolerant and adapted for growth on marginal soil, was established in May 2018, prior to which the site was uncultivated desert shrub-steppe. Plants are uniformly distributed within plots. Irrigation treatments have been ongoing since spring 2019. Irrigation is provided through drip lines from April to October with water supplied at four levels (100%, 75%, 50%, and 25% field water capacity) to create plots with differing water stress based on modeled crop evapotranspiration of tall wheatgrass [59, 60]. Each experimental plot is 2.1 m × 10.7 m with a 1.5 m alley between adjacent plots (Figure S1).<br><br>Sample collection and processing<br>Soil cores were collected on 18 Oct 2022 and 7 March 2023 from plots within the highest (100%) and lowest (25%) irrigation treatments, including 3 field replicates per treatment. All sampled plots were planted with the Alkar cultivar, except one plot with the Jose cultivar (Plot 40, 100% irrigation treatment) which was re-sampled in the spring for consistency, resulting in unequal sample numbers between collection efforts (n = 6 for October 2022, n = 7 for March 2023). Within each plot, one core (5cm diameter) was collected from a random location down to 15 cm depth. The 0-5 cm portion was discarded to remove the surface litter layer. The 5-15 cm portion of each soil core was aseptically broken up and a subsample for Tot RNA was snap frozen in liquid nitrogen (and stored at -80C prior to RNA extraction). The remaining soil was transported from the field site to the Pacific Northwest National Laboratory (PNNL) on ice for processing.<br><br>No data was excluded from our sample collection efforts. Soils were from a managed agricultural field site. Soil cores were collected as described with only exact amounts of soil extracted from the site as were necessary.
Location	Samples were collected from the Tall Wheatgrass Irrigation Field Trial in Prosser, WA, USA (46°15′04″N and 119°43′43″W)
Access & import/export	Samples were collected from the Tall Wheatgrass Irrigation Field Trial in Prosser, WA, USA (46°15′04″N and 119°43′43″W), operated by Washington State University, and described in our previous publications [44–46].
Disturbance	n/a

Reporting for specific materials, systems and methods

We require information from authors about some types of materials, experimental systems and methods used in many studies. Here, indicate whether each material, system or method listed is relevant to your study. If you are not sure if a list item applies to your research, read the appropriate section before selecting a response.

Materials & experimental systems

n/a	Involved in the study	
☒	☐	Antibodies
☒	☐	Eukaryotic cell lines
☒	☐	Palaeontology and archaeology
☒	☐	Animals and other organisms
☒	☐	Clinical data
☒	☐	Dual use research of concern
☐	☒	Plants

Methods

n/a	Involved in the study	
☒	☐	ChIP-seq
☒	☐	Flow cytometry
☒	☐	MRI-based neuroimaging

Dual use research of concern

Policy information about dual use research of concern

Hazards

Could the accidental, deliberate or reckless misuse of agents or technologies generated in the work, or the application of information presented in the manuscript, pose a threat to:

No	Yes	
☒	☐	Public health
☒	☐	National security
☒	☐	Crops and/or livestock
☒	☐	Ecosystems
☒	☐	Any other significant area

Experiments of concern

Does the work involve any of these experiments of concern:

No	Yes	
☒	☐	Demonstrate how to render a vaccine ineffective
☒	☐	Confer resistance to therapeutically useful antibiotics or antiviral agents
☒	☐	Enhance the virulence of a pathogen or render a nonpathogen virulent
☒	☐	Increase transmissibility of a pathogen
☒	☐	Alter the host range of a pathogen
☒	☐	Enable evasion of diagnostic/detection modalities
☒	☐	Enable the weaponization of a biological agent or toxin
☒	☐	Any other potentially harmful combination of experiments and agents

Plants

Seed stocks	n/a
Novel plant genotypes	n/a
Authentication	n/a
