# Supplementary material for: Preparation method shapes the recovery and ecological interpretation of DNA and RNA soil viral communities
Source: Nat Commun. 2026 Jul 28;17:7551. doi: 10.1038/s41467-026-74154-1 (PMC13415888; doi:10.1038/s41467-026-74154-1)
Supplement: Supplementary file 7 — Transparent Peer Review file [file 41467_2026_74154_MOESM7_ESM.pdf]

nature portfolio

Peer Review File

Preparation Method Shapes the Recovery and Ecological Interpretation of DNA and RNA Soil Viral Communities

Corresponding Author: Dr William Nelson

This file contains all reviewer reports in order by version, followed by all author rebuttals in order by version.

Version 0:

Reviewer comments:

Reviewer #1

(Remarks to the Author)
The paper by Rodríguez-Ramos et al. entitled "Comparisons of fractionation methods for DNA and RNA viruses unearths a multifaceted relationship between viruses and soil moisture" provides a comprehensive investigation into the utility of various fractionation methods in uncovering the complexity of soil viral communities. Through a detailed comparison of metagenomic and metatranscriptomic approaches, the authors explore how different methodologies influence the detection of DNA and RNA viral communities, particularly in relation to soil moisture gradients. The key findings suggest that the viral enrichment (VirFrac) methods generate higher quality and more complete viromes and enable the identification of viruses associated with different kingdoms. The integration of microbiome data with viral taxonomy and host prediction not only highlights the ecological significance of viruses in soil but also underscores the need for methodological considerations when examining microbial interactions. The paper is well written and the topic is timely. The paper focuses strongly on methodological aspects and could benefit from more analysis of ecological implications which would also highlight better the novelty of the findings.
While the findings are significant and contribute to ecological and metagenomic understanding, there are major points that should be addressed:
1. The description of the field site and sampling across moisture gradients is well-structured, but the exact methodologies, including the timing of sampling and the rationale for testing different methods, could be improved to provide a clearer context for the observations made.
2. The paper briefly discusses trade-offs between different fractionation methods, but further elaboration on how these trade-offs could affect the interpretation of ecosystem-level microbial interactions is lacking.
3. The authors compare the performance of methods based on quality and quantity, but a more in-depth comparison of how these factors might influence the ecological interpretation (and function) would add value to the discussion. Furthermore, it is not clear to me what would be a good "reference/benchmark".
4. The compilation of the consolidated database is mentioned, but a more detailed explanation of how this database was built and used would be helpful. Why not just use the "parental database" or the "consensus" sequences across methods?
5. Limitations which should be more clearly discussed or addressed. These include single field-site, unclear novelty (well-established patterns, e.g., viral diversity increases with moisture, emphasis on the utility of better sampling methods), and the ecological implications. For example, what do alter host-virus associations due to moisture entail? Are there different taxa/functions at different moisture levels?

Specific comments as I went through the text:
L12-21: The glossary is not very informative. The method designations (acronym?) could be more descriptive and clearer. E.g., why BarFrac? First, I thought these were names of new extraction kits. Adding fraction (Frac) seems redundant and floating (Float) is also just another method for (density) fractionation. Also, all are from soil, hence, designating soil is not necessary (it is also not consistent, later it is 'BulkSoil'/'WholeSoil'). Suggest to simplify to Vir, Euk, BAr, PolyA, and Bulk/Tot. Also, is it shrubland or grassland?
L24: [...] due to soil's high [...]
L25: "sub-communities" is mentioned only in the abstract and conclusions. It is not explained, defined, and elaborated further in the text. If it is a core aspect it should be mentioned.
L40: "exceedingly" -> exceeding what?
50-54: Is the problem really the sequence-based representation? I would think that the sequencing methods are powerful enough to discern microbial diversity. However, genome assembly (especially fungi), functional annotation and taxonomic

identity are challenging especially when dealing with different genomic compositions and fractionation techniques. Furthermore, the high number of taxa and number of possible interactions makes interpretation of the data challenging (regardless of data quality).
L53: Reproductive strategies are mentioned here and in the SI (workflow figure) but not further analyzed or discussed. This could provide a more insightful analysis of the ecological processes. e.g., are lytic/lysogenic vOTUs associated with specific functions?
L73-76: While I agree with the limitations of standard methods, applying enrichment methods can also bias the interpretation towards selected groups (e.g., how flotation affects fungal yeasts compared to hyphae?). Furthermore, the partitioning of biomass/DNA may need to consider the mass-conservation and account for different extraction efficiencies (which standard methods would be less prone to since everything stems from the same DNA/RNA sample). It is unclear to me from the selected methods what would be the reference approach? e.g., it would be useful to use the standard methods as reference.
L84-86: It seems this is out-of-scope for the current study (with one soil type) and should be rephrased.
L99: Why 'parent database'? Just 'database'? Also, the number of vOTUs is redundant as it appears in the text.
L100: I am missing the 'WholeSoil DNA', which could serve as a reference for the other methods (as a 'standard method'). Please explain why this was not included.
L115-117: This implies that none of the methods capture the diversity of viruses in the soil. Furthermore, the "BulkSoil" method indicates a similar relative slope as other methods - hence, capturing RNA-virus diversity similarly well to the others (even reaching higher vOTU counts - I guess this is meant by "number of viral populations"). How does the curve change with different thresholds for OTU clustering? i.e., how low does similarity need to be to reach saturation? Lastly, the figure axis should start at zero and use the same scale for the different methods to ease comparison.
Supplementary Figure 3: There seems to be a bi-modal distribution of virus (contig?) length in all methods. What could be the reason for the shorter lengths? Should they be filtered (and how would the proportions of low-quality change)? i.e., are the short ones of low quality and if so, could they be removed? Also, the axis should start at zero and the label or histogram should be moved to the other side.
L150: we could also expect bacterial viruses to be the most variable due to the nature of host propagation (high number of copies and possible mutations).
L192: Why was this fractionation selected? By absolute numbers of links it should be VirFrac. Also, what are the consensus matches across the methods?
L229: How were the moisture intervals classified? The range of variations is very narrow. How does it compare to the precision of the measurement (i.e., what was the SD of moisture values of thereplicates?). Only 10 g of soil was used, and it was dried only at 60 degrees C (typically it would be 105 degrees C for 24 or 48 hours).
L243: The 'consolidated database' contains more vOTUs than the 'parent' database. How is it possible? It also makes the designation of 'parent' a bit strange.
L247: Ambiguous. Higher in high moisture compared to low moisture?
L276: The header is long and does not convey much information. e.g., there are always "unique" and "shared"
L280: How robust is this with respect to filtering the data (i.e., using only the longer contigs)
L353-374: The finding seems consistent with what one would expect with higher host activities under higher moisture contents (especially, in water-limited systems). However, the range of moisture values is very narrow. This should be discussed. Are there other factors, which could underlie this pattern (e.g., vegetation, which could be more sensitive to the small differences in moisture with reduced carbon input at low moisture levels - as indicated by the NDVI in Fig. 1). Where there also plant viruses in the dataset? if so, how does their proportion change with moisture? Lastly, since the water comes from an irrigation system it could also be a source of viruses. Was the irrigation water analyzed as well? Alternatively, this could be tested by comparing the samples based on their distance to the water sources?
L436: Typically, water content is determined at 105 degrees C (above water boiling) for at least 24 h. Why was this temperature chosen?
L449: Please provide centrifugation speed in g.
L506-511: The quality threshold seems quite low. How do stricter quality setting affect the sequence recovery?
L687: Functional annotation was performed, but no analysis on the functions was reported.
Figure 1: The description on the foto is not useful - what does the acronym mean? It would be helpful to show a schematic of how the fractionation is done. Also, there are too many different font sizes and captions which could be placed on the figure caption
Figure 2: This figure is difficult to read and could be placed into the SI. It would be helpful to indicate (visually) what are in the different preparation methods. The DNA and RNA should be separated into two figures because they cannot overlap in the sets. It is not surprising that they are mostly non-overlapping since this is the goal of the fractionation. Visually, the figure is very busy with unclear elements and inconsistencies E.g., horizontal bars do not start at zero and font sizes are not consistent, bars in inset are not rounded others are, the "roundness" of borders changes, arrow showing "decreasing set size" is redundant as the axis reads "Set Size" etc.
Figure 3: The taxonomically assigned 2% seem more consistent across methods. Should this be discussed? Also, I do not understand why two different styles of visualization are chosen here. It would be clearer to represent panel B the same as panel A, which would also allow for clearer labelling of the taxa grouping (shared/unique).
Figure 5: It is a bit confusing why mostly bacterial viruses are recovered from different fractionation method. This should be discussed. In A, the label indicates total counts but no number. The caption should read: "Bacterial and archaeal phyla"? Also, I do not understand the proportions reported in panel B and D. The data is not compositional - how does it sum up to one across different methods? The method with the highest diversity just shows the highest proportion (apart from few unique groups)?
Figure 6: The figure seems unnecessarily busy due to multi-line labels, variably rounded edges, redundant labeling, and axis should start at zero. Also, displaying boxplots for the low number of samples is misleading. The estimation of quantiles is defined by a single value if the number of points is 5 - max, 75%, 50%, 25%, and min are a single sample. Plotting the median or mean would be better.

(Remarks on code availability)

Reviewer #2

(Remarks to the Author)
The manuscript “Comparisons of fractionation methods for DNA and RNA viruses unearths a multifaceted relationship between viruses and soil moisture”, submitted for consideration by Nature Communications, describes the sequencing of soil DNA and RNA viruses with prior fractionation by multiple approaches too generate a viral fraction, unicellular fraction and filamentaous (hyphal) fraction, as well as a whole soil transcriptome and polyA-enriched transcriptome. Sequencing outputs from these fractions are compared in various ways, notably for viral diversity and overlap, and predicted hosts. The parallel application of these approaches is certainly interesting, and the study advances the methodology in various ways that will interest researchers using or planning to use these approaches, especially with respect to RNA viromics. There are, however, some problematic aspects of the study. The chosen methods are not really comparable, in that they would not be expected to measure the same thing, but their differences are also not robustly validated. As such, it is not clear how to interpret the direct comparison presented in the manuscript. Secondly, use of field moisture content casts doubt on the ecological finding of the study, namely the influence of soil moisture on the virome. I details these below, followed by additional comments.

It is not clear why these methods were compared in this way. The size-fractionated viruses should in principle reflect extracellular viruses; the cellular bacterial fraction would presumably reflect intracellular prokaryotic viruses; the hyphal fraction presumably intracellular fungal viruses and intracellular viruses of fungal symbionts. There seems little a priori reason to expect these methods to deliver the same results, as they measure quite different viral pools. As such, it is not clear how a comparison of vOTU richness (l. 111-121; 225-238) and overlap (fig. 2) should be interpreted. At the same time, with the exception of limited evidence from predicted hosts, there is little validation that different target pools have been successfully selectively sequenced. It doesn’t seem appropriate to consider differences between the fractions to reflect bias (l. 155). Agreement between these methods does not seem to provide information about their methodological validity, since ecologically any outcome would be possible depending on the (unknown) distribution of viral taxa across these different pools. As such, while the comparison is certainly interesting, it lacks a solid theoretical basis to make be rigorous.

The study reported interesting trends and some significant differences between samples of differing moisture content. The analysis of soil moisture was problematic, however. The sampling site was a field experiment with irrigation treatments (l. 408-417), and the sampling design used these as treatments (Fig. 1) but in the end the analysis ignored this experimental design and it seems that field moisture at the time of sampling is instead used to re-classify samples on a categorical moisture gradient (l. 659-661), apparently disregarding the experimental moisture treatment or sampling date. The reasons for this are unclear. Crucially, however, this shifts the study from an experimental design to an observational one, where differences may be correlated to moisture content but not necessary caused by it. As just one example, I wonder whether the observed differences are not also related to soil organic matter, which quite likely correlates to field moisture (in as far as this is not driven by experimental irrigation). This undermines causal conclusions about the role of moisture (l. 358-359).

The manuscript has a tendency to show significant results while placing non-significant results in the supplementaries, but this risks a subtle publication bias. For example, determining viral richness under contrasting moisture was a major target for this study, but these results are not shown in the main text (although described in l. 226-238).

l. 243 What is holistic about this approach?

l. 236 This conclusion is unjustified based on the data. If methods did not detect statistically significant differences, then they should not be considered to have detected differences. Furthermore, this statement seems based on an a-priori assumption that the hypothesized differences were present. Perhaps they were not.

l. 53 Virus strategies are more diverse than this simple binary classification

l. 55 Language is odd here: can an organism “exist at an abundance” or “exist at lower biomass”?

l. 59 Unclear what “them” refers to

l. 65-66 Viruses are not examples of cells

l. 66 Separation methods don’t maximize the quantity present –as a general rule there’s a trade-off between separation purity and total recovery

l. 85 The authors emphasize the need to generalize previous observations across different soil types, but then this study looks only at a single site. Therefore, this doesn’t seem to be a gap that is filled by the study.

The study uses the term “community” very loosely, for example l. 96 “less studied viral communities” actually refers to operationally defined groups of viruses within the same soil microbial community.

Basic soil data is lacking: quatitative SOC content, quantitative texture, WRB soil type classification, land use and plant

cover prior to establishing the experiment.

l. 415 Was water supply adjusted to maintain the soils always at these moisture contents? Percentages of field capacity don't provide a reproducible water supply rate, so this unit of description is ambiguous.

l. 438 Conventionally, soil moisture content should be calculated relative to soil dry mass, bot wet mass.

l. 459 Greek letter mu should be used in the "micro" unit

l. 469-472 These sentences seem to repeat each other

l. 569 DNA?

Methods should also provide the raw sequencing depth for each dataset

l. 159 and elsewhere, this is a misuse of "n". This conventionally refers to a number of replicates, but the additional vOTUs added to the database (in this example) are not replicates in the context of this analysis. As such, the "n=" seems to add confusion rather than clarity here.

l. 198-199 This sentence is not so clear. What is a "host-phylum linkage profile"? And why is this analysis done at the phyla level? (I would argue if host linkage is only consistent at the phylum level, this is not consistent at all).

Fig. 6 Actual p values should be provided rather than just the $p < 0.05$ cutoff.

Fig S5 should show individual datapoints, like the figures in the main text.

l. 287-288 which biological insights are these?

l. 342-344 The argument is not so clear why lower diversity would suggest a "trove" of sequences.

l. 383 This is highly speculative, given that we know little about moisture-level responses of viral communities and the relevant data (water-filled pore space and connectivity) is not reported for the soil. The cited Bickel and Or (2020) consider climatic water content, integrating long-term selection processes at a site, and it is not clear how this could be compared to the snapshot of moisture content applied in this work. At least, this comparison is not explained or justified.

(Remarks on code availability)

Reviewer #3

(Remarks to the Author)
Rodrigues-Ramos et al report a comparison of the different methods that can be used to sequence the soil virome, with a particular emphasis on fractionation and RNA viruses sequencing. Sampling was done at 7 different locations in the same field, which varied in soil moisture.
According to their analysis, at the species level, there was barely any overlap among the viruses found with the various fractionation methods. However, at taxonomic ranks above family, among RNA viruses, some overlap existed. They also showed that ~ 40% of their DNA vOTU are new, compared to those present in soil virus databases. They finally determine, thanks to their RNA and DNA sequencing of the same samples, the amount of active viruses, defined as those having transcripts, and found it to be very low compared to the amount of viral genomes (but this could be due to technical problems). Nevertheless, on this small dataset, a tendency of higher viral activity in soils with higher moisture was detected.

For me, as a virome- but not a soil-specialist, this analysis is very methodological and technique oriented, and it is not bringing much novelty. However, soil viromes are notoriously difficult to analyse, and I can understand that this paper is a breakthrough in its field.

If the editor wants to accept it, I would recommend to improve the methods in the following directions:

-Clustering is made in two steps (lines 105 and 241), one of low quality, that serves to conclude for all results in figures 2 to 5, one of higher quality, that serves to conclude in figure 6. The high quality clustering should be used all along. Then to define which vOTU is present in which sample (for the important figure 2, for instance), a mapping step should be applied (using rarefied RNA,and rarefied DNA reads to compare samples at the same sequencing depth), including the removal of vOTU that collected insufficient reads (the cut-off 75% coverage and 1x depth is OK). The main difficulty with figure 2 as it is, is that some vOTU may be present in two or more samples, but assembly was not good enough to get it complete (as reflected in the CheckV result which is quite poor). So fragments 1 and 2 of the same vOTU appear as independent vOTU, and the overlap between methods is poor. This problem should be mentioned.

-Fig S2D indicates that the vOTU set is collecting a small fraction of the total reads used for assembly. This should be mentioned as a limit of the study, and commented. Could it be that a better assembler (such as Spades) would have given better results?

-Does the increase in vOTU richness with soil moisture simply reflect an increase in bacterial OTU (bOTU) richness? Authors have the sequencing data to analyse bOTU (but this analysis is not reported in the manuscript), if they do not intend to report this analysis, they should at least mention this interpretation.

-The Methods section 'nucleic acid extraction' should more clearly describe what is the "VirFrac" fraction, the same way as "BarFrac" and "FloatFrac" are described.

(Remarks on code availability)
The git hub contains raw data used to build up phylogenetic trees of RdRp, as well as parsing scripts.

Version 1:

Reviewer comments:

Reviewer #1

(Remarks to the Author)
The manuscript was greatly revised and many of my concerns were addressed. The authors improved the description of the work performed that (from my understanding) was done thoroughly. Some of the bolder claims were toned down, which improved the data interpretation. The manuscript remains very methodological and descriptive (a bit like a technical note) and I cannot fully appreciate the novelty. I can understand the challenges faced for recovering viral RNA/DNA and that this "groundwork" is required to advance the research on viruses in soil. While the manuscript provides a relevant and useful dataset, it is missing an impactful experimental design and outcome. Overall, the text requires language editing because some parts are difficult to follow and the text could be more engaging. While the manuscript and figures improved, there are several points to address.

Title: The title does not say much and feels like it is just stating the obvious: "if you measure different things you may arrive at different interpretations"
Abstract should be re-written.
L14: "[...] microbial community [...]" and remove "communities" before "of [...] viruses". Maybe a bit philosophical: Communities are interacting populations and I would not know how this applies to viruses, which require a host for interaction.
L19-21: revise sentence
L24-26: revise sentence. "[...] expression by moisture [...]"? "[...] and method [...]"? I cannot follow what is written here. What is/are the object/subject of the sentence?
L68: What are "Viral DNA and RNA omics"? metagenomics? metatranscriptomics?
L76: Soil texture is mentioned here although the study is based on a single soil... this should be revised.
L78: What is a "site-scale" approach? A site has no representative scale. It can be 1, 100, 1000, ... m2
L90: The header has numbers. It is not clear what is the importance of this. The header does not convey a message and is very descriptive.
L113: This seems overstated and should be rephrased. Recovery implies that we know what is there and are able to reconstruct the composition quantitatively. To address this one would need to spike the soil with a known mixture of viruses and then recover it from DNA/RNA extractions. Here the outcome is obvious and boils down to "different methods measure different things" (L120).
Table 1: This is a useful addition.
L429: The header should be rephrased. The database does not show anything - it is just data. Moisture cannot have a relationship with ecology - ecology is the study of relationships among living organisms and their environment while moisture/water content is the state of the system. Maybe "insights into viral ecohydrology" or "relationship with the ecological system". Also, it is important here that the grassland is managed.
L448: The range of moisture levels are very low (I know studies can blow up minor differences and find significance with enough data). For context, this could be the difference in water contents of a soil aggregate and the surrounding loose soil. The discussion is fine as it states the limitation, but it should be called a "trend" rather than "marginally significant".
L482: here the values are 11-18%, but earlier it was 11-17%

Additional comments on previous comments of reviewer #2:
The study reported interesting trends and some significant differences between samples of differing moisture content. The analysis of soil moisture was problematic, however. The sampling site was a field experiment with irrigation treatments (l. 408-417), and the sampling design used these as treatments (Fig. 1) but in the end the analysis ignored this experimental design and it seems that field moisture at the time of sampling is instead used to re-classify samples on a categorical moisture gradient (l. 659-661), apparently disregarding the experimental moisture treatment or sampling date. The reasons for this are unclear. Crucially, however, this shifts the study from an experimental design to an observational one, where differences may be correlated to moisture content but not necessary caused by it. As just one example, I wonder whether the observed differences are not also related to soil organic matter, which quite likely correlates to field moisture (in as far as this is not driven by experimental irrigation). This undermines causal conclusions about the role of moisture (l. 358-359).

>R#1: While the authors toned down the references to soil moisture, it is still prominently presented in the abstract and in figures. Removing the reference to differences between expression/DNA in the abstract would be better. Especially, because the trends do not seem fundamentally different given the uncertainty of the data. It is not surprising that "expression and diversity" should yield comparable results in the first place. Also, Fig 8/9 could be reduced (keep only few panels for illustration purpose and move the rest to SI) without "hiding" the lack of clear relationships.

The manuscript has a tendency to show significant results while placing non-significant results in the supplementaries, but this risks a subtle publication bias. For example, determining viral richness under contrasting moisture was a major target for this study, but these results are not shown in the main text (although described in l. 226-238).

>R#1: As mentioned, I am not sure putting too much emphasis on the moisture results is helpful, because there is not much conceptual interpretation. E.g, L358: "unique ecology that mirrors their bacterial hosts " is mentioned as an explanation, but does not provide any conceptual link. Why should bacteria (and their viruses) be affected? While I agree with the later interpretation on how soil moisture can affect the ecosystem (L434ff), it is not explained how this relates to viruses - e.g., how does the habitat/host distribution change and affect the viral transmissions, etc. - and this only appears later in the text (L456ff). Also, the discussion often repeats the results. I suggest shortening the discussion on moisture by combining the sections.

Basic soil data is lacking: quatitative SOC content, quantitative texture, WRB soil type classification, land use and plant cover prior to establishing the experiment.

>R#1: The soil characteristics should be briefly reported in the text (not just adding the citation) as these are important for understanding the context of eventual moisture-virus relations.

l. 415 Was water supply adjusted to maintain the soils always at these moisture contents? Percentages of field capacity don't provide a reproducible water supply rate, so this unit of description is ambiguous.

>R#1: It is called "water content at field capacity" and quantifies how much (capillary) water is held by the soil against gravitational (free) drainage. It is an often reported quantity that, however, strongly depends on the methodology used to determine its values. Here, not enough information is provided to assess what the numbers mean.

l. 438 Conventionally, soil moisture content should be calculated relative to soil dry mass, bot wet mass.

>R#1: For soil, it should be WC (w/w) = m_water/m_dry = (m_fresh-m_dry)/m_dry

l. 383 This is highly speculative, given that we know little about moisture-level responses of viral communities and the relevant data (water-filled pore space and connectivity) is not reported for the soil. The cited Bickel and Or (2020) consider climatic water content, integrating long-term selection processes at a site, and it is not clear how this could be compared to the snapshot of moisture content applied in this work. At least, this comparison is not explained or justified.

>R#1: This was addressed.

(Remarks on code availability)

Reviewer #3

(Remarks to the Author)
I am afraid my first and main methodological point has not been fully answered. The authors have performed read mapping on the consolidated database, as requested, but they did not perform the appropriate step of read rarefaction (they instead made a normalization per sequencing depth, which is different). To compare vOTU richness between samples, a clean rarefaction is important.

Here how to proceed: Rarefaction consists in the a posteriori and proportional reduction of the number of mapped reads per sample, to reach an equivalent number of mapped reads in all samples. This rarefaction and richness estimation is done after having discarded, in each sample, the vOTUs insufficiently covered (as you did). As an example: If the "poorer" sample has a total of 13455 reads mapped on all vOTUs, then all other samples should have their read numbers per vOTU reduced proportionally so that in total they also have 13455 reads mapped. And vOTU ending up having less than 1 read after rarefaction should not be counted in the richness estimate. The number of rarefied reads chosen used to compare all samples should be stated in the Methods. The Y axis unit in Figures 2B and 3B should display the number of vOTU found in each sample, so values above 1 are expected (numbers such as 10-4 or 10-5, as shown now, are not relevant).

In the text itself, the sentence line 128-131 is obscure, and probably inaccurate. It should be corrected. It states: "Contrary to the recovery-based analyses, this is less stringent approach that does not require the assembly of a full virus and can ultimately help determine whether the reported recovery differences between methods shown in Figure 2A and Figure 3A are due to enrichment of certain vOTUs or incomplete contig reconstruction between methods.": Why is this less stringent? It is on the contrary more sensitive. What is meant by "does not require assembly of a full virus"? Virome studies are never limited to the subset of full viruses (this would be a very low number of vOTUs). What I tried to explain in my former review was that by combining assembly results (which are always quite deficient, regardless of the tool used), you increase the

quality (the length) of the contigs retrieved. The consolidated database and rarefaction approach is the less incorrect method permitting to compare samples/methods of extraction. What you call the “recovery based” analyses are highly biased by the sequencing depth: a given sample sequenced twice, with a 10-fold sequencing depth difference, will give artificially different richness results.

(Remarks on code availability)

Version 2:

Reviewer comments:

Reviewer #1

(Remarks to the Author)
The revisions provided by the authors addressed my concerns and, from my point of view, improved the manuscript, which I can now recommend for publication.

(Remarks on code availability)

Reviewer #3

(Remarks to the Author)
The authors did not comply with my first request. They instead provide reference to an old debate about rarefaction of 16S-based assessments of OTU richness, which was also semantic, and was apparently solved in favour of rarefaction. Anyway, this debate is irrelevant with the method I proposed to apply, a method used in viral metagenomics and taking into account the fact that total DNA approaches will contain a large proportion of bacterial reads which should not be considered when comparing methods. Indeed, in the soil samples analyzed here, in average 19,6% of the “viral extraction” reads mapped by to the (consolidated?) viral DB, whereas only 0.1% of the “total DNA” reads mapped on this DB (if suppl table 1 “read_mapping_data” sheet is providing mapped reads against the consolidated DB). Using the method I proposed in the last round of review would probably have revealed a better performance of the total DNA approach.
The answer to my second comment is confusing and not satisfactory. The authors suggest that to apply a read mapping approach, you need to have first a reference viral database, which is incorrect. You can also do read mapping on de novo databases constructed by read assembly, as they have done here. They continue by saying that both approaches are useful, which is very confusing.

(Remarks on code availability)

Reviewer #4

(Remarks to the Author)
Thank you for reaching out regarding Reviewer 3’s suggestion on rarefaction. I have given this careful thought and wanted to share my assessment.

In my view, the rarefaction approach proposed by Reviewer 3 that suggests normalizing samples based on the number of reads mapped to the consolidated database rather than sequencing depth is not necessary for the purposes of this manuscript. This approach would effectively standardize comparisons around the viral fraction of the data, thereby focusing the analysis specifically on viral populations. While such normalization is appropriate when the goal is to make ecological inferences about viral communities, it does not appear to align with what authors are trying to demonstrate.

As I understand it, the authors’ main point from this particular analysis is to demonstrate that viral enrichment leads to an increased recovery of viral sequences, an expected but still useful result (Figure 2B and 3B; line 116-127). Applying the suggested rarefaction would not fundamentally change this conclusion, as the enrichment effect is likely to remain strong. However, it could alter relative comparisons among the different sample types (e.g., Euk,Tot, and Bar). Since the manuscript does not appear to draw specific conclusions from these relative differences (though I recommend confirming this), the additional analysis may not provide meaningful new insight.

For these reasons, I believe the current analytical approach is sufficient to support the authors’ conclusions, and the additional rarefaction step suggested by Reviewer 3 is not essential.

Also, note that I did not review Figure 8 and its conclusions. If R3 is worried about that part, where they seem to draw ecological inferences, the ask might be valid.

(Remarks on code availability)

Open Access This Peer Review File is licensed under a Creative Commons Attribution 4.0 International License, which permits use, sharing, adaptation, distribution and reproduction in any medium or format, as long as you give appropriate credit to the original author(s) and the source, provide a link to the Creative Commons license, and indicate if changes were made.
In cases where reviewers are anonymous, credit should be given to 'Anonymous Referee' and the source.
The images or other third party material in this Peer Review File are included in the article's Creative Commons license, unless indicated otherwise in a credit line to the material. If material is not included in the article's Creative Commons license and your intended use is not permitted by statutory regulation or exceeds the permitted use, you will need to obtain permission directly from the copyright holder.
To view a copy of this license, visit https://creativecommons.org/licenses/by/4.0/

REVIEWER COMMENTS

General response to reviewers

Thank you for the effort and time spent reviewing our manuscript. We are pleased that you recognized the importance of our work to the field and provided us with thoughtful feedback. We believe that addressing your concerns has significantly strengthened our manuscript. We note that due to our extensive revisions, we have included a marked-up version, tracking each change.

To address concerns about comparing the ecology of the DNA virus methods, we performed analysis on an additional, seventh preparation method (total DNA). This necessitated significantly revising every section in the manuscript to include these new results and comparisons. Second, we have implemented a second approach for identifying whether a specific viral contig was shared across methods. Previously we relied on a recovery-based approach (i.e., based on assembly and identification of viral contigs). We have added a detection-based approach (i.e., based on mapping reads to a reference database). Third, we have extensively expanded the results and discussion, as well as added or modified all figures to clearly highlight the benefits and caveats of each method and how it may impact interpretation of the soil viral ecology in this system. Fourth, we have added in a table into the discussion that discusses a-priori expectations from each method followed by a-posteriori results from our analyses. Finally, we have added an additional conceptual figure (Figure 1) that more clearly showcases the methods employed in this work as well as revised our computational pipeline figure in the supplemental results (Figure S2) to more clearly outline the work performed.

Once again, we thank you for your time and expertise.

Reviewer #1 (Remarks to the Author):

The paper by Rodríguez-Ramos et al. entitled "Comparisons of fractionation methods for DNA and RNA viruses unearths a multifaceted relationship between viruses and soil moisture" provides a comprehensive investigation into the utility of various fractionation methods in uncovering the complexity of soil viral communities. Through a detailed comparison of metagenomic and metatranscriptomic approaches, the authors explore how different methodologies influence the detection of DNA and RNA viral communities, particularly in relation to soil moisture gradients. The key findings suggest that the viral enrichment (VirFrac) methods generate higher quality and more complete viromes and enable the identification of viruses associated with different kingdoms. The integration of microbiome data with viral taxonomy and host prediction not only highlights the ecological significance of viruses in soil but also underscores the need for methodological

considerations when examining microbial interactions. The paper is well written and the topic is timely. The paper focuses strongly on methodological aspects and could benefit from more analysis of ecological implications which would also highlight better the novelty of the findings.

Thank you for taking the time to review our manuscript, and for recognizing our contributions to the field! We are grateful for the high level of feedback you provided. In response, we have substantially revised the manuscript to address your concerns. We feel the revised manuscript is more robust and will serve as an important resource for the field of soil viral ecology. Please find line-by-line responses to your comments below.

While the findings are significant and contribute to ecological and metagenomic understanding, there are major points that should be addressed:

1. The description of the field site and sampling across moisture gradients is well-structured, but the exact methodologies, including the timing of sampling and the rationale for testing different methods, could be improved to provide a clearer context for the observations made.

To improve understanding of what we did and why, we have made a new conceptual figure (**Figure 1**) that details the parallel fractionation methods that were performed on each soil core as well as a table of expectations and outcomes for each preparation method (Table 1). We have also simplified supplemental figure 1 (now Supplemental Figure 2), to clearly show the computational pipeline. Language throughout the manuscript has been edited to use consistent terms for each method for greater cohesiveness. Finally, we added a table to the discussion (Table 1) that specifies the rationale for comparing these methods. Specifically, we denote our a priori hypotheses for each method along with references supporting those statements and the results from each method (a-posteriori) and how they compared to our expectations.

With regards to rationale for timing of sampling, the October collection occurred immediately after the second aboveground biomass harvest and end of irrigation treatments (i.e., should be strong integrated effect of irrigation and plant growth), while the March collection represents pre-emergence of perennial Tall Wheatgrass after months without active irrigation (i.e., potential homogenizing effect of wet winters and minimal/distinct belowground plant influence).

2. The paper briefly discusses trade-offs between different fractionation methods, but further elaboration on how these trade-offs could affect the interpretation of ecosystem-level microbial interactions is lacking.

Thank you for this feedback. We have extensively re-worked the results, figures, and discussion portions of the manuscript to highlight potential trade-offs and their effects on interpretation. Specifically, we detail the effect of the different preparation methods on common ecology metrics like diversity (richness, Pielou's Evenness, Shannon's Diversity) and expression. Given the addition of a total metagenome dataset, we have also detailed how each DNA or RNA fractionation method compares to the "overall" total dataset.

3. The authors compare the performance of methods based on quality and quantity, but a more in-depth comparison of how these factors might influence the ecological interpretation (and function) would add value to the discussion. Furthermore, it is not clear to me what would be a good "reference/benchmark".

Please see response to #2 for changes made related to how fractionation methods can change or enhance our interpretation of soil viral ecology. Our intention with this paper was a more methods-driven approach, and we have modified the manuscript extensively to reflect that. As such, specifics regarding changes in functionality is beyond the scope of the work presented.

With regards to what would be a good "reference/benchmark", we have added in a seventh preparation method (total metagenomes) and use that alongside all the other viral information we gathered from our nucleic acid sequencing to give the best possible depiction of the viral communities and ecology of our soils. Nonetheless, we recognize and agree that more work should be performed to benchmark these results, for example testing extraction efficiencies of fractionation methods on model soil communities inoculated into real soils. We have added language to reflect this necessary further testing (see Lines 461-471).

4. The compilation of the consolidated database is mentioned, but a more detailed explanation of how this database was built and used would be helpful. Why not just use the "parental database" or the "consensus" sequences across methods?

We apologize that this was not clear in our initial version of the manuscript. To more directly interrogate how each individual method influences our ecological interpretation, we opted to remove co-assembly method from the work. This has significantly streamlined the methods that were implemented. We have updated the supplemental methods figure to reflect this (Figure S2). Further, per reviewer comments, we now use two forms of metric for comparisons: 1) the original recovery-based methods (i.e., the ability of a method to fully assembly and identify a specific viral contig) and 2) an additional detection based method (i.e., the ability of the reads from a specific method to detect a viral contig from a

consolidated reference database). We have updated the methods and workflow figure to reflect this. Please let us know if you have further recommendations to increase clarity.

5. Limitations which should be more clearly discussed or addressed. These include single field-site, unclear novelty (well-established patterns, e.g., viral diversity increases with moisture, emphasis on the utility of better sampling methods), and the ecological implications. For example, what do alter host-virus associations due to moisture entail? Are there different taxa/functions at different moisture levels?

We agree we should have added in more caveats to our discussions and our conclusions. We have made edits in the results, discussions, and conclusions to make clear that this work is from a single site with a single soil type, and that further research is needed to confirm our hypotheses. We have also constrained the claims we have made in the article to focus more deeply on the differences between preparation methods.

Specific comments as I went through the text:
L12-21: The glossary is not very informative. The method designations (acronym?) could be more descriptive and clearer. E.g., why BarFrac? First, I thought these were names of new extraction kits. Adding fraction (Frac) seems redundant and floating (Float) is also just another method for (density) fractionation. Also, all are from soil, hence, designating soil is not necessary (it is also not consistent, later it is 'BulkSoil'/'WholeSoil'). Suggest to simplify to Vir, Euk, BAr, PolyA, and Bulk/Tot. Also, is it shrubland or grassland?

We agree that your simplified naming scheme is more effective than what we had and have adopted it throughout the manuscript. We have removed the confusing glossary.

L24: [...] due to soil's high [...]

This text has been modified.

L25: "sub-communities" is mentioned only in the abstract and conclusions. It is not explained, defined, and elaborated further in the text. If it is a core aspect it should be mentioned.

We have changed all instances of sub-communities to “communities”.

L40: "exceedingly" -> exceeding what?

We have removed the word “exceedingly”.

50-54: Is the problem really the sequence-based representation? I would think that the sequencing methods are powerful enough to discern microbial diversity. However, genome assembly (especially fungi), functional annotation and taxonomic identity are challenging especially when dealing with different genomic compositions and fractionation

techniques. Furthermore, the high number of taxa and number of possible interactions makes interpretation of the data challenging (regardless of data quality).

Thank you for your comment. While we agree with you that genomic assembly is a bottleneck for disentangling soil diversity, one reason why assembly can be a bottleneck is because sequence-based read coverage of individual organisms is low. We have reworded the text in this introduction paragraph (see Lines 39-50). We also agree that interpretation of possible interactions is incredibly challenging! We have added text to this effect (see Lines 47-49).

L53: Reproductive strategies are mentioned here and in the SI (workflow figure) but not further analyzed or discussed. This could provide a more insightful analysis of the ecological processes. e.g., are lytic/lysogenic vOTUs associated with specific functions?

We completely agree that the reproductive strategy of the viruses would be an incredibly powerful and useful method to enhance our results. Unfortunately, due to the challenging nature of viral genomic data in soils, we did not detect enough full-length viral genomes to confidently statistically compare the differences in lifestyles detected across preparation methods or moisture gradient. We apologize that we had not modified the supplemental results and methods to reflect the change in work performed. We have added some text regarding viral lifestyles to the discussion in regard to how the BAr DNA could potentially help with these analyses and we build upon this response with appropriate references (see Lines 349-363).

L73-76: While I agree with the limitations of standard methods, applying enrichment methods can also bias the interpretation towards selected groups (e.g., how flotation affects fungal yeasts compared to hyphae?). Furthermore, the partitioning of biomass/DNA may need to consider the mass-conservation and account for different extraction efficiencies (which standard methods would be less prone to since everything stems from the same DNA/RNA sample). It is unclear to me from the selected methods what would be the reference approach? e.g., it would be useful to use the standard methods as reference.

We agree that the enrichment methods likely bias the interpretation towards selected groups, much in the same way that standard methods bias the interpretation towards selected groups (https://pmc.ncbi.nlm.nih.gov/articles/PMC11460016/#Sec17, https://pubmed.ncbi.nlm.nih.gov/33612831/). The purpose of our comparisons was to see if alternate methods could exceed, enhance, or corroborate the results from standard methods. With regards to what would be the reference method, we have added in a new preparation method (total DNA / total metagenome) to be able to use as this "reference". This way, we were able to compare the fractionated DNA and RNA on to the standard

“total” methods. We also would like to point out that all data is prepared from the same exact soil samples, please see the new **Figure 1**.

L84-86: It seems this is out-of-scope for the current study (with one soil type) and should be rephrased.

Fair point. We have rephrased this in a way that suggests we are part of the additional studies needed, but not the ONLY study that is needed. See Line 75-77.

L99: Why 'parent database'? Just 'database'? Also, the number of vOTUs is redundant as it appears in the text.

We have modified the text as well as Figure S1 to be clearer. To answer your question, we have a database containing each dereplicated vOTU coming from each preparation method (19,759 DNA vOTUs, 8,302 RNA vOTUs) (i.e., not redundant within a preparation method, but redundant across preparation methods). We then also have another database (our consolidated database) that is globally dereplicated across all methods (17,590 DNA vOTUs, 6,005 RNA vOTUs). In our original manuscript, we also had a third database that included co-assemblies of Vir DNA reads, which led to confusion regarding the term “parent” database. For simplicity and clarity, we have removed this database, as the core message of the manuscript did not depend on it, and it was confusing to follow. We now only refer to the individual preparations database and the consolidated (globally non-redundant) database.

L100: I am missing the 'WholeSoil DNA', which could serve as a reference for the other methods (as a 'standard method'). Please explain why this was not included.

Indeed! In response to reviewer comments, we have extracted DNA from the stored soil samples that our original preparation methods were made from. We have sequenced these and now include them in our results. Originally, we did not have the sequencing allocation as part of the user proposal that was awarded for all preparation methods. Since works comparing Total DNA and Virome DNA have been published before, we opted to focus on other comparisons initially. However, we reached out to the JGI user facility, and they graciously allowed us a larger allocation for this purpose.

L115-117: This implies that none of the methods capture the diversity of viruses in the soil. Furthermore, the "BulkSoil" method indicates a similar relative slope as other methods - hence, capturing RNA-virus diversity similarly well to the others (even reaching higher vOTU counts - I guess this is meant by "number of viral populations"). How does the curve change with different thresholds for OTU clustering? i.e., how low does similarity need to be to reach saturation? Lastly, the figure axis should start at zero and use the same scale for the different methods to ease comparison.

We agree that the accumulation curves suggest that neither method is getting us at the breadth of viral diversity in soils. We also agree that the Tot RNA is at least as effective regarding viral recovery as the rest of the RNA methods for RNA viruses from this study. We have further elaborated in the discussion as to the tradeoffs of Tot RNA and Vir RNA. Yes, Tot RNA identifies the highest number of "viral populations" as you mention. This is how they are referred to in the viral literature.

With regards to how the curve changes with different thresholds for OTU clustering, this is well beyond the scope of this work. The methods used to cluster are the standard, generally accepted cutoffs for viral OTUs (https://www.nature.com/articles/nbt.4306). These are made at that cutoff given they represent ecologically distinct populations. Our attempt at going beyond just the "OTU" level is reflected in our taxonomic assignment analyses at the Family level.

Per your request we have changed the y-axes on the figures to more clearly denote the '0' mark.

Supplementary Figure 3: There seems to be a bi-modal distribution of virus (contig?) length in all methods. What could be the reason for the shorter lengths? Should they be filtered (and how would the proportions of low-quality change)? i.e., are the short ones of low quality and if so, could they be removed? Also, the axis should start at zero and the label or histogram should be moved to the other side.

To streamline the messaging of the manuscript, we have removed the distribution of viral lengths from the figure and manuscript. The likely reason why there was a bi-modal distribution is that there are specific size cutoffs for DNA viruses where most viruses fall along. There are likely a handful of much longer viruses (megaviruses) but those were expected to be rare in these datasets. Shorter viruses do not inherently mean lesser quality. Please let us know if you have any further feedback.

L150: we could also expect bacterial viruses to be the most variable due to the nature of host propagation (high number of copies and possible mutations).

We recognize your point and mention that another reviewer also brought up this possibility. We have added in a second approach to identifying viral contig overlaps across methods (please see ***Overall Comments to Reviewers*** at the beginning of this document). Specifically, we refer to "recovery" and "detection" approaches as two unique ways to identify whether a virus is found in other samples. Recovery requires the entirety of the virus to cluster at the standard dereplication cutoffs. The detection approach, on the other hand, is much less "stringent", and only requires a minimum amount of reads from a preparation method to map/align to a virus present in the full consolidated database. This

makes it slightly more robust to these potential mutations. We note that both approaches gave us similar results, but we agree that it was an important point to verify.

L192: Why was this fractionation selected? By absolute numbers of links it should be VirFrac. Also, what are the consensus matches across the methods?

Yes, by absolute number of links Vir DNA had the highest number of links. However, VirFrac also had a substantially larger number of viral contigs. As such, we normalized the total number of links by the total number of viral contigs per preparation method. We noted that Vir DNA had the highest number of links, which we have now written explicitly per your comment, and also report descending order of normalized links (see Line 219).

L229: How were the moisture intervals classified? The range of variations is very narrow. How does it compare to the precision of the measurement (i.e., what was the SD of moisture values of the replicates?). Only 10 g of soil was used, and it was dried only at 60 degrees C (typically it would be 105 degrees C for 24 or 48 hours).

We have revised the analysis to use the measured moisture values as a continuous variable for regression analyses instead of grouping samples into arbitrary moisture categories. Yes, the overall range in measured soil moisture is narrow, but happily it is sufficient to detect some statistically significant trends (see Figures 8, Figure 9, and Figure S5 and S6 for examples).

L243: The 'consolidated database' contains more vOTUs than the 'parent' database. How is it possible? It also makes the designation of 'parent' a bit strange.

Thank you for noting this. Please see our responses to comments above made on L99. In our original submission, we had a third database that included co-assemblies of Vir DNA reads, hence why there were additional co-assembled viruses in the dataset, which led to confusion regarding “parent” database. For simplicity and clarity, we have removed this database as the core message of the manuscript did not depend on it.

L247: Ambiguous. Higher in high moisture compared to low moisture?

We have rewritten this entire section to more correctly depict the calculated statistics.

L276: The header is long and does not convey much information. e.g., there are always "unique" and "shared"

We have significantly rewritten the entire discussion and changed the paragraphs headers. Please let us know if you have any further comments.

L280: How robust is this with respect to filtering the data (i.e., using only the longer contigs)

We have modified our approaches to use both "recovery" and "detection" based methods to help account for the possibility of different sized viruses contributing to these results. Please see response to L150 above. We have rewritten the results to reflect this. See Lines 115-137.

L353-374: The finding seems consistent with what one would expect with higher host activities under higher moisture contents (especially, in water-limited systems). However, the range of moisture values is very narrow. This should be discussed. Are there other factors, which could underlie this pattern (e.g., vegetation, which could be more sensitive to the small differences in moisture with reduced carbon input at low moisture levels - as indicated by the NDVI in Fig. 1). Were there also plant viruses in the dataset? if so, how does their proportion change with moisture? Lastly, since the water comes from an irrigation system it could also be a source of viruses. Was the irrigation water analyzed as well? Alternatively, this could be tested by comparing the samples based on their distance to the water sources?

We agree that the finding is consistent with what we would expect at higher moistures. While the moisture value ranges were fairly narrow, we note that other studies have used similar ranges, for example this Nature publication: https://www.nature.com/articles/s41598-021-88914-0. We identified some plant viruses detected in the RNA preparations, specifically in the PolyA method. However, the current manuscript is written in the scope of methods comparisons. We have focused our efforts on these methods comparisons and reduced the claims relating to overall ecosystem services. Given limitations to funding and journal word limits, we will follow up with these in future works.

L436: Typically, water content is determined at 105 degrees C (above water boiling) for at least 24 h. Why was this temperature chosen?

Our soil samples are collected as part of a scientific focus area where multiple research questions are addressed from the same samples. While 105 degrees C is commonly used for soil drying, this temperature detrimentally affects total carbon and total nitrogen measurements. As such, we used the 60 degrees C temperature for the measurement, as is specified in the Kellogg Biological Station LTER protocol here: https://lter.kbs.msu.edu/protocols/24. We have added in this reference.

L449: Please provide centrifugation speed in g.

This correction has been made.

L506-511: The quality threshold seems quite low. How do stricter quality setting affect the sequence recovery?

The quality thresholds employed here and written out in the methods are the JGI DOE genomic workflow that has been benchmarked and tested. These methods were benchmarked and provided by JGI as part of their user facility protocols. We have added a reference to their protocol to make it clear that these cutoffs are coming directly from their protocols (https://journals.asm.org/doi/10.1128/msystems.00804-20?url_ver=Z39.88-2003&rfr_id=ori%3Arid%3Acrossref.org&rfr_dat=cr_pub++0pubmed)

L687: Functional annotation was performed, but no analysis on the functions was reported.

Initially, our goal was to use the annotation in the ecological assessments. However, due to the length and complexity of the current manuscript they were not included. We have removed the lines from the methods and revised Figure S1.

Figure 1: The description on the foto is not useful - what does the acronym mean? It would be helpful to show a schematic of how the fractionation is done. Also, there are too many different font sizes and captions which could be placed on the figure caption

We agree that figure 1 was not helpful in orienting the reader. We have replaced this with a conceptual diagram that explains the preparation methods that were used. We have also worked on formatting all figures and text sizes to be consistent.

Figure 2: This figure is difficult to read and could be placed into the SI. It would be helpful to indicate (visually) what are in the different preparation methods. The DNA and RNA should be separated into two figures because they cannot overlap in the sets. It is not surprising that they are mostly non-overlapping since this is the goal of the fractionation. Visually, the figure is very busy with unclear elements and inconsistencies E.g., horizontal bars do not start at zero and font sizes are not consistent, bars in inset are not rounded others are, the "roundness" of borders changes, arrow showing "decreasing set size" is redundant as the axis reads "Set Size" etc.

Per your recommendation, we have included a conceptual diagram to showcase the different preparation methods. We have also separated the DNA and RNA analysis into Figures 2 and 3, respectively. We have simplified the original upset plots and fixed the font sizes and the bounding boxes to keep things consistent.

Figure 3: The taxonomically assigned 2% seem more consistent across methods. Should this be discussed? Also, I do not understand why two different styles of visualization are chosen here. It would be clearer to represent panel B the same as panel A, which would also allow for clearer labelling of the taxa grouping (shared/unique).

We have removed the confusing Panel A given that the purpose was to show the overlaps of the 2% of taxonomically assigned viral contigs. We have opted for adding text into the results that highlight the high degree of non-overlap of viral genera that had no known taxonomic classification (See Lines 189-193).

Figure 5: It is a bit confusing why mostly bacterial viruses are recovered from different fractionation method. This should be discussed. In A, the label indicates total counts but no number. The caption should read: "Bacterial and archaeal phyla"? Also, I do not understand the proportions reported in panel B and D. The data is not compositional - how does it sum up to one across different methods? The method with the highest diversity just shows the highest proportion (apart from few unique groups)?

Other than viruses, bacteria are the most common microorganisms in soils. As such, it is understandable that all methods detect a significant portion of bacterial host links. We have modified the legend per your request, and we have split the figure into DNA and RNA subsets (Figure 6, Figure 7) to streamline the results and comparisons. Indeed, the data are just counts. But we normalize it two ways: in panels B we show by host Phylum the proportion of total host assertions by each method, while in panels C show the relative abundance of each Phylum asserted as host by each method.

Figure 6: The figure seems unnecessarily busy due to multi-line labels, variably rounded edges, redundant labeling, and axis should start at zero. Also, displaying boxplots for the low number of samples is misleading. The estimation of quantiles is defined by a single value if the number of points is 5 - max, 75%, 50%, 25%, and min are a single sample. Plotting the median or mean would be better.

Due to the changes in analysis (treating moisture as a continuous variable) this figure has been replaced with new figures 8 and 9.

Reviewer #2 (Remarks to the Author):

The manuscript “Comparisons of fractionation methods for DNA and RNA viruses unearths a multifaceted relationship between viruses and soil moisture”, submitted for consideration by Nature Communications, describes the sequencing of soil DNA and RNA viruses with prior fractionation by multiple approaches to generate a viral fraction, unicellular fraction and filamentaous (hyphal) fraction, as well as a whole soil transcriptome and polyA-enriched transcriptome. Sequencing outputs from these fractions are compared in various ways, notably for viral diversity and overlap, and predicted hosts. The parallel application of these approaches is certainly interesting, and the study

advances the methodology in various ways that will interest researchers using or planning to use these approaches, especially with respect to RNA viromics.

Thank you for the time and effort you spent reviewing our manuscript, and the recognition of our contributions to the field! We appreciate the depth of comments that you provided, and we believe they have significantly strengthened our manuscript. Below, we respond to your comments line by line, but we ask that you please review the entirety of the manuscript as it has been significantly rewritten.

There are, however, some problematic aspects of the study. The chosen methods are not really comparable, in that they would not be expected to measure the same thing, but their differences are also not robustly validated. As such, it is not clear how to interpret the direct comparison presented in the manuscript. Secondly, use of field moisture content casts doubt on the ecological finding of the study, namely the influence of soil moisture on the virome. I detail these below, followed by additional comments.

It is not clear why these methods were compared in this way. The size-fractionated viruses should in principle reflect extracellular viruses; the cellular bacterial fraction would presumably reflect intracellular prokaryotic viruses; the hyphal fraction presumably intracellular fungal viruses and intracellular viruses of fungal symbionts. There seems little a priori reason to expect these methods to deliver the same results, as they measure quite different viral pools. As such, it is not clear how a comparison of vOTU richness (l. 111-121; 225-238) and overlap (fig. 2) should be interpreted.

To your first point about the methods comparison, we had similar a-priori expectations of which viruses each of the preparation methods would recover and that the parallel methods would generate distinct results. The main intention of the work was to test these assumptions and characterize how these various preparation methods could be redundant or complementary and thus provide new insights into soil viral ecology. To clarify this in the text, we have added a table in the Discussion that describes our a-priori expectations (alongside article references on why we had those assumptions) and how these were or were not what we observed in our results. In addition, we extended our analysis to evaluate how these methods compare to the “standard” methods of Total and Virome DNA / RNA and how their use could influence interpretation of soil ecology. While two other papers have compared Total DNA and Viral (size-fractioned) DNA methods (https://pubmed.ncbi.nlm.nih.gov/33612831/, https://pmc.ncbi.nlm.nih.gov/articles/PMC12697348/), a similar evaluation has not been published for Total RNA and Viral RNA methods. Further, we have added in a separate

approach (i.e., detection-based comparisons, see general response to reviewers and lines 126-137) to more robustly evaluate the differences between the preparation methods.

If you have specific suggestions on how to go about more robustly validating these, please let us know and we will try and accommodate.

Your point about moisture is addressed specifically below.

At the same time, with the exception of limited evidence from predicted hosts, there is little validation that different target pools have been successfully selectively sequenced. It doesn't seem appropriate to consider differences between the fractions to reflect bias (l. 155). Agreement between these methods does not seem to provide information about their methodological validity, since ecologically any outcome would be possible depending on the (unknown) distribution of viral taxa across these different pools. As such, while the comparison is certainly interesting, it lacks a solid theoretical basis to make be rigorous.

A key message we aim to make in the paper is that the specific research question should motivate which preparation method is used. In service of this, we report on the similarities and differences in recovered vOTU quantity and quality, viral community composition, and predicted host composition across the 7 methods included. We do not intend to make arguments about methodological 'validity' per se, since as you point out, the true distribution of viruses is unknown. We have extensively rewritten the discussion and reformatted our figures to make the outcomes of our analysis clearer. As part of these revisions, we have compiled a table (Table 1) of a priori expectations about the subset of the soil virosphere captured by each method (perhaps analogous to the 'target pools' you mention) and a brief synopsis of observed results, highlighting where each method supported or diverged from expectation. In addition, we note that the material used to generate data for each parallel preparation method was derived from the same mass of homogenized soil (instead of from independent soil cores collected within the same field plot) to mitigate contributions of fine scale spatial heterogeneity (Fudyma 2024) to observed similarities and differences across methods.

We are open to suggestions for implementing a more rigorous theoretical basis for this evaluation.

The study reported interesting trends and some significant differences between samples of differing moisture content. The analysis of soil moisture was problematic, however. The sampling site was a field experiment with irrigation treatments (l. 408-417), and the sampling design used these as treatments (Fig. 1) but in the end the analysis ignored this experimental design and it seems that field moisture at the time of sampling is instead used to re-classify samples on a categorical moisture gradient (l. 659-661), apparently

disregarding the experimental moisture treatment or sampling date. The reasons for this are unclear. Crucially, however, this shifts the study from an experimental design to an observational one, where differences may be correlated to moisture content but not necessary caused by it. As just one example, I wonder whether the observed differences are not also related to soil organic matter, which quite likely correlates to field moisture (in as far as this is not driven by experimental irrigation). This undermines causal conclusions about the role of moisture (l. 358-359).

We acknowledge the points you have raised and agree that this is correlative rather than causal investigation. We have modified the text to ensure that language is consistent (i.e., our usage of "related to moisture" or "trending" with moisture), specifically in the results and discussion sections that relate to the relationship between viral communities and moisture (see Lines 248-296, Lines 317-470). We also have toned down the conclusions of the manuscript, specifically with regards to the impact of moisture on viral communities. Please let us know if you have feedback on these changes.

The manuscript has a tendency to show significant results while placing non-significant results in the supplementaries, but this risks a subtle publication bias. For example, determining viral richness under contrasting moisture was a major target for this study, but these results are not shown in the main text (although described in l. 226-238).

We agree that more results should have been included in the main text figures. We have substantially revised all our figures, specifically those relating to diversity and expression trends in response to moisture, to show all statistics regardless of significance. Further, we have specified p-values for all statistical analyses that were done in in each figure. We also note that we changed our analyses to consider measured moisture as a continuous variable for regression analyses rather than the moisture level categories.

l. 243 What is holistic about this approach?
Our extensive revisions to the manuscript have eliminated use of this term.

l. 236 This conclusion is unjustified based on the data. If methods did not detect statistically significant differences, then they should not be considered to have detected differences. Furthermore, this statement seems based on an a-priori assumption that the hypothesized differences were present. Perhaps they were not.
We have modified our statistical approaches and how we report our results. We have re-written these sections to be more specific as to which methods detected which trends using linear regressions.

l. 53 Virus strategies are more diverse than this simple binary classification

We were highlighting lysis and lysogeny, specifically, as they are the predominant lifestyles. We have added in "chronic, etc." per your comment.

l. 55 Language is odd here: can an organism "exist at an abundance" or "exist at lower biomass"?

Text has been revised to clarify that some organisms are less abundant in natural systems, as such can be harder to sequence.

l. 59 Unclear what "them" refers to

We have reworded "them" as "soil microbiomes".

l. 65-66 Viruses are not examples of cells

We have replaced with the word "groups".

l. 66 Separation methods don't maximize the quantity present –as a general rule there's a trade-off between separation purity and total recovery

Thank you for bringing up this point. Text has been edited to emphasize optimization and enrichment.

l. 85 The authors emphasize the need to generalize previous observations across different soil types, but then this study looks only at a single site. Therefore, this doesn't seem to be a gap that is filled by the study.

We have changed the wording here to be more general (see Lines 75-77). We have also constrained our language to make it clear that our study focuses on a single field site and interpretations should be taken with caution (see Lines 464-470, Lines 490-495).

The study uses the term "community" very loosely, for example l. 96 "less studied viral communities" actually refers to operationally defined groups of viruses within the same soil microbial community.

The definition of a community is a group of different populations that live and interact within the same geographic area. As such, community is an appropriate term to call the diverse collections of viruses assayed here. If you have a specific recommendation as to different language please let us know.

Basic soil data is lacking: quantitative SOC content, quantitative texture, WRB soil type classification, land use and plant cover prior to establishing the experiment.

Basic characterization of the soil and history of this study site has previously been published and is now cited in the manuscript.

l. 415 Was water supply adjusted to maintain the soils always at these moisture contents? Percentages of field capacity don't provide a reproducible water supply rate, so this unit of

description is ambiguous.

A consistent irrigation treatment plan was applied to the field site from April to October each year. The plan was not adjusted to maintain a certain soil moisture. The irrigation application did not manifest in consistent soil moisture levels between replicate plots. For this reason we decided to analyze soil by measured moisture content rather than by treatment. Across seasons moisture content of plots was consistent (i.e., lower moisture plots in Fall were also lower in Spring). Percentage of field water capacity is a standard measure of soil moisture.

l. 438 Conventionally, soil moisture content should be calculated relative to soil dry mass, not wet mass.

Here we are referring to the irrigation treatment (i.e., the target soil moisture level), not the actual soil moisture content. Soil moisture reported in the manuscript was gravimetric water See line 506 and Line 529.

l. 459 Greek letter mu should be used in the “micro” unit

Thanks for your attention to detail. We have changed per your request.

l. 469-472 These sentences seem to repeat each other

Thank you for catching this error. You are correct and we have removed the repeating steps.

l. 569 DNA?

We have added this to Lines 634-635.

Methods should also provide the raw sequencing depth for each dataset

All sequencing data is available in Table S1. We have added text to lines 586 and 621 to point readers to this data.

l. 159 and elsewhere, this is a misuse of “n”. This conventionally refers to a number of replicates, but the additional vOTUs added to the database (in this example) are not replicates in the context of this analysis. As such, the “n=” seems to add confusion rather than clarity here.

We have modified our use of “n” throughout the manuscript.

l. 198-199 This sentence is not so clear. What is a “host-phylum linkage profile”? And why is this analysis done at the phyla level? (I would argue if host linkage is only consistent at the phylum level, this is not consistent at all).

We have modified this and changed it to “predicted host profiles” (see Line 220). This approach, aggregating at the phyla level is well established in the literature. For examples see:

https://www.frontiersin.org/journals/microbiology/articles/10.3389/fmicb.2022.946070/full, https://www.sciencedirect.com/science/article/pii/S0960982223008163?via%3Dihub, https://www.biorxiv.org/content/10.1101/2025.11.25.690293v1,

The host linkages are not consistent at the phylum level across our preparations. We state that only 32% of host assignments are detected across all methods (Line 220), and even then, that there is significant variability in the proportions of those assignments within each fractionation method (Lines 224-232).

Fig. 6 Actual p values should be provided rather than just the $p < 0.05$ cutoff.
Per your request, we have modified all figures in the main text and supplemental to include the full p-values for each statistical comparison. We note, however, that for accessibility to the reader, we have kept cutoffs in the main text.

Fig S5 should show individual datapoints, like the figures in the main text.
This analysis has been redone as regression(see now Figure 9, figure S5 and Figures S6).

l. 287-288 which biological insights are these?
We agree this was unclear. These sections have been significantly revised and as such no longer include this language.

l. 342-344 The argument is not so clear why lower diversity would suggest a “trove” of sequences.
We agree that this argument was not robust. We have significantly revised the discussion section and no longer include this language.

l. 383 This is highly speculative, given that we know little about moisture-level responses of viral communities and the relevant data (water-filled pore space and connectivity) is not reported for the soil. The cited Bickel and Or (2020) consider climatic water content, integrating long-term selection processes at a site, and it is not clear how this could be compared to the snapshot of moisture content applied in this work. At least, this comparison is not explained or justified.
We agree that this is highly speculative. We have significantly revised the discussion section related to moisture. We have added very specific text as to how our hypotheses are highly speculative and require further research (Lines 464-470).

Reviewer #3 (Remarks to the Author):

Rodrigues-Ramos et al report a comparison of the different methods that can be used to sequence the soil virome, with a particular emphasis on fractionation and RNA viruses

sequencing. Sampling was done at 7 different locations in the same field, which varied in soil moisture.
According to their analysis, at the species level, there was barely any overlap among the viruses found with the various fractionation methods. However, at taxonomic ranks above family, among RNA viruses, some overlap existed. They also showed that ~ 40% of their DNA vOTU are new, compared to those present in soil virus databases. They finally determine, thanks to their RNA and DNA sequencing of the same samples, the amount of active viruses, defined as those having transcripts, and found it to be very low compared to the amount of viral genomes (but this could be due to technical problems). Nevertheless, on this small dataset, a tendency of higher viral activity in soils with higher moisture was detected.

For me, as a virome- but not a soil-specialist, this analysis is very methodological and technique oriented, and it is not bringing much novelty. However, soil viromes are notoriously difficult to analyse, and I can understand that this paper is a breakthrough in its field.

Thank you very much for your feedback and for recognizing the importance of our work. We appreciate the time that was spent giving us actionable feedback. We include line-by-line responses below, but given the substantial revisions the manuscript has undergone, we ask you please read the new, improved manuscript.

If the editor wants to accept it, I would recommend to improve the methods in the following directions:

-Clustering is made in two steps (lines 105 and 241), one of low quality, that serves to conclude for all results in figures 2 to 5, one of higher quality, that serves to conclude in figure 6. The high quality clustering should be used all along. Then to define which vOTU is present in which sample (for the important figure 2, for instance), a mapping step should be applied (using rarefied RNA,and rarefied DNA reads to compare samples at the same sequencing depth), including the removal of vOTU that collected insufficient reads (the cut-off 75% coverage and 1x depth is OK). The main difficulty with figure 2 as it is, is that some vOTU may be present in two or more samples, but assembly was not good enough to get it complete (as reflected in the CheckV result which is quite poor). So fragments 1 and 2 of the same vOTU appear as independent vOTU, and the overlap between methods is poor. This problem should be mentioned.

Thank you very much for this feedback! We had not considered your excellent point regarding the same virus being present in two samples yet not being able to dereplicate into

each other. In response to this comment, we have analyzed the data using your suggested approach. Specifically, we use the final, globally clustered database and map the reads from each of the preparation methods (e.g., Tot DNA, Vir DNA, etc.). We refer to this as viral "detection", i.e., the ability of a method to contain + map reads that belong to a specific viral contig. (see Line 126). We note, however, that the original approach (which we are now calling "recovery-based" approach) is also important for a reader, as in most cases researchers will only have one or two preparation approaches to choose from due to financial constraints. Specifically, this recovery-based approach answers the question: "If I could only pick 1 method, which will give me the best results" while the "detection-based" approach answers the question: "If I wanted to maximize the most viral sequencing to map to a reference database, which method would give me the best results". We have ultimately decided to include both approaches in our final analyses (Lines 115-137). Please let us know if you have any further feedback.

-Fig S2D indicates that the vOTU set is collecting a small fraction of the total reads used for assembly. This should be mentioned as a limit of the study, and commented. Could it be that a better assembler (such as Spades) would have given better results?
We have rewritten the results to acknowledge that this is indeed a limitation of this study (Lines 147-151). While we agree that SPAdes could potentially have given different (and potentially even better) results, the point of the manuscript was to compare the methods using a standardized pipeline. Ultimately, we chose MEGAHIT because it is a commonly used assembler and for its accessibility to more users because it is much more memory efficient than SPAdes.

-Does the increase in vOTU richness with soil moisture simply reflect an increase in bacterial OTU (bOTU) richness? Authors have the sequencing data to analyse bOTU (but this analysis is not reported in the manuscript), if they do not intend to report this analysis, they should at least mention this interpretation.
This could very well be a possible interpretation for this data, especially when we consider the BAr DNA method which should, for the most part, select for integrated prophage. We have added text in Line 440 that states this is a possibility, and likely, a reason as to why we see such patterns.

-The Methods section 'nucleic acid extraction' should more clearly describe what is the "VirFrac" fraction, the same way as "BarFrac" and "FloatFrac" are described.
We have elaborated on the specifics of DNA and RNA viral extractions in our methods line 554. We have also added in Table 1 into the discussion, which describes the a-priori expectations of each method based on published literature as well as how that compares to what we saw a-posteriori in our results. Finally, we have added in a conceptual diagram

(new Figure 1) that shows how each preparation method was generated.

Reviewer #3 (Remarks on code availability):

The git hub contains raw data used to build up phylogenetic trees of RdRp, as well as parsing scripts.
Thank you for confirming that all code was publicly accessible to readers. We have further modified this code to reflect the latest changes made to our manuscript.

REVIEWER COMMENTS ROUND 2

Reviewer #1 (Remarks to the Author):

The manuscript was greatly revised and many of my concerns were addressed. The authors improved the description of the work performed that (from my understanding) was done thoroughly. Some of the bolder claims were toned down, which improved the data interpretation. The manuscript remains very methodological and descriptive (a bit like a technical note) and I cannot fully appreciate the novelty. I can understand the challenges faced for recovering viral RNA/DNA and that this "groundwork" is required to advance the research on viruses in soil. While the manuscript provides a relevant and useful dataset, it is missing an impactful experimental design and outcome. Overall, the text requires language editing because some parts are difficult to follow and the text could be more engaging. While the manuscript and figures improved, there are several points to address.

Title: The title does not say much and feels like it is just stating the obvious: "if you measure different things you may arrive at different interpretations"

We have crafted a new title that highlights that our work informs on how to choose an appropriate method given a specific scientific question.

Abstract should be re-written.
L14: "[...] microbial community [...]" and remove "communities" before "of [...] viruses". Maybe a bit philosophical: Communities are interacting populations and I would not know how this applies to viruses, which require a host for interaction.

Although viruses depend on hosts for replication, it is common in the literature to discuss viruses in terms of "viral communities." Regardless, we have modified per your request, so that L13 now reads: "To enhance detection of DNA and RNA viruses..."

L19-21: revise sentence

We have revised this sentence for clarity and to correct a typo (L18). It now reads: "DNA viromes outperformed total soil metagenome in viral detection and quality. Contrastingly, DNA viromes and total soil metatranscriptomes performed similarly for viral recovery, though RNA viromes yielded higher quality genomes."

L24-26: revise sentence. "[...] expression by moisture [...]"? "[...] and method [...]"? I cannot follow what is written here. What is/are the object/subject of the sentence?

We have revised this sentence to clarify and shift the focus from moisture to vOTU quantity/quality (L21). It now reads: “Together, our results highlight how different preparation methods can influence the recovery and quality of DNA and RNA vOTUs.”

L68: What are "Viral DNA and RNA omics"? metagenomics? metatranscriptomics?

We have clarified by re-writing “omics” as “metagenomics and metatranscriptomics.” (L68)

L76: Soil texture is mentioned here although the study is based on a single soil... this should be revised.

We have revised this sentence to clarify that we are calling attention to the importance of considering site-specific properties in studies of the soil virosphere and to advocate for studies across different soil types (L75). This sentence now reads: “However, additional studies examining different soil types are needed to understand how viral and microbial communities change across soil characteristics like texture and moisture level.”

L78: What is a "site-scale" approach? A site has no representative scale. It can be 1, 100, 1000, ... m2

“Site-scale” is a common way to describe a study at a specific, relatively small, well-defined location, and we used the term to communicate that we evaluated samples from plots across a single site, but not across sites. We have further clarified this sentence to read: “Here, we sampled a single site with 7 plots (19.5 m^2) to...” (L79).

L90: The header has numbers. It is not clear what is the importance of this. The header does not convey a message and is very descriptive.

We have revised this header to better communicate the key result. It now reads: “Parallel soil preparation methods yield unique vOTU profiles.” (L92)

L113: This seems overstated and should be rephrased. Recovery implies that we know what is there and are able to reconstruct the composition quantitatively. To address this

one would need to spike the soil with a known mixture of viruses and then recover it from DNA/RNA extractions. Here the outcome is obvious and boils down to "different methods measure different things" (L120).

We have removed this header and consolidated this results section with the section above, under the revised header “Parallel soil preparation methods yield unique vOTU profiles.” To avoid confusion, we specifically define **recovery** in the text as “the ability to assemble vOTUs ≥10kb from a sample” to distinguish this approach from **viral detection**, which we later define as “the ability to detect a virus in a sample by the mapping of reads to a reference viral database”.

Table 1: This is a useful addition.

Thank you for this feedback.

L429: The header should be rephrased. The database does not show anything - it is just data. Moisture cannot have a relationship with ecology - ecology is the study of relationships among living organisms and their environment while moisture/water content is the state of the system. Maybe "insights into viral ecohydrology" or "relationship with the ecological system". Also, it is important here that the grassland is managed.

We have revised the header as suggested for precision. It now reads: “Virus profiles from managed grassland provide insights into viral ecology.” (L418)

L448: The range of moisture levels are very low (I know studies can blow up minor differences and find significance with enough data). For context, this could be the difference in water contents of a soil aggregate and the surrounding loose soil. The discussion is fine as it states the limitation, but it should be called a "trend" rather than "marginally significant".

Thank you for this suggestion. We have revised this sentence accordingly and have framed the results and discussion more broadly to speak to a “positive trend” as requested.

L482: here the values are 11-18%, but earlier it was 11-17%
Thanks for catching this inconsistency. And thanks to your comment below, we’ve identified and corrected an error in our gravimetric water content calculations. The correct moisture range is 12-20%. This correction is also reflected in revised statistical analyses

and figures. We note this correction did not qualitatively impact the results or interpretations.

Additional comments on previous comments of reviewer #2:
The study reported interesting trends and some significant differences between samples of differing moisture content. The analysis of soil moisture was problematic, however. The sampling site was a field experiment with irrigation treatments (l. 408-417), and the sampling design used these as treatments (Fig. 1) but in the end the analysis ignored this experimental design and it seems that field moisture at the time of sampling is instead used to re-classify samples on a categorical moisture gradient (l. 659-661), apparently disregarding the experimental moisture treatment or sampling date. The reasons for this are unclear. Crucially, however, this shifts the study from an experimental design to an observational one, where differences may be correlated to moisture content but not necessary caused by it. As just one example, I wonder whether the observed differences are not also related to soil organic matter, which quite likely correlates to field moisture (in as far as this is not driven by experimental irrigation). This undermines causal conclusions about the role of moisture (l. 358-359).

>R#1: While the authors toned down the references to soil moisture, it is still prominently presented in the abstract and in figures. Removing the reference to differences between expression/DNA in the abstract would be better. Especially, because the trends do not seem fundamentally different given the uncertainty of the data. It is not surprising that "expression and diversity" should yield comparable results in the first place. Also, Fig 8/9 could be reduced (keep only few panels for illustration purpose and move the rest to SI) without "hiding" the lack of clear relationships.

We have further toned down our interpretation of moisture-driven trends in diversity and/or expression by removing mentions of moisture from the abstract. The abstract is now focused on the differences across parallel preparation methods in terms of viral recovery efficiencies.

We originally expanded on Figures 8 and 9 at the request of Reviewer #2, who expressed concern that we were biasing our results by only displaying significant results (see comment below this one). As such, we have decided to leave the figures as they are (except for revising to incorporate correct GWC values).

The manuscript has a tendency to show significant results while placing non-significant

results in the supplementaries, but this risks a subtle publication bias. For example, determining viral richness under contrasting moisture was a major target for this study, but these results are not shown in the main text (although described in l. 226-238).

>R#1: As mentioned, I am not sure putting too much emphasis on the moisture results is helpful, because there is not much conceptual interpretation. E.g, L358: "unique ecology that mirrors their bacterial hosts " is mentioned as an explanation, but does not provide any conceptual link. Why should bacteria (and their viruses) be affected? While I agree with the later interpretation on how soil moisture can affect the ecosystem (L434ff), it is not explained how this relates to viruses - e.g., how does the habitat/host distribution change and affect the viral transmissions, etc. - and this only appears later in the text (L456ff). Also, the discussion often repeats the results. I suggest shortening the discussion on moisture by combining the sections.

We appreciate your feedback on how to improve the discussion. We have consolidated all text related to moisture into single sections in the Results and the Discussion, streamlining how we address moisture in this paper. Specifically, we have removed these two sentences from the discussion: "We highlight that the BAr DNA showed highly significant positive trend between richness and moisture, while also having a negative relationship between Pielou's evenness and moisture. This could suggest that the viral communities of the BAr fractionation have unique ecology that mirrors their bacterial hosts given their integrated lifestyles," to better focus this paragraph on viral lifestyles (e.g., integrated vs. free viruses).

Basic soil data is lacking: quatitative SOC content, quantitative texture, WRB soil type classification, land use and plant cover prior to establishing the experiment.

>R#1: The soil characteristics should be briefly reported in the text (not just adding the citation) as these are important for understanding the context of eventual moisture-virus relations.

We agree and have added information regarding SOC, WRB soil type, and plant cover in the Methods (Line 473-486). The methods now read: "T Samples were collected from the Tall Wheatgrass Irrigation Field Trial in Prosser, WA, USA (46°15'04"N and 119°43'43"W), operated by Washington State University, and described in our previous publications [60–62]. The site is characterized by marginal Aridisol soils with low organic matter content (<2%), pH of 8, and a sandy loam texture (55.5% sand, 34.1% silt, 10.4% clay). Specifically,

it is described as coarse-silty, mixed, superactive, mesic Xeric Haplocambids and having high porosity, permeability, and soil bulk density (avg = 1.56g/cm^3) [58]. Tall wheatgrass (*Thinopyrum ponticum*), which is drought tolerant and adapted for growth on marginal soil, was established in May 2018, prior to which the site was uncultivated desert shrub-steppe. Plants are uniformly distributed within plots. Irrigation treatments have been ongoing since spring 2019. Irrigation is provided through drip lines from April to October with water supplied at four levels (100%, 75%, 50%, and 25% field water capacity) to create plots with differing water stress based on modeled crop evapotranspiration of tall wheatgrass [63] . Each experimental plot is 2.1 m × 10.7 m with a 1.5 m alley between adjacent plots **(Figure S1)**."

l. 415 Was water supply adjusted to maintain the soils always at these moisture contents? Percentages of field capacity don't provide a reproducible water supply rate, so this unit of description is ambiguous.

>R#1: It is called "water content at field capacity" and quantifies how much (capillary) water is held by the soil against gravitational (free) drainage. It is an often reported quantity that, however, strongly depends on the methodology used to determine its values. Here, not enough information is provided to assess what the numbers mean.

We have added citations for the method used to determine field capacity from our collaborators that manage the field site (L489-491). (https://doi.org/10.7273/000006916, https://irrigation.wsu.edu/Content/Fact-Sheets/Soil-Monitoring-and-Measurement.pdf)

l. 438 Conventionally, soil moisture content should be calculated relative to soil dry mass, bot wet mass.

>R#1: For soil, it should be WC (w/w) = m_water/m_dry = (m_fresh-m_dry)/m_dry

Thank you for catching this calculation error. We have generated correct values, re-run affected analyses, and re-generated affected figures (Figures 8, 9, S6). Importantly, this correction did not qualitatively impact the results or interpretations.

l. 383 This is highly speculative, given that we know little about moisture-level responses of viral communities and the relevant data (water-filled pore space and connectivity) is not

reported for the soil. The cited Bickel and Or (2020) consider climatic water content, integrating long-term selection processes at a site, and it is not clear how this could be compared to the snapshot of moisture content applied in this work. At least, this comparison is not explained or justified.

>R#1: This was addressed.

Reviewer #3 (Remarks to the Author):

I am afraid my first and main methodological point has not been fully answered. The authors have performed read mapping on the consolidated database, as requested, but they did not perform the appropriate step of read rarefaction (they instead made a normalization per sequencing depth, which is different). To compare vOTU richness between samples, a clean rarefaction is important.

Here how to proceed: Rarefaction consists in the a posteriori and proportional reduction of the number of mapped reads per sample, to reach an equivalent number of mapped reads in all samples. This rarefaction and richness estimation is done after having discarded, in each sample, the vOTUs insufficiently covered (as you did). As an example: If the “poorer” sample has a total of 13455 reads mapped on all vOTUs, then all other samples should have their read numbers per vOTU reduced proportionally so that in total they also have 13455 reads mapped. And vOTU ending up having less than 1 read after rarefaction should not be counted in the richness estimate. The number of rarefied reads chosen used to compare all samples should be stated in the Methods. The Y axis unit in Figures 2B and 3B should display the number of vOTU found in each sample, so values above 1 are expected (numbers such as 10-4 or 10-5, as shown now, are not relevant).

The debate about whether it is appropriate or not to rarefy sequencing data prior to microbiome analyses is ongoing[1,2], and both methods are scientifically reasonable. Rarefying to a common number of mapped reads is indeed appropriate when the goal is to compare richness across samples at an equalized sampling effort. In our case, however, the purpose of these figures is to evaluate the overall performance of each preparation method, as implemented in this study, at detecting the vOTUs present in the consolidated database.

Our preparation methods differ significantly in extraction and enrichment strategy, ultimately affecting the resulting proportion of viral reads and sequencing depth. These differences in viral yield and depth are intrinsic aspects of method efficiency. Rarefying all methods to the same number of mapped reads would simulate a scenario in which each method achieves identical viral sampling effort, which is infeasible for lower yield methods like DNA and RNA viromes. Further, rarefying across different soil preparation methods essentially treats all samples as technical replicates from the same sequencing library, which is not the case. Thus, we opted to control for differences in depth by looking at the normalized number of viruses detected (i.e., total number of viruses detected per the total sequencing depth of a specific method). This approach was adapted from that of Ma et al. 2024 where they normalized viral diversity by number of base pairs sequenced (https://www.nature.com/articles/s41559-024-02347-2).

We have revised the text to articulate the logic motivating our decision to not use rarefication. Lines 121-125 now read: "Because sequencing depth and the percent fraction of viral reads per sample are themselves outcomes of each preparation method, we did not rarefy our reads to a common sequencing depth, instead we opted to normalize the number of vOTUs detected to the total sequencing depth from each method [21]." Also, we have modified the legend in Figure 2 and 3 to reflect the normalization strategy that was used.

(1) McMurdie, P. J. and Holmes, S. Waste Not, Want Not: Why Rarefying Microbiome Data Is Inadmissible. PLoS Comput Biol 10(4): e1003531 https://pmc.ncbi.nlm.nih.gov/articles/PMC3974642/

(2) Schloss, P.D. Waste not, want not: revisiting the analysis that called into question the practice of rarefaction. mSphere 9(1). https://doi.org/10.1128/msphere.00355-23.

In the text itself, the sentence line 128-131 is obscure, and probably inaccurate. It should be corrected. It states: "Contrary to the recovery-based analyses, this is less stringent approach that does not require the assembly of a full virus and can ultimately help determine whether the reported recovery differences between methods shown in Figure 2A and Figure 3A are due to enrichment of certain vOTUs or incomplete contig reconstruction between methods.": Why is this less stringent? It is on the contrary more sensitive. What is meant by "does not require assembly of a full virus"? Virome studies are never limited to the subset of full viruses (this would be a very low number of vOTUs). What I tried to explain in my former review was that by combining assembly results (which are always quite deficient, regardless of the tool used), you increase the quality (the length) of the contigs retrieved. The consolidated database and rarefaction approach is the less incorrect

method permitting to compare samples/methods of extraction. What you call the "recovery based" analyses are highly biased by the sequencing depth: a given sample sequenced twice, with a 10-fold sequencing depth difference, will give artificially different richness results.

Thanks for this additional feedback. Our language was unclear. Yes, we agree that the virus recovery-based method we employed (that is, assembly of a virus that is at least 10kb in length) is directly influenced by sequencing depth and the inherent limitations of any method implemented (as addressed in the above comment) and is the point of our study. This approach answers the question "If you only have resources to apply one preparation/sequencing method, which method yields more, higher quality viral data?" By comparison, the read mapping approach requires that you already have an appropriate viral reference database, which is rare for most systems since new viruses are discovered practically with each new investigation[1,2,3]. As such, we assert that both approaches are useful but evaluate distinct questions.

To address these concerns, we have removed the word "full" from this text, as we were simply referring to a virus that is at minimum 10kb in size. We have revised the wording in this section to remove claims about stringency as well. The text at lines 132-135 now reads: "By combining viruses from all methods into a single, consolidated vOTU database, we mitigate some of the deficiencies of individual assemblies (e.g., fragmented contigs) and improve our ability to detect shared viruses across methods via the mapping of 151bp reads. In this way, we can assess whether the apparent differences in recovery (Figure 2A, Figure 3A) primarily reflect true enrichment of vOTUs or are influenced by incomplete contig reconstruction in some methods."

(1) Neri, U., et al. Expansion of the global RNA virome reveals diverse clades of bacteriophages. Cell. https://doi.org/10.1016/j.cell.2022.08.023

(2) Lee, B. D., et al. Mining metatranscriptomes reveals a vast world of viroid-like circular RNAs. Cell. 186(3): 646-661. https://doi.org/10.1016/j.cell.2022.12.039

(3) Graham, E. B., et al. A global atlas of soil viruses reveals unexplored biodiversity and potential biogeochemical impacts. Nature Microbiology. 9: 1873-1883. https://www.nature.com/articles/s41564-024-01686-x

REVIEWER COMMENTS ROUND 3

Reviewer #4 (Remarks to the Author):

Thank you for reaching out regarding Reviewer 3's suggestion on rarefaction. I have given this careful thought and wanted to share my assessment.

In my view, the rarefaction approach proposed by Reviewer 3 that suggests normalizing samples based on the number of reads mapped to the consolidated database rather than sequencing depth is not necessary for the purposes of this manuscript. This approach would effectively standardize comparisons around the viral fraction of the data, thereby focusing the analysis specifically on viral populations. While such normalization is appropriate when the goal is to make ecological inferences about viral communities, it does not appear to align with what authors are trying to demonstrate.

As I understand it, the authors' main point from this particular analysis is to demonstrate that viral enrichment leads to an increased recovery of viral sequences, an expected but still useful result (Figure 2B and 3B; line 116-127). Applying the suggested rarefaction would not fundamentally change this conclusion, as the enrichment effect is likely to remain strong. However, it could alter relative comparisons among the different sample types (e.g., Euk,Tot, and Bar). Since the manuscript does not appear to draw specific conclusions from these relative differences (though I recommend confirming this), the additional analysis may not provide meaningful new insight.

For these reasons, I believe the current analytical approach is sufficient to support the authors' conclusions, and the additional rarefaction step suggested by Reviewer 3 is not essential.

Also, note that I did not review Figure 8 and its conclusions. If R3 is worried about that part, where they seem to draw ecological inferences, the ask might be valid.

We thank the reviewer for their prompt response and for taking the time to review our manuscript. We also appreciate that the reviewer recognized that our non-rarefied approach is valid considering the goal of this study: to demonstrate that viral enrichment leads to increased recovery of viral sequences, and that this viral enrichment varies across methods. We have added language to further clarify our choice not to rarefy: Line 143 now

reads: “Because sequencing depth and the percent fraction of viral reads ***(i.e., the enrichment of viral particles)*** ...

We thank the reviewer for bringing up a possible caveat regarding Figure 8 for which we do, in fact, attempt to “infer” some degree of ecology. However, the purpose for Figure 8 is similar to the purpose of Figure 3. We ask the question: “If we only had a single one of these methods, would our interpretation of viral patterns change?”. To this end, we have added in a few lines of text to reflect the purpose of our analyses in Lines 286-289.

We also note that, in response to Reviewer #3, Reviewer #4, and the request of the editor, we have included a paragraph in the Discussion related to the decision to not rarefy the data for our analyses.

Reviewer #3 (Remarks to the Author):

The authors did not comply with my first request. They instead provide reference to an old debate about rarefaction of 16S-based assessments of OTU richness, which was also semantic, and was apparently solved in favour of rarefaction.

Anyway, this debate is irrelevant with the method I proposed to apply, a method used in viral metagenomics and taking into account the fact that total DNA approaches will contain a large proportion of bacterial reads which should not be considered when comparing methods.

Indeed, in the soil samples analyzed here, in average 19,6% of the “viral extraction” reads mapped by to the (consolidated?) viral DB, whereas only 0.1% of the “total DNA” reads mapped on this DB (if suppl table 1 “read_mapping_data” sheet is providing mapped reads against the consolidated DB).

Using the method I proposed in the last round of review would probably have revealed a better performance of the total DNA approach.

We reiterate that a central aspect of our work was to investigate the quality and quantity of information about the viral community that each different preparation technique provides. The varying amount of viral sequence recovered, relative to other methods, is an essential factor in selecting an approach that is appropriate for a specific research question. We make no assertions that one technique is more correct than another. Please see Reviewer #4’s comments, which support our approach.

The answer to my second comment is confusing and not satisfactory. The authors suggest that to apply a read mapping approach, you need to have first a reference viral database, which is incorrect. You can also do read mapping on de novo databases constructed by read assembly, as they have done here. They continue by saying that both approaches are useful, which is very confusing.

We seem to be arguing the same point. Read mapping does require a reference to search against. That reference can be something amassed from previous studies (such as the Global Soil Virus Atlas) or generated from de novo assembly of the sampled read sets (as we did). One issue with using an existing reference database is that, with the high diversity of viruses, the viruses represented in the reference might not overlap well with those present in the sample, giving poor results. A de novo assembly should provide a more congruent set of target sequences, but this approach is only possible if sequence collected is sufficient to provide good assembly of viral genomes.

Finally, to clarify, our statement that 'both approaches are useful' refers to the viral recovery (assembly-based) analysis and the viral detection (read-mapping-based) analysis.
